# Reconsidering evidence for psychedelic-induced psychosis: an overview of reviews, a systematic review, and meta-analysis of human studies

**DOI:** 10.1038/s41380-024-02800-5

**Published:** 2024-11-27

**Authors:** Michel Sabé, Adi Sulstarova, Alban Glangetas, Marco De Pieri, Luc Mallet, Logos Curtis, Héléne Richard-Lepouriel, Louise Penzenstadler, Federico Seragnoli, Gabriel Thorens, Daniele Zullino, Katrin Preller, Kerem Böge, Stefan Leucht, Christoph U. Correll, Marco Solmi, Stefan Kaiser, Matthias Kirschner

**Affiliations:** 1 Division of Adult Psychiatry, Department of Psychiatry, University Hospitals of Geneva, 2, Chemin du Petit-Bel-Air, CH-1226 Thonex, Switzerland; 2 Faculty of Medicine, University of Geneva, Geneva, Switzerland; 3 Univ Paris-Est Créteil, DMU IMPACT, Département Médical-Universitaire de Psychiatrie et d’Addictologie, Hôpitaux Universitaires Henri Mondor - Albert Chenevier, Assistance Publique-Hôpitaux de Paris, Créteil, France; 4 Sorbonne Université, Institut du Cerveau—Paris Brain Institute—ICM, Inserm, CNRS, Paris, France; 5 Department of Mental Health and Psychiatry, Global Health Institute, University of Geneva, Geneva, Switzerland; 6 Young Adult Psychiatry Unit, Geneva University Hospitals, Geneva, Switzerland; 7 Mood Disorder Unit, Psychiatric Specialties Service, Geneva University Hospital, Geneva, Switzerland; 8 Division of Addiction Psychiatry, Department of Psychiatry, University Hospitals of Geneva, 70, Grand-Pré, CH-1202 Geneva, Switzerland; 9 Department of Adult Psychiatry and Psychotherapy, Psychiatric University Clinic Zurich and University of Zurich, Zurich, Switzerland; 10 Department of Psychiatry and Psychotherapy, Campus Benjamin Franklin, Charité—Universitätsmedizin Berlin; and Freie Universität Berlin; and Humboldt-Universität zu Berlin; and Berlin Institute of Health, Berlin, Germany; 11 German Center of Mental Health (DZPG), Berlin, Germany; 12 Medical University Brandenburg—Theodor Fontane, Berlin, Germany; 13 Department of Psychiatry and Psychotherapy, School of Medicine, Technical University of Munich, Munich, Germany; 14 Klinik für Psychiatrie, Psychosomatik und Psychotherapie des Kindes- und Jugendalters, Charité—Universitätsmedizin Berlin, Campus Virchow, Augustenburger Platz 1, 13353 Berlin, Deutschland; 15 Department of Psychiatry and Molecular Medicine, Donald and Barbara Zucker School of Medicine at Hofstra/Northwell, Hempstead, USA; 16 Department of Psychiatry, The Zucker Hillside Hospital and Zucker School of Medicine at Hofstra/Northwell, New York, USA; 17 SIENCES lab, Department of Psychiatry, University of Ottawa, Ottawa, ON Canada; 18 Department of Mental Health, The Ottawa Hospital, Ottawa, ON Canada; 19 Ottawa Hospital Research Institute (OHRI) Clinical Epidemiology Program University of Ottawa, Ottawa, ON Canada; 20 School of Epidemiology and Public Health, Faculty of Medicine, University of Ottawa, Ottawa, ON Canada; 21 Department of Child and Adolescent Psychiatry, Charité Universitätsmedizin, Berlin, Germany

**Keywords:** Schizophrenia, Neuroscience, Depression

## Abstract

**Background:**

Persons with schizophrenia are excluded from psychedelic-assisted therapy due to concerns about the risk of triggering or worsening psychosis. However, there is limited meta-analytic data on the risk of psychedelic-induced psychosis in individuals with pre-existing psychotic disorders.

**Methods:**

We conducted a systematic review, meta-analysis, and overview of reviews to assess the incidence of psychedelic-induced psychosis and symptom exacerbation in schizophrenia. Our pre-registered protocol (CRD42023399591) covered: LSD, psilocybin, mescaline, DMT, and MDMA, using data from Embase, PubMed, PsyARTICLES, PsyINFO, and trial registries up to November 2023. A random-effects model was used to calculate psychosis incidence, with standardized assessments of study quality.

**Results:**

From 131 publications, we analyzed 14 systematic reviews, 20 reviews, 35 randomized-controlled trials (RCTs), 10 case-control studies, 30 uncontrolled trials (UCTs), and 22 cohort studies, most of which were low quality. Meta-analysis of nine studies showed an incidence of psychedelic-induced psychosis at 0.002% in population studies, 0.2% in UCTs, and 0.6% in RCTs. In UCTs including individuals with schizophrenia, 3.8% developed long-lasting psychotic symptoms. Of those with psychedelic-induced psychosis, 13.1% later developed schizophrenia. Sensitivity analyses confirmed the results.

**Conclusion:**

In summary, the reviewed evidence suggests that schizophrenia might not be a definite exclusion criterion for clinical trials exploring safety and efficacy of psychedelics for treatment-resistant depression and negative symptoms. However, given the low quality and limited number of studies, more high-quality research is needed, and a conservative approach is recommended until further data is available.

## Introduction

Supported by the U.S. Food and Drug Administration (FDA) and other funding agencies, the scientific research and interest in the therapeutic potential of psychedelics for treating mental health conditions is rapidly expanding [1]. Significant breakthroughs in psychedelic research provide valuable insights into the mechanisms of mental disorders and offer novel therapeutic strategies for treatment-resistant conditions in depression [2], substance use disorders [3], anxiety [4], and post-traumatic stress disorder (PTSD) [5]. However, specific patient populations have not been considered for psychedelic treatment approaches, such as patients with schizophrenia and bipolar disorders until recently [6], mainly due to concerns about psychedelic-induced acute or long-lasting psychotic symptoms [7]. It thus remains an open question whether psychedelic drugs could have a role in the treatment of schizophrenia, in particular for patients with prominent negative symptoms [8]. Focusing on these observations of psychedelic-induced psychotic experiences, the serotonin hypothesis of schizophrenia proposed that serotonin-2A overactivity could be involved in the pathophysiology of schizophrenia (Vollenweider et al. [9]). However, recent research shows that cannabis and illegal psychostimulant drugs are much more likely than serotoninergic psychedelics to induce psychotic symptoms in users (30-55%) [10, 11]. The serotonin hypothesis of schizophrenia suggests serotonergic psychosis typically results in a reversible psychotic syndrome lasting a few hours, which shares certain similarities with acute decompensation of psychotic symptoms in schizophrenia. However, this serotonergic psychosis is characterized primarily by visual hallucinations and shows no response to D2 blockers, distinguishing it from the dopamine and glutamatergic-related psychosis seen in schizophrenia (Bowers et al. [12]).

Prior to the advent of the serotonergic schizophrenia hypothesis, some researchers in the 50 s and 60 s used lysergic acid diethylamide (LSD) for stabilized patients with schizophrenia (Fink et al. [13]; Hoch et al. [14]).

However, concerns regarding ‘overdiagnosis’ in patients with schizophrenia has been reported which greatly limited these findings [15]. Other serotonergic drugs, such as antidepressant, have shown small effects in improving depressive and negative symptoms in patients with schizophrenia [16]. Therefore, the potential of serotonergic psychedelics to enhance neuroplasticity as seen in animal studies [17] could offer significant promise for patients with treatment-resistant depressive symptoms and negative symptoms [8, 18]; NCT05770375.

In contrast to these hypothetical benefits of psychedelics in the treatment of schizophrenia, the relationship between serotonergic psychedelics and exacerbations of psychotic episodes is unclear. Overall, Cohen suggests a low rate of prolonged psychotic reactions in LSD users (1.8 per 1000) [19]. Accordingly, Strassman proposed the distinction between acute panic reactions resolving spontaneously within a day, prolonged psychotic reactions lasting more than one day, and more complex chronic undifferentiated psychotic reactions [7]. A recent meta-analysis by Murrie and colleagues found a 26% transition rate to psychoses for hallucinogen users, which is lower than the rate associated with cannabis (34%) but not amphetamines (22%) [11, 20]. However, these results are based on only three studies, mainly driven by one study’s weight on phencyclidine (PCP) users. PCP blocks the uptake of dopamine and norepinephrine, leading to sympathomimetic effects, and is a NMDA receptor antagonist provoking dissociative symptoms, which is absent from usual doses of classic psychedelics [21]. Furthermore, recent randomized-controlled trials (RCTs), including patients with depression and PTSD, show the absence of risk of prolonged psychotic reaction following the use of MDMA, LSD, and psilocybin in selected populations free from any history of personal or family risk of psychotic symptoms [22]. Taken together, the effective risk of psychedelic-induced psychosis or worsening of pre-existing psychotic symptoms in schizophrenia -as well as in the early stages of the psychosis spectrum- remains incompletely understood. This uncertainty raises the question whether the current exclusion of patients with psychotic symptoms may be too restrictive and hamper progress in developing novel treatment approaches for negative symptoms of schizophrenia. To shed light on the existing evidence for the risk of psychedelic-induced psychosis and worsening symptoms of schizophrenia, we conducted an overview of reviews, a systematic review and meta-analysis. We focused on serotonergic psychedelic drugs of which some have shown promise as therapeutics in mental disorders and potential candidates for treatment in schizophrenia, including LSD, N,N-Dimethyltryptamine (DMT), mescaline, psilocybin and 3,4-Methylenedioxymethamphetamine (MDMA). Although MDMA has different mechanisms than classic psychedelics with additional action on dopaminergic, and GABAergic systems [23, 24], we decided to include MDMA considering the similar effects on the serotonin system and the therapeutic potential in PTSD [25]. Our primary aim was to summarize the literature regarding the current evidence of the risk for de novo psychedelic-induced psychosis, exacerbation of psychotic symptoms in pre-existing psychotic disorders, and development of schizophrenia after psychedelic-induced psychosis. Our secondary aim was to conduct a meta-analysis of the incidence of long-lasting (>48 h) psychotic symptoms following psychedelic use for each specific population and the risk of transition to schizophrenia.

## Methods

### Registration

This systematic review was conducted according to the Preferred Reported Items for Systematic Reviews and Meta-Analysis (PRISMA) (Supplementary Information 1). The review protocol was registered on PROSPERO in April 2023 (CRD 42023399591). The literature search was updated in November 2023.

### Search strategy

A multi-step literature search was performed in other to conduct a joint umbrella review and a meta-analysis [26]. First, two reviewers (MS, AG) independently reviewed titles and abstracts using Rayyan, a research collaboration web platform for systematic reviews in PubMed, Embase, and PsycINFO using specific search terms (Supplementary Information 1). The only limits applied were human studies. In case of disagreement, full texts were analyzed until both reviewers reached a consensus. Second, two authors (MSa and AG) independently extracted articles using a predefined data extraction form. Third, snowball searches of reference lists were conducted by cross-referencing key papers and other relevant articles identified by the electronic searches, in particular in retrieved reviews.

### Eligibility criteria, hierarchization of the quality of evidence, and data extraction

We included systematic reviews, reviews, guidelines, meta-analysis, RCTs, clinical trials, case-control studies, uncontrolled trials, prospective and retrospective cohorts, and population-survey/register studies that explored the link between psychosis or other psychiatric side-effects following serotoninergic psychedelic consumption (LSD, DMT, mescaline, psilocybin, MDMA). We decided to exclude both cannabis, salvia divinorum and PCP from our analysis, as their mechanisms of action are clearly distinct. Moreover, due to their specific neurobiological effects, cannabis and PCP are strongly linked to the occurrence of psychoses, and salvia divinorum has dissociative properties. Case reports and case series were excluded.

Considering the extensive history of psychedelic research and the expected significant heterogeneity in the quality of evidence, we employed a hierarchy of evidence to identify the ‘best evidence’ by examining all published studies from uncontrolled trials to reviews and guidelines. Firstly, given that the definition of ‘psychedelic (hallucinogen)-induced psychosis’ is not truly established, we considered Strassman’s landmark article on adverse reactions to psychedelics drugs [7], and the definition of substance-induced psychosis according to the DSM-5 to guide our inclusion criteria for identified studies and more recent reviews on the subject [27] (Supplementary Information 2). We distinguish between various groups of psychedelic users on the continuum ranging from ‘healthy’ individuals to patients with schizophrenia: individuals considered as ‘healthy individuals’, patients with other diagnoses (e.g., depression), patients with susceptibility for psychosis, patients with long-lasting psychotic symptoms, patients with a diagnosis of schizophrenia. Additionally, we attempted to assess the temporal relationship between prolonged psychotic reactions and the use of psychedelics whenever possible.

Secondly, two authors (MSa, AG) independently categorized the quality of evidence and the risk of bias by classifying studies based on their type and evaluating their quality using specific assessment tools. We utilized the AMSTAR-2 scale for systematic reviews [28], the SANRA scale for reviews [29], the Cochrane Risk-Of-Bias tool for RCTs (RoB 2) [30], and Risk Of Bias In Non-randomized Studies of Interventions tool (ROBINS-I) for non-randomized studies [31]. To further enhance the hierarchy of evidence, we employed the Oxford Center of Evidence Based Medicine (OCEBM) level of evidence framework [32]. A distinguishing feature of the OCEBM is that the levels cover the entire range of clinical questions, such as the evidence for incidence, prognosis, therapeutic effects, rare harms, common harms, and usefulness of screening. To ease the reception of the hierarchy of evidence, we present in the results section uncontrolled studies, followed by cohort studies, case-control studies, and RCTs, for each drug and subgroup of the identified population. Two authors (MS, AG) independently extracted all data according to a preestablished data extraction form.

### Statistical analysis

To conduct a meta-analysis of incidence, we employed a logit transformation to stabilize the variance. We selected a random-effects model to account for anticipated heterogeneity among the included studies, for each subgroup of populations or measure of exposure to the primary outcome [33]. To measure heterogeneity, we utilized the I^2^ statistic and the Q test [34]. The I^2^ values were categorized as indicating low, moderate, substantial, and considerable heterogeneity, representing <25%, <25–50%, <50–75%, and ≥75%, respectively. The analyses were performed using R software, specifically version 4.3.1, with the metafor package [35]. We opted for the logit transformation of proportions as it is more suitable for a wide range of incidence values and when proportions are close to 0 or 1 [36].

## Results

### Search results

**Fig. 1:**
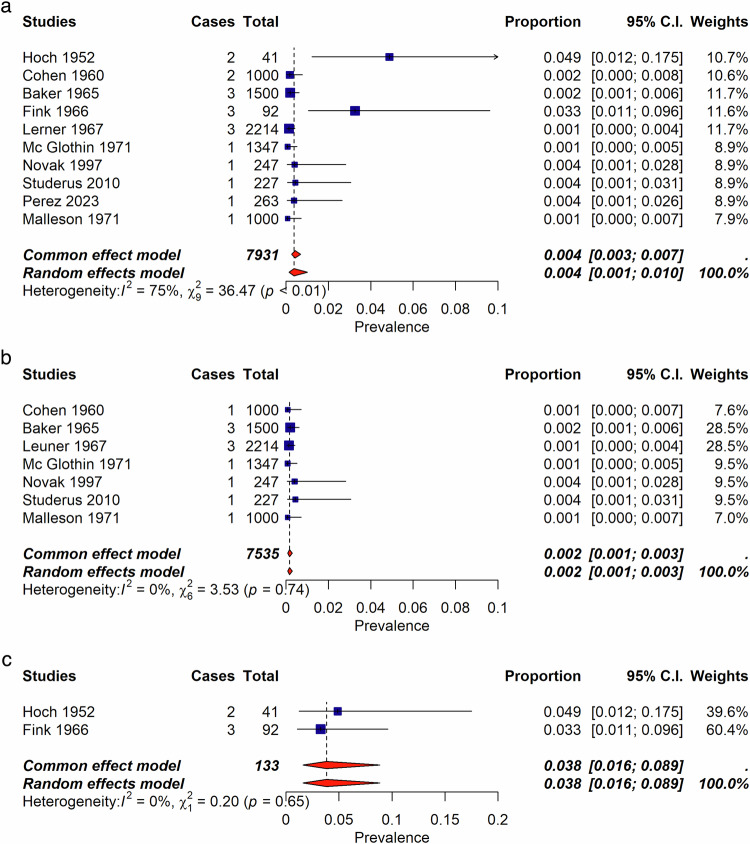
Incidence of psychedelic-induced psychosis according to the number of sessions. **a** Incidence of psychedelic-induced psychosis according to the number of sessions across healthy individuals, patients with depression, and patients with schizophrenia. Of importance, RCTs, UCT and cohort studies are combined in this forest plot. **b** Incidence of psychedelic-induced psychosis according to the number of sessions, in healthy individuals only. Please note that Studerus et al. [75], Novak et al. [79] studies are RCTs. All other studies are retrospective cohort studies. When excluding these 2 RCTS, the incidence does not change. When considering only those 2 RCTS, the incidence is 0.4% (95%; CI 0.1–1.7)(I^2^ = 0%). **c** Lifetime occurrence of prolonged psychosis according to number of sessions considering only studies that included patients with schizophrenia.

Among the 2170 records identified, we assessed 223 full-text records for eligibility and included 131 publications published from 1947 to 2023, encompassing 14 systematic reviews, 20 non-systematic reviews, and 96 individual studies. These studies were 35 RCTs, 10 case-controls studies, 30 uncontrolled trials (UCTs), and 22 cohort studies (retrospective or prospective cohorts) (Supplementary Table S1 and Fig. 1). Table 1 reflects the characteristics of included studies, and Table 2 of the reviews. The list of the 91 excluded studies is reported in supplementary Table S2.

**Table 1:** Clinical studies reporting potential psychedelic-induced psychosis.

Clinical studies							
Author, year	Title	Drug	Type of study	Setting Mean age, %male	Main findings	RoB 2 or ROBINS-I/COIs	LoE OCEBM Levels of Evidence^a^
**1. LSD**							
**1.1. UCT and cohort studies**							
**UCT**							
**Stoll 1947** [42]	Lysergsäure-diäthylamid, ein Phantastikum aus der Mutterkorngruppe	LSD	UCT	16 healthy volunteers and 6 patients with treatment-resistant schizophrenia received 30 μg and 130 μg dose of LSD respectively	*Patients with schizophrenia were medication-free. They experienced fewer visual hallucinations and less euphoria compared to healthy individuals, although the drug’s effects lasted longer. *None of the patients showed any worsening of their symptoms.	CR; n.m.	Level 2
**Condrau 1949** [43]	Klinische Erfahrungen an Geisteskranken mit Lysergsäure- Diäthylamide	LSD	UCT	Seven healthy volunteers and 30 patients with schizophrenia received up to 280 μg of LSD	*Patients with schizophrenia showed greater resistance to the effects of LSD compared to healthy volunteers, experiencing fewer hallucinations, less euphoria, and fewer side effects such as nausea and headaches. *None of the patients experienced a worsening of their symptoms.	CR; n.m.	Level 2
**Busch & Johnson 1950** [103]	L.S.D. 25 As an Aid in Psychotherapy	LSD	UCT	18 patients with schizophrenia, and 3 patients with bipolar disorders received 30 to 40 μg LSD	*All patients exhibited increased psychomotor activity, and most experienced euphoric states. Four patients with paranoid schizophrenia required hydrotherapy. *Patients with bipolar disorder were generally unstable and irritable, also requiring hydrotherapy for calming. *Eight patients with schizophrenia and psychoneurosis showed improvement with the combination of LSD and psychotherapy. Of these, two experienced relief from childhood traumatic episodes. No long-lasting symptoms were observed.	CR; n.m.	Level 2
**De Giacomo 1951** [104]	Catatonie toxique expérimentale	LSD	UCT	12 patients with schizophrenia received from 30 to 500 μg of LSD	*The author observed the induction of a catatonic state in 5 patients with schizophrenia. This effect, specific to patients with schizophrenia, has not been reported in most subsequent studies. *No long-lasting symptoms were observed.	CR; n.m.	Level 2
**Mayer-Gross 1951** [105]	Experimental Psychoses And Other Mental Abnormalities Produced By Drugs	LSD	UCT	13 patients with schizophrenia received from 30 to 500 μg of LSD	*Half of the patients with schizophrenia developed euphoria during the LSD session. *One patient experienced an outburst of anger during the session. *No long-lasting symptoms were observed.	CR; n.m.	Level 2
**Forrer & Goldner 1951** [46]	Experimental physiological studies with lysergic acid diethylamide (LSD-25)	LSD	UCT	Six patients with resistant schizophrenia received 0.5 to 6 μg/per/kg μg IM LSD	*Five of six patients presented euphoric states and hallucinations during the 42 LSD sessions. A dose-response occurrence of euphoric states is described *Three patients presented increased sexual material *These patients presented fewer side effects (nausea, vomiting) and no long-lasting symptoms	CR; n.m.	Level 2
**Belsanti 1952** [44]	Modificazioni neuro-psico-biochimiche indotte dalla dietilamide dell’acido lisergico in schizofrenici e frenastenici	LSD	UCT	14 patients with schizophrenia received from 80 to 480 μg of LSD	*Patients with schizophrenia seem more resistant to LSD effect that healthy individuals *LSD accentuate previous symptomatology in patients with schizophrenia *No long-lasting symptoms were found	CR; n.m.	Level 2
**Liddell & Weil-Malherbe 1953** [47]	The effects of methedrine and Lysergic acid diethylamide on mental processes and on tile blood adrenaline level	LSD	UCT	Ten patients with schizophrenia received 60 μg IV LSD	*Six patients presented during the LSD session rapid mood swings and disorganization *Two patients were frightened and inaccessible *No long-lasting symptoms were found after the LSD session	CR; n.m.	Level 2
**Katzenelbogen & Fang 1953** [106]	Narcosynthesis effects of sodium amytal, methedrine and L.S.D-25	LSD	UCT	20 patients with schizophrenia received from 10 to 50 μg of oral LSD	*All patients experimented mood swings, with mainly euphoric states, except one patient that presented negativistic features *The authors described specific minimal effective doses for each patient	CR; n.m.	Level 2
**Cholden et al. 1955** [107]	Clinical reactions and tolerance to LSD in chronic schizophrenia	LSD	UCT	20 patients with schizophrenia received dose from 100 to 500 μg of IM LSD	*Patients with schizophrenia exhibited an increased expression of their emotions *Patients with catatonic or paranoid features showed significant improvement, with enhanced communication *Some patients with acute schizophrenia may experience an ‘intensification’ of psychomotor agitation; however, this phenomenon is limited to the LSD session *Patients were exposed from 100 to 500 μg within 5 days duration. Tolerance to LSD was obtained in 2–3 days of drug administration. A period of 4–6 days free of LSD is necessary to reinstate the original reaction to LSD. No cross-tolerance between LSD and mescaline was found	MR; n.m.	Level 2
**Bercel et al. 1956** [38]	Model Psychoses Induced by LSD-25 in Normals I. Psychophysiological Investigations, with Special Reference to the Mechanism of the Paranoid Reaction	LSD	UCT	25 healthy individuals received oral LSD from 30 to 100 μg of LSD. 20-35(68%)	*Two individuals experienced delusions of persecution during the LSD session *One individual exhibited hypomania during the LSD session *Ego dissolution and depersonalization persisted in one individual for more than 24 h	MR; n.m.	Level 3
**Abramson 1960** [108]	The Use of LSD in Psychotherapy	LSD	UCT	Six patients with treatment-resistant severe autism received LSD at 40 μg. 5-14(n.a.)	* Patients received weekly LSD treatment, averaging 3 to 6 sessions. *Five out of six patients showed improvement with no side effects.	MR; n.m.	Level 2
**Cohen 1960** [37]	Lysergic Acid Diethylmaide: side effects and complications	LSD	UCT	A total of 62 physicians who had experience with prescription of LSD or mescaline were asked on psychiatric side effects of treatments. n.a	*Dosages of LSD ranged from 25-1500 mcg. *A prevalence of 1.8/1000 of prolonged psychotic reaction ( > 48 h) was reported	MR; n.m.	Level 2
**Anastasopoulos & Photiades 1962** [45]	Effects of LSD-25 on relatives of schizophrenic patients.	LSD	UCT	19 parents of patients with schizophrenia received LSD from 1 to 1.5 μg/kilo over 97 sessions. n.a	*Patients with schizophrenia require higher doses than their parents and siblings to exhibit symptoms. However, paranoid features were more likely to occur in parents and siblings, sometimes resulting in long-lasting psychotic symptoms and persistent insomnia (lasting from a few days to 6 weeks). *The authors suggest that reactions to LSD might predict the risk of developing schizophrenia.	CR; n.m.	Level 2
**Freedman et al.1962** [109]	Autistic schizophrenic children: An experiment in the use of d-lyergic acid diethylamide (LSD-25)	LSD	UCT	12 patients with treatment-resistant severe autism received oral 50 to 200 μg. LSD 5-12(75%)	*Most patients were on antipsychotic medications *The authors observed rapid mood swings in all participants, with only one experiencing panic. Generally, there was an increase in communication and motor activity	CR; n.m.	Level 2
**Fischer & Castile 1963** [110]	Interim report on research project: An investigation to determine therapeutic effectiveness of LSD-25 and Psilocybin on hospitalized severely emotionally disturbed children.	LSD	UCT	Patients with severe autism and psychotic features received LSD from 50 to 400 μg, or psilocybin 250 to 300 mcg. 5-13(n.a.)	* The authors found that the best candidates for LSD treatment were patients who could communicate verbally and exhibited more psychotic features than severe autistic traits	MR; n.m.	Level 2
**Bender et al. 1962** [111]	Treatment of autistic schizophrenic children with LSD-25 and UML-491	LSD	UCT	44 patients with a diagnosis of autism and psychotic features received LSD or UML491. 50 to 150 μg of LSD. 6-15(n.a.)	*The authors observed short-term improvements in speech and behavior, greater emotional responsiveness, increased positive mood, and a decrease in compulsive ritualistic behavior *The authors found a rapid reduction in secondary negative symptoms	CR; n.m.	Level 2
**Bender & Faretra 1963** [48]	LSD and UML treatment of hospitalized disturbed child	LSD	UCT	50 prepubert children with a diagnosis of schizophrenia were included. 6 patients received LSD up to 150 μg. n.a. (76%) daily	*Patients received daily LSD for 2 to 12 months *Patients showed increased responsiveness to environmental stimuli *While some aggressive children became remarkably quieter after LSD treatment, some quieter children became mildly aggressive *No serious adverse events were reported	CR; n.m.	Level 2
**Bender 1966** [112]	D-lysergic Acid in the Treatment of the Biological Features of Childhood Schizophrenia	LSD	UCT	12 prepubert children with a diagnosis of schizophrenia were included. 6 patients received LSD up to 150 μg. n.a. (100%) daily	*The authors found that patients who received LSD exhibited changes in neurological signs, memory function, visual-motor skills, and body image *Children with psychotic symptoms showed improvements in overall behavior and attitudes, better reality contact, and enhanced thinking *The authors did not observe any side effects or acute psychotic reactions as reported in adults	CR; n.m.	Level 2
**Fink 1966** [13]	Prolonged adverse reactions to LSD in psychotic subjects.	LSD	NRCT	One or multiple IV LSD session (0.5 µg/kg to 10 µg/Kg) -or placebo- to 65 patients with schizophrenia. 36-year-old(n.a.)	*Patients received either LSD or a placebo medication *The authors found a 3.2% (3 out of 92) incidence of long-lasting psychotic reactions, described as exacerbations of preexisting psychopathology accompanied by signs of confusional delirium	CR; pf, npo	Level 2
**Blumfield & Glickman 1967** [113]	Ten months’ experience with LSD users admitted to a country psychiatric receiving hospital	LSD	UCT	23 patients hospitalized for LSD induced psychosis.	*80% of patients were diagnosed with schizophrenia or borderline schizophrenia *The authors suggest that preexisting sociopathic characteristics, along with a genetic predisposition for substance abuse, may have contributed to the involvement with LSD, possibly as a means of escaping other pressures or as an attempt at self-medication	CR; n.m.	Level 3
**Bowers et al. 1990** [12]	Psychotogenic Drug Use and Neuroleptic Response	LSD	UCT	Treatment of 32 case of acute psychosis in emergency service that were prior users of LSD. 21.2(73%).	*Haloperidol and perphenazine were not effective in treating LSD-induced psychosis *Male participants with psychosis and a history of using psychotogenic drugs had a poorer response to neuroleptics compared to those without such a history	CR; p.f., n.p. o.	Level 3
**Cohort studies**							
**Malleson 1971** [78]	Acute Adverse Reactions to LSD in Clinical and Experimental Use in the United Kingdom	LSD	Retrospective cohort	Report by clinicians of their practice with LSD. (n.a)	* Out of 4,300 individuals who received one or multiple sessions of LSD, 37 experienced psychedelic-induced psychosis. Of these 37 patients, 10 developed a persistent and chronic psychotic state, while 19 recovered completely. Among those who recovered, 7 did so over a period of 3 months, 3 recovered between 2 weeks to 3 months, and 9 within 2 weeks.	CR; n.m.	Level 2
**McGlothlin & Arnold 1971** [51]	LSD revisited. A ten-year follow-up of medical LSD use	LSD	10-year follow-up study	247 persons who received LSD in nonmedical or psychotherapeutic setting. 44(66%)	* 58% of subjects reported some lasting effects, such as reduced anxiety to stress, 6 months after three sessions of 200 μg LSD. There is little evidence of measurable, lasting personality or behavior changes * 36 of the 247 participants, and only 4 of the 32 participants with serious psychiatric disorders, experienced ‘LSD-like recurrences’ that can be classified as minor psychotic or psychosomatic symptoms *One case of prolonged psychosis, requiring a one-week hospitalization, was reported among the 247 LSD users. This occurred after the individual’s third LSD experience (1/276 sessions)	CR; p.f.	Level 2
**Dewhurst & Hatrick 1972** [114]	Differential diagnosis and treatment of lysergic acid diethylamide induced psychosis	LSD	Retrospective cohort	19 patients presenting LSD-induced psychosis	*The authors identified distinguishing features of LSD-induced psychosis, including a loss of time sense, grandiose delusions of a pseudo-philosophical nature, visual hallucinations, perceptual disturbances, and a common regression to childhood	CR; n.m.	Level 3
**Roy 1981** [115]	LSD and onset of schizophrenia.	LSD	Retrospective cohort	74 patients were divided in two groups of chronic schizophrenia with prior use of LSD and psychedelic naïve group. 20.35(78%).	*The authors found no significant difference between 37 patients with chronic schizophrenia who were LSD users prior to their diagnoses and control patients with chronic schizophrenia who were psychedelics-naïve *The mean age of onset of schizophrenia was lower in the LSD users group	CR; n.m.	Level 2
**Vardy & Kay 1983** [52]	LSD psychosis or LSD-induced schizophrenia?	LSD	Prospective cohort	Prospective follow-up at 3–5 years of 29 patients with LSD**-psychosis** and 29 FEP. 20.46(n.a)	*The frequency of LSD intake was most commonly 1–5 times (34.6% of cases), occasionally 6-10 times (7.7%), but ranged up to about 100 times (2 subjects) *Patients with LSD-induced psychosis exhibited psychotic symptoms after LSD ingestion that persisted for at least 2 weeks *The authors found that the rate of parental alcoholism in LSD psychosis patients exceeded that in schizophrenic patients *The findings support a model of LSD psychosis as a drug-induced schizophreniform reaction in individuals vulnerable to both substance abuse and psychosis	MR; n.m.	Level 2
**Niemi-Pynttäri et al. 2013** [50]	Substance-induced psychoses converting into schizophrenia: a register-based study of 18,478 Finnish inpatient cases	LSD	Register-based study	Retrospective analysis of the nationwide Finnish hospital discharge register with follow-up of patients (1987-2003). Total of 18.478 patients, with 84 HIP. 24.3(7)	*Eight-year cumulative risk to receive a schizophrenia spectrum disorder diagnosis reported: 46%(95%CI, 35-57%) for cannabis-induced psychosis 30%(95%CI, 14-46%) for amphetamine-induced psychosis 24%(95%CI, n.a.) for hallucinogens-induced psychosis (n = 15/84 ; N = 3) *HR (95%CI): 2.69 (1.59-4.55) for hallucinogens. PCP accounted for 1/3 of studies *Total of 18.478 patients, with 84 patients presenting a hallucinogen-induces psychosis. Of those patients 15(17.8%) converged to a diagnosis of schizophrenia	SR; p.f.	Level 2
**1.2. Case-control studies and RCT**							
**CC**							
**Sloane & Doust 1954** [63]	Psychophysiological Investigations in Experimental Psychoses: Results of the Exhibition of D-Lysergic Acid Diethylamide to Psychiatric Patients	LSD	Case-control study	Four inpatients with schizophrenia, 2 with schizoaffective disorders, 12 with depression were compared to 11 healthy controls and received 40 to 120 μg of LSD. 41 ± 8.25(n.a)	*Patients with schizophrenia exhibited an increased mood during the LSD session *A few individuals, both patients and controls, showed increased suspiciousness	CR; n.m.	Level 2
**Smart et al. 1966** [116]	A Controlled Study of Lysergide in the Treatment of Alcoholism.The Effects on Drinking Behavior	LSD	Case-control study	30 individuals with alcohol use disorders and received LSD 800 µg, ephedrine 60 mg or placebo. 40 ± 6(93%).	*All three groups showed improvement at the 6-month follow-up, with no significant differences between the groups. The gain in abstinence time for the LSD, ephedrine, and control groups was 34%, 32%, and 20%, respectively *No psychosis was reported among participants *Results from Baker et al. [76] and Leuner [77] are included in this paper (Supplementary Table S8)	CR; n.m.	Level 3
**Ungerleider 1968** [60]	The ‘bad trip’ – The etiology of the adverse LSD reaction	LSD	Case-control study	25 inpatients hospitalized for LSD-psychosis were compared to 25 regular users of LSD (religious group). n.a.(n.a.).	*LSD may cause adverse effects in individuals with schizoid personalities *40% of patients admitted to the hospital had been diagnosed as ‘psychotic’, and 37% had received psychiatric care previously	MR; n.m.	Level 3
**Langs & Barr 1968** [84]	Lysergic acid diethylamide (LSD-25) and schizophrenic reactions. A comparative study.	LSD	Case-control study	30 healthy individuals received LSD (100 μg). 20 controls received placebo and were compared to 20 hospitalized patients with schizophrenia. 30(100%)	*LSD primarily causes visual hallucinations, while schizophrenia is characterized by auditory hallucinations *Both groups experienced delusions, body image changes, and altered self-perception, but delusional thinking was more prominent in schizophrenia	CR; n.m.	Level 1
**Hays & Tilley 1973** [61]	The differences between LSD psychosis and schizophrenia.	LSD	Case-control study	Authors compared 10 LSD-psychosis to 114 controls (patients with schizophrenia). n.a.	*LSD-induced psychosis is clinically distinct from schizophrenia *Patients with LSD psychosis had fewer auditory hallucinations, more visual hallucinations and illusions, fewer primary delusions, and less mood flattening	C.R.; n.m.	Level 3
**Lev-Ran et al. 2014** [117]	Clinical characteristics of individuals suffering from schizophrenia and Hallucinogen Persisting Perceptual Disorders: A preliminary investigation	LSD	Case-control study	Case-control study gathering hospitalized patients that had prior LSD use. 32.3 ± 7.6(84.6%)	*Little is known about the co-occurrence of schizophrenia and HPPD or its clinical implications. The authors describe 12 cases of schizophrenic patients with perceptual distortions *No significant differences were found between the groups in response to antipsychotics or side effects	SR; no coi	Level 3
**Lev-Ran et al. 2015** [118]	Schizophrenia and hallucinogen persisting perception disorder: A clinical investigation	LSD	Case-control study	Case-control study with 80 patients: 37 patients with schizophrenia and HPPD, compared 43 patients with schizophrenia. 31.5(90%)	*Schizophrenia patients with HPPD exhibited less severe negative symptoms and lower general psychopathology scores, despite persistent perceptual disturbances *There were differences in treatment responses between the groups	MR; no coi	Level 3
**RCT**							
**Shirvaikar & Kelkar 1966** [73]	Therapeutic trial of lysergic Acid diethylamide (LSD) and thioridazine in chronic schizophrenia	LSD and Thioridazine	Experimentaldouble-blind, controlled	Ten patients with a diagnosis of chronic schizophrenia received LSD during 16 sessions with 50 to 150 μg LSD combined with 50 to 100 mg of thioridazine	* Patients with chronic schizophrenia and predominant negative symptoms showed improvement with LSD, experiencing euphoric reactions in the first sessions and increased social behavior in the second week	MR; n.m	Level 2
**Tomsovic & Edwards 1970** [119]	Lysergide treatment of schizophrenic and nonschizophrenic alcoholics: a controlled evaluation	LSD	RCT	75 individuals with alcohol use disorders of whom 23 also had the diagnosis of schizophrenia received one session of 500 μg of LSD. 43(100%)	* Non-schizophrenic individuals responded better to the drug, with more abstinent at a 1-year follow-up compared to schizophrenics and alcoholics treated by other methods * There was significant uncertainty in schizophrenia diagnoses (63% of inclusions) * Two non-schizophrenic patients lost touch with reality during the peak of their experience and behaved bizarrely	CR; n.m.	Level 3
**Gasser et al. 2014** [120]	Safety and efficacy of lysergic acid diethylamide-assisted psychotherapy for anxiety associated with life-threatening diseases	LSD	RCT	12 patients with anxiety associated with life-threatening diseases received LSD or active placebo (20 to 200 μg)	* Psychological side effects were mild and limited; no prolonged anxiety, psychotic episodes, or flashbacks were reported	M.R.; n.p.o.	Level 1
**Schmid et al. 2015** [121]	Acute Effects of Lysergic Acid Diethylamide in Healthy Subjects	LSD	Cross-over RCT	LSD (200 μg) and placebo were administered to 16 healthy subjects. 28.6 ± 6.2(50%)	* LSD reduced associative connectivity but increased sensory-somatomotor and thalamic connectivity across the brain * LSD’s neuropharmacological effects involve the 5-HT2A receptor	LR; p.f.	Level 2
**Preller et al. 2018** [65]	Changes in global and thalamic brain connectivity in LSD-induced altered states of consciousness are attributable to the 5-HT2A receptor	LSD	Cross-over RCT	LSD (100 μg), ketanserin and placebo were administered to 24 healthy subjects. 25 ± 3.6(79.5%)	* Ketanserin fully blocked both subjective and neural effects of LSD *Implication of the 5-HT2A receptor in LSD’s neuropharmacology	LR; p.f., n.p.o.	Level 1
**Wießner et al. 2021** [85]	LSD, madness and healing: Mystical experiences as possible link between psychosis model and therapy model	LSD	Cross-over RCT	24 healthy volunteers received 50 μg of LSD or inactive placebo. 35 ± 11(67%)	* Mystical experiences may bridge the psychosis model and therapeutic applications * LSD significantly heightened sensory sharpening, emotionality, cognition, and aberrant salience, potentially inducing psychotic-like experiences * Moderate negative correlations were found between psychedelic experiences and mindfulness, while suggestibility was not correlated	LR; p.f.,n.p.o.	Level 1
**2. DMT and Ayahuasca**							
**2.1. UCT and cohort studies**							
**UCT**							
**Gillin et al. 1976** [39]	The psychedelic model of schizophrenia: The case of N,N-dimethyltryptamine	DMT	UCT	Seven mg/kg of DMT intramuscularly to 15 male volunteers. Each subject was an experienced user of LSD or mescaline. n.a. (100%)	* Psychological changes appeared within 5 minutes of injection, peaked at 10-15 minutes, and lasted 45-120 minutes * Only one subject experienced elementary hallucinations, while two had paranoid symptoms lasting less than an hour *Review of the DMT model of schizophrenia	MR; n.m.	Level 3
**Jimenez-Garrido et al. 2020** [122]	Effects of ayahuasca on mental health and quality of life in naïve users: A longitudinal and cross-sectional study combination	Ayahuasca	UCT	Authors compare ayahuasca in ayahuasca-naïve subjects and ayahuasca experienced subjects (N = 40, N = 23). 35 ± 11.5(30%)	* DMT was associated with immediate reductions in depression, anxiety, and hostility, with the depression reduction persisting at 6 months * No adverse events were reported by the authors	MR; no coi	Level 3
**Cohort studies**							
**Lima & Toffoli 2011** [53]	An epidemiological surveillance system by the UDV: mental health recommendations concerning the religious use of hoasca	Ayahuasca	Epidemiological survey	Authors report observations obtained through a surveillance program from 1994 up to 2007. 130000 participants	* Authors report 20 psychotic-like phenomena cases, with only 4 having a clear temporal link to ingestion and no prior psychiatric history; 5 cases showed onset or worsening in patients with pre-existing psychiatric conditions * Authors estimate 1 psychotic event per 50,000 ingestions	CR; n.m.	Level 2
**2.2. CC and RCT**							
**Case-control**							
**Bickel et al. (article in german) 1977** [123]	Effects of N,N-dimethyltryptamine (DMT) on tests of psychoticism	DMT	Case-control study	26 healthy individuals (DMT, 250 µg/kg), compared to 12 controls 24.7 ± 3.6(60%).	*Study of the effect of DMT on psychoticism. 7 out of 19 variables were affected, most of which are considered to be linked to attention	CR; n.m.	Level 3
**Bouso et al. 2015** [124]	Long-term use of psychedelic drugs is associated with differences in brain structure and personality in humans	Ayahuasca	Case-control study	22 case (regular users of ayahuasca, >50 use in the last 2 years) and 22 controls 41.3 ± 12.3(62.5%)	*Ayahuasca users averaged 123 ± 75.3 sessions over 5.3 ± 2.75 years *Regular psychedelic use may lead to structural changes in brain regions (PCC cortical thickness) involved in attention, self-referential thought, and internal processing *No increase in psychopathology or decline in psychological performance between groups; both showed similar levels of positive symptoms	MR; no coi	Level 2
**RCT**							
**Strassman & Qualls 1994** [125]	Dose-Response Study of N,N-Dimethyltryptamine in Humans.	DMT	RCT	IV-DMT administered at 0.05, 0.1, 0.2, and 0.4 mg/kg to 12 experienced hallucinogen users. 41.5 ± 1.5(91.1%)	*Authors found that threshold doses for significant effects relative to placebo were also hallucinogenic (0.2 mg/kg and higher)	MR; pf, npo	Level 2
**Dos Santos et al. 2021** [126]	Ayahuasca Improves Self-perception of Speech Performance in Subjects With Social Anxiety Disorder: A Pilot, Proof-of-Concept, Randomized, Placebo-Controlled Trial	Ayahuasca	RCT	17 participants with social anxiety disorders received ayahuasca 2 ml/kg or placebo during one session. 41.5 ± 1.5(91.1%)	*Ayahuasca was generally well tolerated, causing mainly nausea, gastrointestinal discomfort, and vomiting. One participant experienced distress, two were confused, and one had a fear reaction	MR; p.f.; n.p.o.	Level 1
**3. Psilocybin and mescaline**							
**3.1. UCT and cohort studies**							
**UCT**							
**Rümmele and Gnirss 1961** [40]	Untersuchungen mit Psilocybin, einer psychotropen Substanz aus Psilocybe Mexicana	Psilocybin	UCT	In this observational study, healthy volunteers (N = 18; N = 9) received oral administration (6 mg sublingual or 10 mg) of psilocybe Mexicana. n.a.	*At 6 mg, no psychotic symptoms were reported during or after administration *At 10 mg, participants showed strong physical reactions and psychotic-like symptoms (depersonalization, anxiety from hallucinations/delusions), but no lasting psychosis	CR; n.m.	Level 2
**Vollenweider et al. 1997** [127]	Positron emission tomography and fluorodeoxyglucose studies of metabolic hyperfrontality and psychopathology in the psilocybin model of psychosis.	Psilocybin	UCT	15 healthy volunteers with PET and FDG prior to and following psilocybin (15-20 mg) and ketamine, ketamine and d-amphetamine, psilocybin and d-amphetamine. 33.3 ± 4.8(53%)	*Psilocybin-induced psychotic symptoms appeared 20-30 minutes after ingestion, peaking within 60 minutes and lasting 1–2 h *Excessive 5-HT2 receptor activation causes a hyperfrontal metabolic pattern, similar to acute psychotic episodes in schizophrenia, contrasting with hypofrontality seen in chronic cases *Serotonergic agonists like psilocybin and LSD can mimic key aspects of schizophrenic psychosis	MR; p.f.	Level 2
**Cohort studies**							
**Bergman 1971** [54]	Navajo peyote use: Its apparent safety	Mescaline (Peyote)	Population survey. Description of 3 cases reports	Authors report a 4-year community psychiatric service mental among Navajo Indians that are peyote users. 30000 participants.	*Psychotic episodes in the Navajo Native American Church are estimated at 1/70,000 peyote ingestions *Combining alcohol with peyote can trigger brief paranoid episodes *Schizophrenia patients attending many ceremonial sessions reported no significant adverse events	CR; n.m.	Level 2
**Garcia-Romeu et al. 2015** [128]	Psilocybin-occasioned Mystical Experiences in the Treatment of Tobacco Addiction	Psilocybin	Open-label study	15 smokers received 2 or 3 doses of psilocybin in the context of cognitive behavioral therapy (CBT) for smoking cessation. A follow-up period was conducted	*60% of participants (9 of 15) met the criteria for a “complete” mystical experience *Smoking cessation was strongly linked to mystical experiences during psilocybin sessions and retrospective ratings of personal and spiritual significance *Only one participant had a bad trip, which ended with the psilocybin session	MR; p.f.; n.p.o.	Level 2
**Anderson et al. 2020** [41]	Psilocybin-assisted group therapy for demoralized older long-term AIDS survivor men: An open-label safety and feasibility pilot study	Psilocybin	Open-label study	0.3–0.36 mg/kg psilocybin was administered to 18 depressed patients with AIDS 59.2(100%)	*Patients presented various underlying psychiatric disorders (GAD, borderline personality disorders) *Mild paranoia/ideas of reference were found in 4/18 patients (22%) *Important anxiety was found in 8/18(44%) patients	SR; p.f., n.p.o.	Level 2
**Aaronson et al. 2023** [6]	Single-Dose Synthetic Psilocybin With Psychotherapy for Treatment-Resistant Bipolar Type II Major Depressive Episodes A Nonrandomized Controlled Trial	Psilocybin	Open-label study	Patients with bipolar type II disorders received a single dose of psilocybin-assisted psychotherapy (25 mg). 37.8 ± 11.6(40%)	*Exclusion criteria included history of BDI disorder, schizophrenia, psychosis (including substance-induced or due to a medical disorder), delusions, paranoid, schizoaffective, or borderline personality disorder, or any substance use disorder within the past 12 months *Authors found no side-effects at the given dose for selected patients. The authors conclude that it is premature to extrapolate these data to the BDI population, who are at higher risk of mania and psychosis	LR; p.f.; n.p.o.	Level 2
**3.2. RCT**							
**Vollenweider et al. 1999** [9]	5-HT Modulation of Dopamine Release in Basal Ganglia in Psilocybin-Induced Psychosis in Man—A PET Study with [11 C]raclopride	Psilocybin	RCT	Seven healthy individuals received placebo or psilocybin (0.25 mg/kg p.o.). 27 ± 2.3(100%)	*Psilocybin can produce a few hours psychotic syndrome that resembles in certain respects acute schizophrenic decompensation *Psilocybin-induced psychotic symptoms may be mediated preferentially by serotonergic rather than mesostriatal dopaminergic systems *Psilocybin decreased [11 C]raclopride binding in the caudate nucleus and putamen, reflecting an increase in striatal dopamine concentration. Authors question whether psychosis-like symptoms are linked to increased striatal dopamine activity	MR; p.f.	Level 1
**Hasler et al. 2004** [72]	Acute psychological and physiological effects of psilocybin in healthy humans: a double-blind, placebo-controlled dose-effect study	Psilocybin	RCT	Eight healthy individuals received placebo, and 45, 115, 215, and 315 ug/kg psilocybin. 29.5 ± 7.3(50%)	*Dose-dependent effects of psilocybin on psychopathological and physiological parameters (n = 8) *Only one subject reacted with transient anxiety to high dose (315 μg/kg) of psilocybin as a consequence of loosening of ego-boundaries	MR; p.f.	Level 1
**Grob et al. 2011** [129]	Pilot Study of Psilocybin Treatment for Anxiety in Patients With Advanced-Stage Cancer	Psilocybin	RCT	RCTs of patients with advanced-stage cancer and anxiety (N = 12; 0.2 mg/kg)	*There were no clinically significant adverse events with psilocybin *All subjects tolerated the treatment sessions well, with no indication of severe anxiety or a “bad trip.” Indeed, the current dose of psilocybin produced only modest effects on the anxious ego dissolution scale of the 5D-ASC	MR; p.f., n.p.o.	Level 1
**Griffiths et al. 2016** [130]	Psilocybin produces substantial and sustained decreases in depression and anxiety in patients with life-threatening cancer: A randomized double-blind trial	Psilocybin	RCT	Five weeks crossover study with 6 months follow-up. Two doses delivered (N = 51; 1–3 mg placebo dose and 22-30 mg/70 kg). n.a. (91.7%)	*Adverse events during the psychedelic experience reported were an episode of anxiety that occurred in 26% of participants in the high dose session and 15% in the low-dose session. One participant had a transient episode of paranoid ideation (2% of high-dose sessions) *There were no cases of prolonged psychosis	MR; p.f.	Level 1
**Ross et al. 2016** [131]	Rapid and sustained symptom reduction following psilocybin treatment for anxiety and depression in patients with life-threatening cancer: a RCT	Psilocybin	RCT	Seven weeks RCT with a crossover design, and follow-up at 33 weeks. Unique dose of psilocybin (N = 31; 21 mg/ 70 kg). 56.3 ± 7.3(62%)	*The most common psychiatric adverse event was transient anxiety (17%) and transient psychotic-like symptoms (7%: one case of transient paranoid ideation and one case of transient thought disorder)	LR; p.f., n.p.o.	Level 1
**Davis et al. 2021** [71]	Effects of Psilocybin-Assisted Therapy on Major Depressive Disorder A RCT	Psilocybin	RCT	Eight weeks RCT with 1 month follow up. Two doses of psilocybin delivered (N = 16; 20 and 30 mg/70 kg).7.4 ± 12(67%)	*Adverse events reported during the psychedelic experience were: ‘I felt as if I was dead or dying 12 (25%)’ ‘I had the feeling that people were plotting against me 4 (8%)’(2/16) ‘Paranoia (3%)’(1/16)	LR; p.f., n.p.o.	Level 1
**Carhart-Harris et al. 2021** [70]	Trial of Psilocybin versus Escitalopram for Depression	Psilocybin	RCT	Six weeks RCT, comparing psilocybin with escitalopram. 2 doses of psilocybin delivered (N = 20; 25 and 1 mg/70 kg). 41.3 ± 10.3(35%)	*Adverse events after the psychedelic experience: One case of abnormal dreams of illusion and of insomnia	LR; p.f., n.p.o.	Level 1
**Goodwin et al. 2022** [2]	Single-Dose Psilocybin for a Treatment-Resistant Episode of Major Depression	Psilocybin	RCT	Three weeks phase 2, dose-finding RCT, with 12 weeks follow up. Unique dose of psilocybin (N = 233;1, 10 or 25 mg/70 kg) 40.1 ± 12(53%)	*Adverse events during the psychedelic experience: Group 25 mg – two suicidal ideations, and two panic attacks Group 10 mg- one suicidal ideation, and one abnormal dream	LR; p.i.	Level 1
**Bogenschutz et al. 2022** [132]	Percentage of Heavy Drinking Days Following Psilocybin-Assisted Psychotherapy vs Placebo in the Treatment of Adult Patients With Alcohol Use Disorder A Randomized Clinical Trial	Psilocybin	RCT	Participants with alcohol use disorders were given at week 4 and 8 psilocybin or diphenhydramine during 2 day-long sessions. 46 ± 12(55.8%)	*Two participants given psilocybin took 10 mg of diazepam orally for anxiety during their second session. The anxiety resolved within 45 minutes for one and 210 minutes for the other *One participant on psilocybin experienced passive suicidal thoughts for 15 minutes during a session, which resolved without further issues *No lasting disturbances were observed, such as psychosis or hallucinogen persisting perception disorder	MR; p.f., n.p.o.	Level 1
**Von Rotz et al. 2023** [133]	Single-dose psilocybin-assisted therapy in major depressive disorder: A placebo-controlled, double-blind, RCT	Psilocybin	RCT	Three weeks RCT. Unique dose of psilocybin (N = 52; 15 mg/70 kg). 36.7 ± 10.4 (63.2%)	*No serious adverse events were recorded	LR; p.f., n.p.o.	Level 1
**4. MDMA/Ecstasy**							
**4.1. Cohort studies**							
**Landabaso et al. 2002** [56]	Ecstasy-induced psychotic disorder.	Ecstasy	Prospective cohort	Follow-up of 32 patients diagnosed with ecstasy-induced psychotic disorder (DSM-IV) in two drug-dependency outpatient centers. 22.47 ± 2.75(84%)	*6/52 patients had previously presented psychotic symptoms *All subjects received treatment with olanzapine and showed after 6-month a statistically significant clinical reduction (BPRS, HDRS and CGI) *The symptoms were most often positive, such as delusions (96%), hallucinations (96%), and conceptual disorganization (96%). Yet, negative symptoms were also detected, such as depressive mood (90%) and blunted affect (81%)	MR; p.f., no coi	Level 2
**4.2. CC and RCT**							
**Case-control**							
**Rugani et al. 2012** [62]	Symptomatological features of patients with and without Ecstasy use during their first psychotic episode.	Ecstasy	Case-control study	Authors compared psychotic patients with (n = 23) and without (n = 46) recent user of Ecstasy, during their first psychotic episode. 21 ± 6(100%)	*Patients with psychosis and with recent use of MDMA were characterized by less blunted affect and more hostile behavior *Psychosis with a high level of aggressiveness and violence constitutes an important ‘side-effect’ that surely runs counter to the expected entactogenic action of ecstasy	CR; no coi	Level 2
**RCT**							
**Bouso et al. 2008** [134]	MDMA-assisted psychotherapy using low doses in a small sample of women with chronic posttraumatic stress disorder	MDMA	RCT	29 subjects received MDMA-assisted psychotherapy with low doses of MDMA 50 and 75 mg. 35.6(0%)	*Neither of the two doses of MDMA increased symptomatology in any of the psychopathological scales in any of the subjects treated	HR; n.p.o	Level 2
**Mithoefer et al. 2011** [69]	The safety and efficacy of MDMA assisted psychotherapy in subjects with chronic, treatment-resistant posttraumatic stress disorder: the first randomized controlled pilot study	MDMA	RCT	20 patients with chronic and treatment-resistant PTSD received 125 mg and an optimal dose of 65 mg or a placebo. 40.4 ± 7.26(85%)	*60% of patients experimented anxiety *No psychotic symptoms emerged among included patients	CR; n.p.o.	Level 1
**Oehen et al. 2013** [135]	A randomized, controlled pilot study of MDMA-assisted psychotherapy for treatment of resistant, chronic Post-Traumatic Stress Disorder (PTSD)	MDMA	RCT	12 patients with PTSD were enrolled for low-dose (25 + 12 mg) or full-dose MDMA (125 + 62.5 mg) 41.2 ± 11.2(83%)	*Patients were proposed an overnight stay for safety reasons after each MDMA session *One patient presented an insomnia and benefit from a zolpidem treatment *No drug-related serious adverse events occurred	LR; n.p.o., no coi	Level 1
**Mithoefer et al. 2019** [90]	MDMA-assisted psychotherapy for post-traumatic stress disorder in military veterans, firefighters, and police officers: a randomised, double-blind, dose-response, phase 2 clinical trial	MDMA	RCT	26 veterans were assigned to received 30 mg (n = 7), 75 mg (n = 7), or 125 mg (n = 12) of MDMA plus psychotherapy. 37.2 ± 10.3(73%)	*Mild to severe anxiety was reported by participants. A dose response relation for occurrence of anxiety is found, with anxiety increasing for each dose (92% at 125 mg) *No drug-related serious adverse events occurred	LR; n.p.o.	Level 1
**Danforth et al. 2018** [136]	Reduction in social anxiety after MDMA-assisted psychotherapy with autistic adults: a randomized, double-blind, placebo-controlled pilot study	MDMA	RCT	12 participants with autism and severe social anxiety received MDMA 75-125 mg or placebo. 31.3 ± 8.8(83.3%)	*Patients presented durable improvement in social anxiety symptoms. *In the MDMA group, one patient presented an intense anxiety reaction, and one patient presented an insomnia	LR; n.p.o.	Level 1
**Ot’alora et al. 2018** [137]	MDMA-assisted psychotherapy for treatment of chronic posttraumatic stress disorder: A randomized phase 2 controlled trial	MDMA	RCT	28 patients with PTSD received 2 actives doses (100, 125 mg) wit a low dose (40 mg) of MDMA. 42 ± 12.9(32.1%)	*Patient presented sleep-related reactions (insomnia) *22.2% of the 100 mg, and 7.7% of the 125 mg group presented irritability	LR; p.f., no coi	Level 1
**Jerome et al. 2020** [138]	Long-term follow-up outcomes of MDMA-assisted psychotherapy for treatment of PTSD: a longitudinal pooled analysis of six phase 2 trials	MDMA	RCT	107 participants received 2–3 doses (75-125 mg) MDMA psychotherapy sessions. 40.5 ± 10.63 (42.5%)	*The large majority of patients were improved. However, 2.4% of patients presented increased anxiety and avoidance of people or places	LR; n.p.o.	Level 1
**Mitchell et al. 2021** [139]	MDMA-assisted therapy for severe PTSD: a randomized, double-blind, placebo-controlled phase 3 study	MDMA	RCT	90 participants with severe PTSD received 80 + 40 mg and120 + 60 mg of MDMA or placebo 41 ± 11.9 (34.4%)	*No increase in adverse events related to suicidality or psychosis was observed *4.3% and 6.8% of the MDMA and the placebo group respectively presented suicidal ideation during the sessions	LR; n.p.o.	Level 1
**Ponte et al. 2021** [140]	Sleep Quality Improvements After MDMA-Assisted Psychotherapy for the Treatment of Posttraumatic Stress Disorder	MDMA	RCT	63 participants received 2–3 sessions of MDMA (75-125 mg) or placebo (0-40 mg) during psychotherapy sessions. 40.6 ± 11.3(53.9%)	*Patient presented sleep-related reactions (insomnia)	LR; n.p.o.	Level 1
**Brewerton et al. 2022** [141]	MDMA-assisted therapy significantly reduces eating disorder symptoms in a randomized placebo-controlled trial of adults with severe PTSD	MDMA	RCT	89 participants with severe PTSD received MDMA 80-180 mg in three experimental sessions over 8 weeks. 41.0 ± 12(34.8%)	*No increase in adverse events related to suicidality or psychosis was observed	LR; p.f., n.p.o.	Level 1
**Nicholas et al. 2022** [142]	The Effects of MDMA-Assisted Therapy on Alcohol and Substance Use in a Phase 3 Trial for Treatment of Severe PTSD	MDMA	RCT	90 participants with severe PTSD were randomized to 3 blinded trauma-focused therapy sessions with either MDMA-AT (80 + 40 /120 + 60 mg) or placebo+therapy. 44.4 ± 12.2(35.4%)	*No psychotic reactions are reported	LR; p.f., n.p.o.	Level 1
**Pacey et al. 2022** [143]	A Randomized, Double-Blind, Controlled Phase 2 Pilot Study of Manualized 3,4-MDMA-Assisted Psychotherapy in 12 Subjects With TR-PTSD	MDMA	RCT	12 individuals with TR-PTSD received 2 sessions of 125 mg MDMA-assisted psychotherapy or placebo with therapy. 47.7 ± 6.2(50%)	*Two patients presented depression and anxiety reactions and one participant presented insomnia during the first session of MDMA.	HR; n.p.o.	Level 1
**Wolfson et al. 2022** [144]	MDMA-assisted psychotherapy for treatment of anxiety and other psychological distress related to life-threatening illnesses: a randomized pilot study	MDMA	RCT	18 patients received MDMA (125 mg) or placebo in combination with two 8-h psychotherapy sessions. 54.9 ± 7.9(22%)	*No psychotic reactions are reported	LR; n.p.o.	Level 1
**Mitchell et al. 2023** [25]	MDMA-assisted therapy for moderate to severe PTSD: a randomized, placebo-controlled phase 3 trial	MDMA	RCT	Participants were randomized to MDMA-AT (n = 53) or placebo with therapy (n = 51). 39.1 ± 10(28.2%)	*Psychiatric treatment emergent adverse events were mostly mild to moderate; three severe events occurred in the MDMA-AT group (5.7%; n = 1 each: dissociation, flashback and grief reaction) and two in the placebo with therapy group (3.9%; n = 1 each: agitation and anxiety)	LR; n.p.o.	Level 1
**5. Multiple substances**							
**5.1. UCT and cohort studies**							
**UCT**							
**Hoch, Cattell & Pennes 1952** [14]	Effects of mescaline and LSD (d-LSD-25)	Mescaline LSD	NRCT	59 patients with schizophrenia received: mescaline (IV, 0.4 to 0.6 gm)(n = 17), d-LSD-25 (oral LSD 10 to 120 µg) (n = 21)(or both, n = 21). Patients were not benefiting from a medication. n.a.(40.7%)	*Patients with paranoid and catatonic schizophrenia experienced worsening symptoms during drug use. Most, but not all, pseudoneurotic schizophrenia patients showed improvement *Intravenous mescaline caused a more severe but temporary deterioration compared to oral LSD, with primarily visual hallucinations and some sexual content *LSD often led to emotional disturbances, and both drugs increased disorganization *The authors note a low incidence of auditory hallucinations, which contrasts sharply with the high rate seen in drug-free schizophrenic patients. Visual hallucinations, however, were more common and aligned with typical schizophrenic experiences *With LSD, 4 out of 21 patients experienced significant deterioration during their trip	CR; n.m.	Level 2
**Hollister et al. 1969** [145]	A Controlled Comparison of Lysergic Acid Diethylamide (LSD) and Dextroamphetamine in Alcoholics	LSD	RCT	29 patients with alcohol use disorders received up to 200-400 μg of LSD, 500 mg mescaline, or dextroamphetamine 60 mg. 45(100%)	*Authors report over 6 months of LSD sessions that only two patients were sufficiently agitated to require intramuscular administration of 50 mg. of chlorpromazine *One case of moderate confusion with more than 72 h of hospitalization is describe, but with no mention of psychotic symptoms	CR; n.m.	Level 2
**Cohort studies**							
**Bowers 1977** [49]	Psychosis precipitated by psychotomimetic drugs: a follow-up study.	LSD, mescaline, amphetamine	Prospective follow-up cohort	Follow-up study (at 1.9 to 5.8 years) of 15 patients with LSD psychosis. 21.2 ± 3.83 (26%)	*The best predictors of poor outcomes were withdrawal, motor retardation, blunt affect, and suicidal thoughts *Prolonged or recurring psychosis after LSD use may be linked to an inherent vulnerability to developing psychosis with affective features *The authors reported a good response to lithium carbonate and ECT in patients with LSD-induced psychosis	CR; n.m.	Level 2
**Breakey, Coodell & Lorenz et al. 1974** [146]	Hallucinogenic drugs as precipitants of schizophrenia	LSD (50%) Mescaline (28%)	Retrospective cohort of hospitalized patients	46 patients with schizophrenia and 46 controls that were hospitalized in psychiatry. 22.6 ± 3.7(60.8%)	*26 of 46 patients with schizophrenia (56%) to have used multiple drugs before the onset of their symptoms *Patients with schizophrenia experienced the onset of symptoms on average four years earlier than non-users and were also admitted to hospital four years earlier *Some patients were diagnosed with chronic SIPD rather than a PPD	CR; p.f.	Level 4
**Boutros et al. 1998** [147]	Chronological association between increases in drug abuse and psychosis in Connecticut state hospitals	Mainly LSD (combined with alcohol)	Retrospective cohort	Authors examine the admissions rate of drug abuse and schizophrenia from Connecticut state hospitals (1965 to 1983). 51.6±n.a.(91.8%)	*A rapid increase in new schizophrenia admissions coincided with a peak period for drug-related admissions. Authors discuss this association *No correlations were found between the drug-related total admissions and psychosis	CR; n.m.	Level 4
**Shoval et al. 2007** [148]	Substance Use, Suicidality, and Adolescent-Onset Schizophrenia: An Israeli 10-Year Retrospective Study	Various substances	Retrospective cohort	Authors included 178 adolescents with schizophrenia or schizoaffective disorders	*Lifetime use of LSD was associated to a greater likelihood suicide attempt: OR = 5.7; p = 0.002 (not adjusted) *Lifetime use of a hallucinogen was associated with an increased likelihood of past-year suicide ideation: aOR = 1.8, 95% CI 1.6–2.1, p < 0.0001	CR; no coi	Level 3
**Carstairs & Lee 2010** [74]	Peyote and mescaline exposures: a 12-year review of a statewide poison center database	Peyote and Mescaline	Retrospective study	Retrospective review of the California Poison Control system database for the years 1997-2008. 31 single-substance exposures to peyote or mescaline. 23 ± 11.25(84%)	*Thirty (97%) exposures were intentional; and through the oral route, whereas one patient (3%) insufflated mescaline powder. Five patients (16%) were managed at home, and the rest in a healthcare facility *Life-threatening symptoms did not occur, and most exposures were associated with only mild to moderate clinical effects (tachycardia, central nervous system effects). *Symptoms typically resolved within 24 h or less and did not usually require anything more than supportive measures or sedation *2 patients presented paranoia reactions and 2 patients’ psychosis	CR; no coi	Level 3
**Krebs & Johansen 2013** [57]	Psychedelics and mental health: a population study	LSD, psilocybin, mescaline, peyote	Population-study	Data drawn from years 2001 to 2004 of the National Survey on Drug Use and health consisted of 21967 respondents reporting lifetime psychedelics. n.a(61%)	*21,967 respondents (13.4%) reported lifetime psychedelic use, with the diagnosis of non-affective psychosis given to 4.4% of psychedelic users (129/2943) *Lifetime psychedelic use was significantly associated with: a lower rate of one of the seven psychotic symptoms (“Felt a force taking over your mind”: OR 0.7, p = 0.03); a lower rate of symptoms of panic attacks (aOR 0.9, p = 0.006) *Lifetime LSD use associated with a lower rate of outpatient mental health treatment (OR 0.9, p = 0.002) and psychiatric medication prescription (OR 0.9, p = 0.04) *Lifetime psilocybin use associated with a lower rate of inpatient mental health treatment (OR 0.8, p = 0.04) and psychiatric medication prescription (OR 0.8, p < 0.001) *Lifetime mescaline/peyote use associated with: a lower rate of psychiatric medication prescription (OR 0.8, p = 0.004) and no mental health treatment (OR 0.8, p = 0.001); with a lower rate of symptoms of agoraphobia (aOR 0.6, p = 0.005) *Lifetime peyote use associated with a lower rate of psychiatric medication prescription (OR 0.8, p = 0.01) *Lifetime psilocybin use and lifetime mescaline/ peyote use associated with a lower rate of one of the specific psychotic symptoms (“Felt a force taking over your mind”: psilocybin, aOR 0.6, p = 0.004; mescaline/peyote: aOR 0.7, p = 0.04)	CR; p.f, no coi	Level 3
**Hendricks et al. 2014** [149]	Hallucinogen use predicts reduced recidivism among substance-involved offenders under community corrections supervision	LSD Mescaline Psilocybin	Register based study 2002-2007	130,152 respondents, randomly selected to be representative of the adult population in the USA, with 25,622 individuals incarcerated in the Southeastern US. n.a(62.8%)	*Hallucinogen use disorder was associated with a decreased probability of supervision failure while controlling for an array of potential confounding factors (OR) = 0.60 (0.46, 0.79), in contrast with all other substance use disorders *Hallucinogens may promote alcohol and other drug abstinence and prosocial behavior in a population with high rates of recidivism	CR; no coi	Level 3
**Hendricks et al. 2014** [150]	Classic psychedelic use is associated with reduced psychological distress and suicidality in the United States adult population	LSD Mescaline Psilocybin	Register based study	Relationships of classic psychedelic use with psychological distress and suicidality among over 190,000 USA adult respondents pooled from the National Survey on Drug Use and Health (2008–2012). n.a(62.8%)	*Lifetime classic psychedelic use was associated with a significantly reduced odds of past month psychological distress weighted odds ratio (OR) = 0.81 (0.72-0.91), past year suicidal thinking (OR = 0.86 (0.78-0.94)), past year suicidal planning (OR = 0.71 (0.54-0.94)), and past year suicide attempt (OR = 0.64 (0.46-0.89)) *Lifetime illicit use of other drugs was largely associated with an increased likelihood of these outcomes	SR; no coi	Level 3
**Johansen & Krebs 2015** [59]	Psychedelics not linked to mental health problems or suicidal behavior: a population study.	Various substances	Population study	135095 randomly selected US adults, including 19299 psychedelics users	*19299(14.28%) of the sample reported lifetime psychedelics use *Lifetime psychedelic use was associated with a lower likelihood of past year inpatient mental health treatment: aOR 0.8, p = 0.01 *Associations between psilocybin use and lower likelihood of past year serious psychological distress, inpatient mental health treatment and psychiatric medication prescription: aOR 0.9, p = 0.007; aOR 0.7, p = 0.004; aOR 0.8, p = 0.002 *Past year use of LSD was associated with lower likelihood of serious psychological distress: aOR 0.8,p = 0.04 *Among people with a history of childhood depressive episode, psychedelic use was associated with a lower likelihood of suicidal thoughts: aOR 0.8, p = 0.01, and suicidal plan: aOR 0.5, p = 0.005	CR; p.f, no coi	Level 3
**Vallersnes et al. 2016** [151]	Psychosis associated with acute recreational drug toxicity: A European case series.	LSD Psilocybin	Register based study	The European Drug Emergencies Network collects data on presentations to emergency departments with acute recreational drug and NPS toxicity at 16 centers in ten countries. 109 cases.	*Psychosis was frequent in those ED presentations involving tryptamines (4/7; 57.1%), methylenedioxypyrovalerone (6/22; 27.3%), methylphenidate (6/26; 23.1%), LSD (18/86; 20.9%), psilocybe mushrooms (3/16; 18.8%), synthetic cannabinoid (4/26; 15.4%) and amphetamine (87/593; 14.7%), but less common in those involving mephedrone (14/245; 5.7%), MDMA (20/461; 4.3%) and methedrone (3/92; 3.3%). *These results should be examined in the light of important limitations (data on previous psychiatric diagnoses not collected, no follow-up data, diagnosis made by an emergency department clinician)	CR; p.f., no coi	Level 3
**Starzer et al. 2018** [80]	Rates and predictors of conversion to schizophrenia or bipolar disorder following substance-induced psychosis.	Various hallucinogens	Register based study	Information extracted from the Danish Civil Registration System. 6788 patients with a diagnosis of SIP. n.a(78%)	*The highest conversion rate was found for cannabis-induced psychosis, with HR = 47.4% (95% CI 54.7–52.3) converting to either schizophrenia or bipolar disorder *Alcohol induced 34% of the psychoses, cannabis 22%, and mixed/other substances 27%	CR; p.i.	Level 2
**Rognoli et al. 2023** [81]	Transition From Substance-Induced Psychosis to Schizophrenia Spectrum Disorder or Bipolar Disorder	Various hallucinogens	Register based study	Information extracted from the Norwegian Patient Registry from 2010 to 2015. 3187 patients with a diagnosis of SIP.33.6 ± 12.3(73.4%)	*The highest conversion rate was found for Cannabis-induced psychosis CH = 36%(95% CI 31.4-41) converting to schizophrenia *The risk of transition of psychedelics-induced psychosis was obtained directly	SR; p.f.	Level 2
**Evans et al. 2023** [152]	Extended difficulties following the use of psychedelic drugs: A mixed methods study	Various hallucinogens	Web-based questionnaires	This mixed-method study focus on self-report of long-lasting side-effects following hallucinogens use in 608 participants	*The most common forms of extended difficulty were feelings of anxiety and fear, existential struggle, social disconnection, depersonalization and derealization *For approximately one-third of the participants, problems persisted for over a year, and for a sixth, they endured for more than three years *A shorter duration of difficulties was predicted by knowledge of dose, drug type and lower levels of difficulty reported during the psychoactive experience, while a narrower range of difficulties was predicted by taking the drug in a guided setting. *5%(29/608) of participants reported long-lasting psychotic symptoms	CR; p.f.	Level 3
**Simonsson et al. 2023** [153] **(Honk et al. 2024** [89]**) duplicate**	Longitudinal associations between psychedelic use and psychotic symptoms in the United States and United Kingdom	Various hallucinogens	Longitudinal study	Authors included samples representative of the US and UK adult populations with regard to sex, age, and ethnicity (N = 9732)	*A total of 7667 patients completed the follow-up survey. Among the 100 who used psychedelics during the 2-month study, psychedelic use was linked to increased unusual visual experiences *Psychedelic use generally did not affect psychotic symptoms. However, in individuals with a personal or family history of bipolar disorder, it was associated with increased psychotic symptoms *In individuals with a personal history of psychotic disorders (but not family history), psychedelic use was linked to a reduction in psychotic symptoms *The authors suggest that while psychedelics may reduce or have no effect on the risk of psychosis in those with a personal or family history of psychotic disorders, they may increase the risk of mania with psychotic features in those with a personal or family history of bipolar disorder	CR; p.f.	Level 2
**5.2. RCT**							
**RCT**							
**Isbell 1959** [67]	Comparison of the reactions induced by psilocybin and LSD-25 in man	Psilocybin LSD	RCT	Seven volunteers ingested 1.0 and 1.5 mcg/kg of LSD, and 114 mcg/kg of psilocybin (4, 6 and 8 mg/70 kg) 22-40(100%)	*Hallucinations and mood alterations were more present with LSD *LSD is approximately 100-150 times as potent as psilocybin *The effects of psilocybin did not persist as long as those of LSD *Two patients lost their insight during the psychedelic trip and felt that their experiences were caused by the experimenters controlling their minds (2/7)	SR; no coi	Level 2
**Wolbach, Miner & Isbell 1962** [68]	Comparison of psilocin with psilocybin, mescaline and LSD-25.	LSD Mescaline Psilocybin	RCT	Ten former morphine addicts received IM: LSD (0.75–1.5 mcg/kg), mescaline (2.5–5 mg/kg), psilocybin (75–150 mcg/kg), or a placebo 25–33(n.a.)	*The time course of the psilocin and psilocybin reactions are shorter than those of LSD or mescaline reactions *1 mcg/kg of psilocybin base was equivalent to 1.48 mcg/kg of psilocybin base (95% CI, 1.08 to 2.05 mcg/kg)	CR; no coi	Level 1

To note, high risk of the ROB2 scale are categorized as ‘CR’ critical risk in the table.

*AMSTAR Checklist* A Measurement Tool to Assess systematic Reviews checklist, *CC* case-control, *Loe OCEBM* level of evidence according to the Oxford Center for Evidence-Based Medicine (Levels of Evidence Working Group), *n.m.* not mentioned, *no coi* no conflict of interest, *npo* nonprofit organization or trusts (e.g. Heffter Research Institute), *NRCT* non-randomized clinical trial, *pf* public funds (e.g. grants, state funds), *pi* private industry (e.g. pharmaceutical compagnies), *RCT* randomized clinical trial, *SANRA Scale* Scale for the Assessment of Narrative Review Articles, *UCT* uncontrolled studies.

**Table 2:** Narrative reviews and systematic reviews reporting potential psychedelic-induced psychosis.

Reviews							
Author	Title	Drug	Type of study	Setting	Main findings	AMSTAR Checklist /SANRA scale /COIs	LoE OCEBM Levels of Evidence
**I. Reviews on long-lasting psychotic reactions - transition to psychosis**							
**1. LSD**							
**1.1. Narrative reviews**							
**Cohen** [37]	Lysergic acid diethylamide: side effects and complications	LSD	Narrative review and report of a UCT	Follow-up cohort Case reports. A total of 62 physicians who had experience with prescription of LSD or mescaline were asked on psychiatric side effects of treatments. (n.a)	*Four adverse reactions following LSD use were identified: ‘prolonged psychotic reactions’, ‘acting out behavior’, ‘abuse of euphoriant’, ‘multihabituation’. *Rate of 1.8 prolonged reactions per 1000 patients. *Suicide attempts and completed suicides occurred at a rate of 1.2 and 0.4 per 1000 patients. *No case of HPPD were detect on this sample of 5000 LSD users.	MQ; n.m.	Level 2
**Smart & Bateman** [154]	Unfavorable reactions to LSD: a review and analysis of the available case reports.	LSD	Narrative review	21 case-reports which contained the details of 225 adverse reactions. Additional citation of longitudinal studies	*The most serious complications include prolonged psychotic reactions, recurrent LSD experiences, disturbed non-psychotic reactions, and, less frequently, suicide, homicide, and convulsions. *Data on the frequency of illicit use are unavailable, so prevalence rates of adverse reactions cannot be estimated *Baker [76] (conference) precipitated four psychoses lasting three or four days in 150 patients who received up to 10 LSD sessions. *Leuner [155] (conference) found three prolonged psychotic reactions among 82 patients given an average of 27 LSD sessions.	LQ; n.m.	Level 2
**Panhuysen** [156]	[Undesirable side effects of LSD administration]	LSD	Narrative review	Authors discuss side-effects associated with LSD use	*Illegally manufactured LSD can be contaminated with atropine-like acting substances *Patients with the following conditions should only be eligible for LSD trials after intense clinical examination: -Patients with neurotic ego-defense mechanisms at work -Patients with psychasthenic symptoms, psychopathic traits, with bipolar disorders, schizoaffective disorders and schizophrenia.	LQ; n.m.	Level 3
**Strassman** [7]	Adverse reactions to psychedelic drugs. A review of the literature	LSD	Narrative review	Good quality narrative review including case series, cohorts’ studies and clinical trials	*Strassman distinguish from acute, time-limited panic reactions during administration, through transient psychoses lasting several days, to recurrent flashbacks and chronic undifferentiated psychotic and treatment-resistant cases. *Potential risk are: poorer premorbid adjustment, a history of psychiatric illness and/or treatment, a greater number of exposures to psychedelic drugs and correlatively, a greater average total cumulative dosage taken over time, drug-taking in an unsupervised setting, a history of polydrug abuse, and self-therapeutic and/or peer-pressure-submission motive *Discussion of the potential of LSD to trigger underlying illness	HQ; n.m.	Level 2
**Novak** [79]	LSD before Leary: Sidney Cohen’s critique of 1950s psychedelic drug research	LSD	Narrative review	Narrative review on historical concepts	*Reports a long-lasting psychosis ( > 48H) at a rate of 1 case out of 247 individuals who received LSD	LQ; n.m.	Level 3
**Paparelli et al**. [86]	Drug-induced psychosis: how to avoid star gazing in schizophrenia research by looking at more obvious sources of light	LSD	Narrative review	Narrative review on mechanism of drug-induced psychosis	*Authors discuss the link between cannabis use and psychosis *Authors propose that stimulants and THC are more likely to induce paranoia beliefs, in particular following repeated use, whereas LSD is more closely associated with visual hallucinations	H.Q.; no coi	Level 2
**1.2. Systematic review**							
**Boutros & Bowers** [147]	Chronic substance-induced psychotic disorders: state of the literature.	LSD	Systematic review	Case series, cohorts’ studies and case controls studies	*Psychostimulants, hallucinogens, marijuana, and possibly industrial inhalants can cause or increase the susceptibility for a state of chronic psychosis	CL n.m.	Level 1
**De Gregorio et al.** [157]	d-Lysergic Acid Diethylamide (LSD) as a Model of Psychosis: Mechanism of Action and Pharmacology	LSD	Systematic review	Systematic review on preclinical studies regarding the mechanism of action involved in the psychotic-like effects induced by LSD	*LSD’s exert effects on serotonin, dopamine, glutamate and TAAR systems *LSD’s mechanism of action is pleiotropic, primarily mediated by the serotonergic system in the Dorsal Raphe, binding the 5-HT2A receptor as a partial agonist and 5-HT1A as an agonist. *LSD also modulates the Ventral Tegmental Area, at higher doses, by stimulating dopamine D2, Trace Amine Associate receptor 1 (TAAR1) and 5-HT2	CL no coi	Level 1
**Perez et al**. [22]	Psilocybin-assisted therapy for depression: A systematic review and dose-response meta-analysis of human studies	Psilocybin	Meta-analysis	A total of 366 patients included in several RCt were included in this meta-analysis. Doses of psilocyin varied from 3 mg to 40 mg. 366(46.8%)	*The determined 95% effective doses per day (ED95) were 8.92, 24.68, and 36.08 mg/70 kg for patients with secondary depression, primary depression, and both subgroups, respectively. *Authors found found significant dose-response associations for various side effects, including physical discomfort, blood pressure increase, nausea/vomiting, headache/migraine, and the risk of prolonged psychosis	LR; p.f.	Level 1
**2. DMT**							
**2.1. Narrative reviews**							
**Jacob & Presti** [158]	Endogenous psychoactive tryptamines reconsidered: an anxiolytic role for dimethyltryptamine	DMT	Narrative review	Narrative review on the role of DMT	*Authors hypothesis that the action of endogenous DMT at the TA receptor is to produce a calming, anxiolytic effect, which may suppress, rather than promote, symptoms of psychosis.	MQ; n.m.	Level 4
**Grammenos & Barker** [159]	On the transmethylation hypothesis: stress, N,N-dimethyltryptamine, and positive symptoms of psychosis	DMT	Narrative review	Narrative review on the role of DMT	*Stress has been found to elevate DMT levels in rodents and DMT levels have been associated with positive features of psychosis. *Healthy participants treated with exogenous DMT experience predominantly positive symptoms of psychosis. Authors hypothesize that increased DMT reactivity as a response to stress could possibly underlie positive symptoms of psychosis in a subgroup of patients with schizophrenia.	MQ; no coi	Level 4
**2.2. Systematic reviews**							
**Gable** [160]	Risk assessment of ritual use of oral dimethyltryptamine (DMT) and harmala alkaloids	DMT	Systematic review and presentation of case reports	Narrative review on the role of DMT	*DMT is capable of inducing aversive psychological reactions or transient psychotic episodes that resolve spontaneously in a few hours. *The dependence potential of oral DMT and the risk of sustained psychological disturbance are minimal. *DMT or ayahuasca experience has a substantial degree of unpredictability with respect to both aversive and positive aspects, depending on variables such as dosage, participant’s intention and setting.	CL; npo	Level 2
**Dos Santos et al**. [55]	Ayahuasca, dimethyltryptamine, and psychosis: a systematic review of human studies	DMT	Systematic review and presentation of one case report	Systematic review on DMT and psychosis	*Authors suggest that the incidence of psychotic episodes associated with ayahuasca/DMT intake is a rare phenomenon, and these rare instances appear be associated with previous premorbid characteristics of the individuals, previous and possibly concurrent drug abuse, and lack of a supervised setting *Individuals with personal or family history of schizophrenia or schizophreniform disorders, psychotic depression or mania, or with ongoing manic or psychotic symptomatology, should avoid ayahuasca/DMT intake	CL; no coi	Level 2
**Orsolini et al**. [87]	How does ayahuasca work from a psychiatric perspective? Pros and cons of the entheogenic therapy	Ayahuasca	Systematic review	Systematic review on preclinical, observational, and experimental studies in healthy volunteers and in clinical samples	*Ayahuasca appears to be safe and well tolerated, nausea and emesis being the most reported and transient side effects. *Findings suggest not to use ayahuasca in bipolar or psychotic patients because of an increased risk of manic switch -based on 2 case-reports- and/or psychotic onset -based on one epidemiological survey- that do not drive clear conclusion on this association (see Lima & Tófoli, 2011)	LQ; no coi	Level 1
**3. Mescaline**							
**Studerus et al**. [75]	Acute, subacute and long-term subjective effects of psilocybin in healthy humans: a pooled analysis of experimental studies	Psilocybin	Review of different clinical trials	Narrative review of the prospective follow-up of patients included in different clinical trials. 227 subjects experimented psilocybin with follow-up of possible adverse events	*Authors identified a pool of 8 RCTs published between 1999 and 2008 *The analysis included 110 healthy subjects who had received 1–4 oral doses of psilocybin (45-315 µg/kg body weight) *Acute adverse drug reactions, characterized by strong dysphoria and/or anxiety/panic, occurred only in the two highest dose conditions in a relatively small proportion of subjects (2/110) *Authors found no indication for subsequent drug abuse, persisting perception disorders, prolonged psychosis or other long-term impairments of functioning in any of our subjects.	LR; npo, pf	Level 1
**4. MDMA & Ecstasy**							
**4.1. Narrative reviews**							
**Skryabin** [161]	Hallucinogen persisting perception disorder: A literature review and three case reports	MDMA Ecstasy	Narrative review	Narrative review on the phenomena of HPPD with case report presentation	*Authors discuss HPPD II occurrence in users of ecstasy and MDMA. *Treatments with tofisopam, lamotrigine and sertraline showed possible partial response.	HQ; no coi	Level 3
**McGuire et al.** [162]	Long term psychiatric and cognitive effects of MDMA use.	MDMA	Narrative review	Narrative review gathering 16 case reports and case series	*In rare cases, MDMA use may be associated with chronic psychiatric symptoms, which persist long after the cessation of MDMA use, such as psychotic features, panic disorder, depression, and obsessive-compulsive symptoms *These subjects might already be predisposed to psychiatric disorders. *50% of cases in a series of MDMA users with chronic psychiatric symptoms had a first degree relative with a psychiatric illness, and 50% had previously experienced transient psychiatric symptoms following use of other illicit drugs. *Severe long-term psychiatric disturbances following MDMA use seem uncommon relative to the large numbers of people who use MDMA *It is difficult to determine whether MDMA use is directly responsible, triggers symptoms in subjects predisposed to mental illness, or is incidental.	HQ; n.m.	Level 2
**Soar et al.** [163]	Psychiatric disorders in Ecstasy (MDMA) users: A literature review focusing on personal predisposition and drug history.	MDMA Ecstasy	Narrative review	This narrative review present 38 case reports of side-effects following MDMA or ecstasy use	*Authors estimate that 5 million individuals have tried Ecstasy in UK *29% of cases showing psychiatric symptoms after MDMA consumption involved psychotics symptoms, with 65% of psychotic symptoms among presented case-reports *Authors propose that MDMA could cause long-term neurotoxicity *24% of the patients had a previously diagnosed psychiatric history and 34% had a family psychiatric history	HQ; n.m.	Level 3
**4.2 Systematic reviews**							
**Smith et al.** [164]	MDMA-Assisted Psychotherapy for Treatment of Posttraumatic Stress Disorder: A Systematic Review With Meta-Analysis	MDMA	Systematic review and meta-analysis	This meta-analysis included 5 RCTS of MDMA-assisted psychotherapy	*Therapy was generally safe and well tolerated, bruxism, anxiety, jitteriness, headache, and nausea are commonly reported. *Moderate to high dose of MDMA could expose to panic attacks and anxiety, in particular in a non-controlled psychotherapeutic setting	LQ; no coi	Level 1
**5. Multiple substances**							
**5.1. Narrative review**							
**Mogar & Aldrich** [165]	The use of psychedelic agents with autistic schizophrenic children.	LSD, Psilocybin and UML	Narrative review	Narrative review on 7 studies gathering 91 patients with severe autism resistant to treatments	*80% of patients presented some improvement with LSD *Doses ranged from 50 to 400 μg. The most effective dose was of 100 μg with improved speech behavior in mute children; increased emotional response to other children and adults; elevation in positive mood, decreases in compulsive ritualistic behavior.	MQ; n.m.	Level 2
**Glass** [166]	Psychedelic drugs, stress, and the ego. The differential diagnosis of psychosis associated with psychotomimetic drug use	Mescaline and LSD	Narrative review and case series	Narrative review including case series, with the case report of a patients with chronic psychosis associated with 200 LSD trips in a 2-year period	*Clinical entities to distinguish psychotic onset and psychedelic induced lasting symptoms: past personal history, current and past drug use, prepsychotic level of functioning, mode of onset of long-lasting symptoms, nature of external precipitating factors and presenting mental status *Occasional drug use in patients with schizophrenia may have no significant impact on the patients overall clinical course or response to treatment	LQ; n.m.	Level 3
**McCabe** [167]	Psychedelic Drug Crises: Toxicity and Therapeutics	All psychedelics	Narrative review	Narrative review of psychedelic mechanisms	*The author propose that obsessive and paranoid personality orientations represent the defensive styles most at risk under the influence of psychedelics. *The author proposes a typical sequence that occurs during the psychedelic trip: altered perceptions of external and internal physical stimuli; a loosening of psychological defenses, often after an intensification or caricaturization of those defenses, with a resulting release of conflictual unconscious material; and dissolution of rational, logical, and problem-solving functions (“ego”), with an alteration in the perception of time, space, and subject-object dichotomies.	LQ; n.m.	Level 3
**Vollenweider et al**. [9]	Psilocybin induces schizophrenia-like psychosis in humans via a serotonin-2 agonist action.	Psilocybin LSD	Review of two clinical trials	Authors describes two experiments in healthy human volunteers, to examine the psychotomimetic effects of psilocybin and its response to antipsychotic.	*D2 antagonist haloperidol attenuated psilocybin-induced depersonalisation, derealisation and euphoria *Haloperidol had virtually no effect on psilocybin-induced hallucinations (VUS) and even increased anxious ego-dissolution (AIA) in psilocybin subjects, *Ketanserin, a preferential 5-HT2A receptor antagonist, dose-dependently prevented psilocybin psychosis	MQ; pf	Level 1
**Wolf et al.** [8]	Could psychedelic drugs have a role in the treatment of schizophrenia? Rationale and strategy for safe implementation	All psychedelics	Narrative review	Narrative review of the potential of psychedelics in the treatment of schizophrenia	*Psychedelics could have a therapeutic potential on reducing primary negative symptoms due to the ability to enhance neural plasticity and its effects on inflammatory processes	HQ; pf, npo	Level 1
**5.2. Systematic review**							
**Hermle et al.** [168] (Article in German)	Hallucinogen-induced psychological disorders	All hallucinogens	Systematic review	Narrative review on Hallucinogen-induced disorders	*The authors reported that adolescent intoxication with psychedelic drugs rarely produced acute psychotic syndromes	M.Q.; n.m.	Level 2
**Trope et al.** [169]	Psychedelic-assisted group therapy: A systematic review	RCTs settings	Systematic review	Authors discuss psychedelic-assisted individual psychotherapy modalities	*Authors discuss group therapies for “neurotics” that include patients with psychosis. Most studies excluded chronically psychotic patients, however patients with comorbid psychosis were frequently included *In most studies, the assessment and reporting of adverse events is inconsistent * No studies reported any cases of prolonged psychosis, suicide, or other serious adverse events directly attributable to psychedelic administration	CL; pf	Level 1
**Fiorentini et al**. [27]	Substance-Induced Psychoses: An Updated Literature Review	All hallucinogens	Systematic review	Authors present and discus substance-induced psychoses for different hallucinogens	*The propensity to develop psychosis seems to be a function of the severity of use and addiction * There remains a striking paucity of information on the outcomes, treatments, and best practices of substance-induced psychotic episodes.	LQ; pf	Level 2
**6. Reviews on long-lasting psychosomatic reactions**							
**6.1. Narrative reviews**							
**McCabe** [167]	Psychedelic drug crises: toxicity and therapeutics.	All psychedelics	Narrative review	Authors discuss mechanisms, psychedelic experiences, risks, and potential treatments of adverse effects	*Acute panic reactions and spontaneous recurrent experiences seem to be more common with psychedelic amphetamines *Author comment of a specific bad trip due to negative emotions and psychosomatic symptoms, that can last beyond the session	LQ; n.m.	Level 3
**7. Hallucinogen persisting perception disorder (LSD, MDMA, and other drugs)**							
**7.1. Narrative reviews**							
**Lerner et al.** [170]	Flashback and hallucinogen persisting perception disorder: clinical aspects and pharmacological treatment approach.	LSD	Narrative review	Narrative review on the phenomena of HPPD	*Flashback is a usually short-term, non-distressing, spontaneous, recurrent, reversible and benign condition accompanied by a pleasant affect *HPPD is a generally long-term, distressing, spontaneous, recurrent, pervasive, either slowly reversible or irreversible, non-benign condition accompanied by an unpleasant dysphoric affect. *Pharmacological agents such as clonidine, perphenazine and clonazepam some efficacy on HPPD	MQ; n.m.	Level 3
**Litjens et al.** [171]	Hallucinogen persisting perception disorder and the serotonergic system: A comprehensive review including new MDMA-related clinical cases.	MDMA	Narrative review	Narrative review on the phenomena of HPPF with presentation of case-reports	*Authors present 31 HPPD cases that implicated MDMA as a causative agent for HPPD-like symptoms, alongside classical hallucinogens. *According to authors HPPD symptoms may be a result from a misbalance of inhibitory-excitatory activity in low-level visual processing and GABA-releasing inhibitory interneurons may be involved	HQ; pf	Level 3
**7.2. Systematic reviews**							
**Halpern & Pope** [172]	Hallucinogen persisting perception disorder: what do we know after 50 years?	LSD	Systematic review	Systematic review on the phenomena of HPPD	*The term ‘flashbacks’ is poorly defined. Most studies provide little information to judge how cases could meet DSM-IV criteria for HPPD *Information about risk factors for HPPD, possible etiologic mechanisms, and potential treatment modalities must be interpreted with great caution *HPPD appears to be a genuine but uncommon disorder, sometimes persisting for months or years after hallucinogen use and causing substantial morbidity. It is reported most commonly after illicit LSD use, but less commonly with LSD administered in research or treatment settings, or with use of other types of hallucinogens	CL; npo, pf	Level 2
**Orsolini et al.** [173]	The “Endless Trip” among the NPS Users: Psychopathology and Psychopharmacology in the Hallucinogen-Persisting Perception Disorder. A Systematic Review	LSD	Systematic review	Authors gathered 45 papers on the HPPD phenomena, and discuss risk factors and treatment options	*Authors distinguish HPPD I (short term) and HPPD II (long-lasting) *A vast list of psychoactive substances has been identified and linked with the development of this condition	LQ; pf	Level 2
**Martinotti et al.** [174]	Hallucinogen Persisting Perception Disorder: Etiology, Clinical Features, and Therapeutic Perspectives	LSD Cannabis	Systematic review	Systematic review on the phenomena of HPPD	*The current prevalence estimates are unknown, but DSM-5 suggests 4.2% *The condition is more often diagnosed in individuals with a history of previous psychological issues or substance misuse and can occur even after a single use of LSD or other psychedelics *Controlled clinical investigations are mostly needed in order to better understand the etiology, mechanisms of action, clinical issues, and pharmacological treatment	CL; no coi	Level 2
**Murrie et al**. [20]	Transition of Substance-Induced, Brief, and Atypical Psychoses to Schizophrenia: A Systematic Review and Meta-analysis	Multiple substances	Systematic review and meta-analysis	Systematic review on the risk of substance-induced psychosis. Authors included 3 studies (n = 208) for hallucinogens.	*Authors propose a transition rate of ‘hallucinogens-induced psychosis’ of 26% (95%CI 14-43)(p = 0.0211; I^2^ = 74) *The rate of transition to schizophrenia was higher following cannabis-induced psychosis (34%) than other substance-induced psychoses, including those associated with amphetamines (22%) and hallucinogens (26%). *Of importance, one study concerned phencyclidine	HQ; no coi	Level 1
**Doyle et al. 2022** [175]	Hallucinogen persisting perceptual disorder: a scoping review covering frequency, risk factors, prevention and treatment	LSD	Scoping review	Scoping review on the phenomena of HPPD	*HPPD appears to be an uncommon, yet serious event associated with prior hallucinogen exposure. *One theory suggests that cell death of cortical GABAergic inhibitory neurons expressing serotonergic 5HT2A receptors, induced by toxicity related to the actions of LSD as well as other hallucinogens on these receptors, leads to disinhibition of the visual system at the cortical level, resulting in the onset of HPPD symptoms	CR; pf, pi	Level 2

### Evidence from UCT

#### Evidence from UCT: psychedelic-induced psychosis in healthy individuals

Two UCTs utilizing LSD were found, published from 1956 to 2023 (Table 1.1 and Table 2). These studies noted that a few psychopathology-free individuals can exhibit paranoid symptoms with reduced insight starting at 100 µg LSD doses, confined to the session [37, 38]. The method of administration does not appear to affect the occurrence of these symptoms. Cohen suggest a 1.8% rate of prolonged psychotic reactions after LSD use in healthy individuals. However, this author speculates whether these affected individuals had preexisting psychopathology.

These studies presented a low level of evidence with critical risks of bias at the ROB2 scale and at most Level 2 ranking at the OCEBM (Table 1).

For DMT, authors also describe paranoid symptoms in a minority of participants [39]. More recently, DMT has been studied in healthy individuals, with no reported psychopathological complications (Table 1.2.1).

Concerning psilocybin, authors noted dose-dependent psychotic-like symptoms (intense anxiety) and hallucinations in healthy volunteers [40]. An open-label study found that up to 22% of patients exhibited mild paranoia and ideas of reference during the sessions [41]. Finally, one recent state-of-the-art open-label study proposed psilocybin-assisted therapy (one session, 25 mg) to patients with bipolar type II depression [6]. Authors found no side-effects at the given dose for selected patients, however patients with psychotic features were excluded. The cross-sectional nature of these studies limits the quality of evidence, with most studies showing significant risk at evidence levels 2–3 according to OCEBM criteria.

### Evidence from UCT: use of psychedelics in patients with schizophrenia

We found eleven UCTs administering LSD sessions to adults with schizophrenia, including two studies involving treatment-resistant patients (Table 1.1.1). Oral LSD doses ranged from 30 to 500 μg for these stabilized patients. However, most studies did not mention whether patients were taking an antipsychotic medication. Given the publication dates spanning 1947 to 1971, it is likely that the majority underwent LSD sessions without concurrent medication -or only with D2 blockers- as no information on any medication was found in these articles.

Several studies indicate that individuals with schizophrenia exhibit ‘resistance’ to LSD effects in contrast to healthy volunteers [42–45]. Another study noted phenomenological aspects involving the intensification of preexisting symptomatology under LSD, frequently associated with heightened sexual motifs, increased psychomotor activity, and euphoria. Forrer and Goldner conducted a study administering LSD through intra-muscular injections to six patients across 42 sessions, revealing no notable distinction from the oral route [46]. In contrast, two studies employing intravenous LSD administration among patients with schizophrenia reported more distinct reactions [13, 47]. Indeed, in all studies, no long-lasting psychotic reactions were found, albeit in one study that administered intravenously LSD (0.5 µg/kg to 10 µg/kg) [13], after 1 to 3 sessions, 3 of 65 patients presented long-lasting psychotic reactions up a to a week after LSD sessions. Moreover, in another study with intravenous LSD, two patients presented marked fear during the session [47].

Hoch and colleagues in 1952 compared effects of LSD and mescaline in patients with schizophrenia [14], and found that patients with ‘pseudoneurotic’ and schizoaffective disorders improved (Table 1.6.1). A study involving ‘prepubertal children’ with schizophrenia [48] noted no side effects or significant emotional reactions to LSD at doses ranging from 50 to 150 μg. However, for both studies important methodological question arises regarding the accuracy of diagnoses. Additionally, we located four studies encompassing children with treatment-resistant severe autism accompanied by psychotic symptoms. LSD dosages varied between 40 and 400 µg. In nearly all these studies, short-term enhancements in speech and behavior, heightened emotional responsiveness, improved positive mood, and decreased compulsive ritualistic conduct were observed. All identified studies were rated with critical risk for bias on the ROB2 scale, yet with a level 2 OCEBM classification considering the specific population.

### Evidence from cohort studies

#### Evidence from cohort studies: substance-induced psychosis in previously healthy individuals

For LSD, we retrieved four cohort studies [49–52] (Table 1.1.1). McGlothlin & Arnold conducted a 10-year follow-up of 247 healthy individuals who underwent LSD sessions. They found one instance of prolonged psychosis after three LSD sessions, resulting in a week-long hospitalization. Bowers conducted a follow-up study of up to 5.8 years in 15 patients with LSD-induced psychosis and found that approximately half of the patients presented good outcomes. Vardy & Kay tracked 29 patients diagnosed with LSD-induced psychosis for up to five years [52]. During the follow-up, authors could not distinguish these patients from those experiencing a first episode of psychosis. Niemi-Pynttäri and colleagues retrospectively analyzed Finland’s nationwide hospital discharge register, discovering a cumulative 24% risk of receiving a schizophrenia spectrum diagnosis for patients with initial hallucinogen-induced psychosis [50].

For DMT, we found a 13-year epidemiological survey in a Brazilian indigenous community where regular ayahuasca users ( > 50 uses per year) were studied [53]. Notably, almost no cases of persistent psychotic phenomena were reported (estimated at 1 in 50,000).

Similarly, in the case of psilocybin, a 4-year population survey among Navajo Indians who were frequent peyote users documented isolated incidents of enduring psychotic symptoms (estimated at 1 in 70,000) [54]. In 2017, Dos Santos and colleagues suggested that the occurrence of psychotic episodes linked to ayahuasca/DMT intake is rare, and these infrequent cases seem to be related to preexisting traits (e.g.; family history of psychosis, concomitant use of other drugs), prior and possibly concurrent substance abuse, and a lack of supervised settings [55].

For ecstasy, a prospective cohort study tracked patients diagnosed with ecstasy-induced psychotic disorders and estimated that 10% of these patients had previously experienced psychotic symptoms [56]. Various population studies were also identified. While most studies are of notably low quality, characterized by critical risk and Level 4 OCEBM ratings (Table 1.4.1), some higher-quality studies reported a 4.4% occurrence of non-affective psychosis diagnosis among psychedelic users [57–59] Intriguingly, lifetime psychedelic use was linked to a reduced likelihood of undergoing inpatient mental health treatments in the past year (adjusted odds ratio 0.7; *p* = 0.01). These cohorts’ studies presented moderate to critical ROB2 rating and were classified as level 2 OCEBM studies.

### Evidence from cohort studies: use of psychedelics in patients with schizophrenia

No cohort study on the use of psychedelics in patients with schizophrenia was identified.

### Evidence from case-control studies

#### Evidence from case-control studies: substance-induced psychosis in healthy individuals

In 1968, Ungerleider suggested that up to one third of patients with LSD-induced psychosis might have schizophrenia [60]. In a separate case-control study, Hays & Tilley proposed that LSD-induced psychosis differs from schizophrenia, having fewer primary delusions and auditory hallucinations, more visual hallucinations, and less emotional blunting [61].

For MDMA, one case-control study proposed that patients with recent use of MDMA presented less blunted affect and more hostile behavior. Rare cases of psychosis with high levels of aggressiveness and violence are reported [62].

### Evidence from case-control studies: use of psychedelics in patients with schizophrenia

One case-control study compared patients with schizophrenia and schizoaffective disorder using LSD to healthy individuals [63] (Table 1.1.2). Sloan and Doust’s [63] study reported the safety of 40–120 μg LSD doses and improved mood in unmedicated patients with schizophrenia. This study carried a critical risk for bias and a Level 2 OCEBM classification.

### Evidence from RCTs

#### Evidence from RCTs: substance-induced psychosis in healthy individuals and patients with depression

For healthy individuals without personal or familial risk for psychosis, no long-lasting psychotic reactions are reported in experimental conditions, albeit important anxiety reactions linked to the ego-dissolution phenomena have been described [64–66]. Psychological support seems to be highly effective in mitigating such anxiety responses. One RCT administered psilocybin and LSD to healthy volunteers. Two volunteers presented a loss of their insight during the psychedelic session with paranoid thoughts (1.5 mcg/kg of LSD, and 114 mcg/kg of psilocybin) [67]. In another RCT, ten former morphine addicts received intra-muscular LSD (0.75–1.5 mcg/kg) or mescaline (2.5–5 mg/kg), without adverse reactions reported [68].

Eight RCTs administering MDMA to patients with PTSD and resistant PTSD found no long-lasting psychotic symptoms. However, up to 90% of participants experienced the emergence of dose-dependent anxiety (Table 1.5.2).

Moreover, we found eight RCTs administering doses ranging from 25(+12.5) mg to 125(+62.5) mg to PTSD patients (Table 1.5.2). Authors observed that a minority of patients experienced pronounced anxiety, correlating with dose [69], with anxiety reactions reported in up to 90% of cases at higher doses. Short-term and frequent insomnia reactions were also reported.

Except for some studies published before 1990, most presented a low risk of bias and a Level 1–2 OCEBM classification. Similarly, for DMT, no increase in psychotic reactions was found (Table 1.2.2).

For psilocybin, nine RCTs were identified with doses ranging from 3 to 50 mg/70 kg (Table 1.3.2), essentially for patients with depression and resistant depression (with no personal of familial history of psychosis). None of the recent studies published from 1999 to 2023 has reported enduring psychotic reactions, except for one study with a patient experiencing abnormal dream illusions [70], but without requiring antipsychotics. Nonetheless, a few authors note brief paranoia or heightened anxiety during DMT sessions, affecting fewer than 10% of patients [71, 72].

### Evidence from RCTs: use of psychedelics in patients with schizophrenia

One RCT conducted in 1966 was identified [73]. Patients with chronic schizophrenia using LSD mostly improved with euphoric reactions followed by improved social behavior. For three weeks, they received between 100 and 300 mg LSD daily, followed by 100 to 300 mg of thioridazine a few hours later. After that only thioridazine continued for an additional period of three weeks. Considering the lack of information on randomization and information on the placebo used, this study presented critical risk of bias and a Level 2 OCEBM classification.

### MRI-studies of psychedelic drug effects

We identified several MRI studies focusing on population of regular users of psychedelics, suggesting no specific harm of psychedelics on the brain. These findings might be of particular interest because they could provide potentially a putative therapeutic mechanism of how psychedelics might ameliorate negative symptoms in schizophrenia (Wolf et al. [8]). We detail these studies in the supplements (Supplementary Information 3).

### Hallucinogen persisting perception disorder

Occurrence of Hallucinogen Persisting Perception Disorder (HPPD) (not considered a primary psychedelic-induced psychosis) has also been studied. HPPD was not associated with exacerbation of psychotic symptoms in individuals with schizophrenia (Supplementary Information 4).

### Guidelines for safety and adverse events managements

We identified two guidelines on human hallucinogen research and on the abuse potential of medical psilocybin (Supplementary Table S7). We report in the supplement these resources, and several reviews, and cross-sectional study on selection of participants for psychedelic trials (Supplementary Table S7). In most studies, the assumption of contra-indications for inclusion of patients with schizophrenia, psychosis or bipolar disorder is mainly based on case reports.

### Meta-analysis of the incidence of psychedelic-induced psychosis

We retrieved seven studies that reported lifetime incidence of psychedelic-induced psychosis, in healthy individuals [51, 67, 74, 75], patients with depression [22], or patients with schizophrenia [13, 14].

Furthermore, 10 studies also reported the risk of psychedelic-induced psychosis according to the number of psychedelic sessions (Baker, [76]; S. Cohen [37]; Fink, [13]; Hoch et al. [14]; Leuner, [77]; Malleson, [78]; McGlothlin & Arnold, [51]; Novak [79]; Perez et al. [22]; Studerus et al. [75]) and five studies reported incidences of psychedelic-induced psychosis in population studies [50, 53, 54, 80, 81].

We detail characteristics of all retained study for the meta-analysis in the supplements (Supplementary Table S8).

### Meta-analysis of the incidence of psychedelic-induced psychosis according to the number of sessions, in healthy individuals, patients with depression, or patients with schizophrenia

A total of 7931 sessions of LSD and psilocybin were included from 10 low to high quality experimental studies, mostly involving LSD, published between 1960 and 2010: seven cohort studies (Baker [82]; S. Cohen [37]; Leuner [77]; Malleson [78]; McGlothlin & Arnold [51]; Novak [79]), one pool of eight high-quality RCTs in healthy individuals (*n* = 110) [75]; seven RCTs in patients with depression (*n* = 263) [22]; and two non-randomized controlled studies in patients with schizophrenia (*n* = 133) [13, 14]. One retrieved RCTs delivering LSD to patients with schizophrenia could not be included, as there were no clear cases or worsening for all 20 included patients in the intervention group [73].

The details of all retained studies in our meta-analysis is reported in the supplement (Supplementary Table S8).

When pooling all studies, the combined incidence of psychedelic-induced psychosis was 0.4% (95%CI 0.1–1), with notable heterogeneity (I^2^ = 75%; *n* = 7931) (Fig. 1A). The visual inspection of the funnel plot indicated that the observed heterogeneity was primarily attributed to the studies involving patients with schizophrenia (Supplementary Fig. S1).

We conducted a sensitivity analysis regarding this analysis, by including only the two studies with low risk of bias, that excluded patients with a history of psychosis (Studerus et al., 2010; Perez et al. [22]) (Supplementary Fig. S3). The combined incidence was however similar 0.4% (95%CI 0.1–1.6) in absence of heterogeneity (I^2^ = 0%; *n* = 490).

Furthermore, when excluding patients with psychiatric disorders, the incidence of psychedelic-induced psychosis in healthy participants dropped to 0.2% (95%CI 0.1–0.3; I^2^ = 0%; *n* = 8) (Fig. 1B). Moreover, the incidence of psychedelic-induced psychosis for patients with depression was not different from the incidence in healthy participants 0.4% (95%CI 0.1–2.8). When considering the subgroup of patients with schizophrenia, this incidence was higher 3.8% (95%CI 1.6–8.9; I^2^ = 0%; *n* = 2)(Fig. 1C), however, it is important to note that these studies were published more than half a century ago. These patients had from one to three sessions of LSD (10-120 µg oral LSD, and IV 0.5-10 µg/kg LSD), and in almost all cases no long-lasting psychotic symptoms were observed. One cross-sectional, web-based study was not included in our meta-analysis, considering the self-report nature of the data, in the absence of confirmed diagnoses (Evans et al. 2023). In this study, the incidence in unsupervised conditions of long-lasting psychotic symptoms induced by psychedelics was 5%.

### Lifetime occurrence of prolonged psychosis

**Fig. 2:**
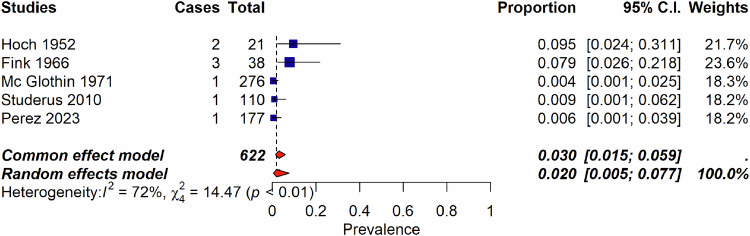
Lifetime occurrence of psychedelic-induced psychosis according to the number of participants affected.

**Fig. 3:**
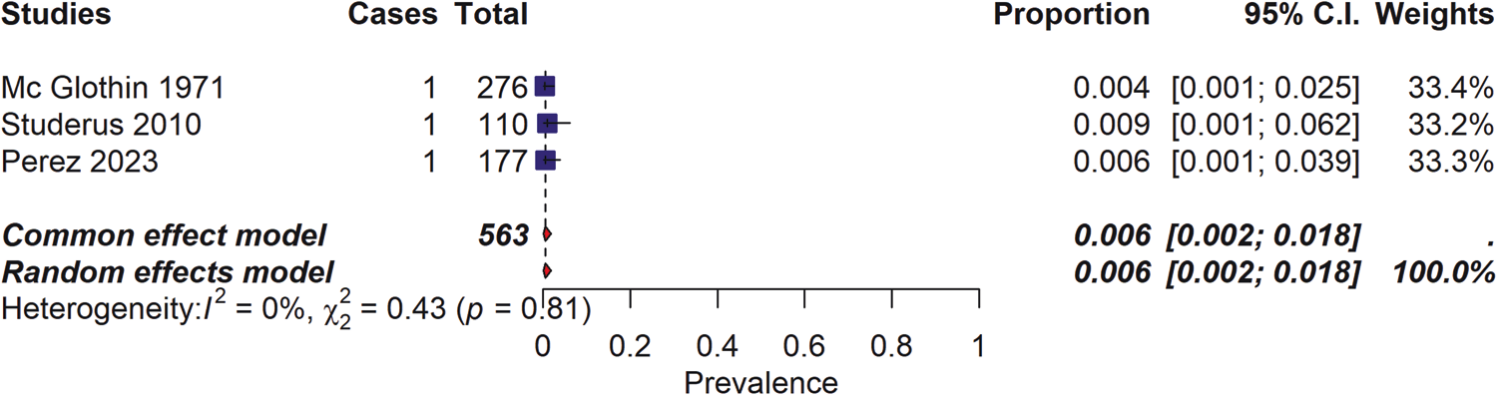
Incidence of psychedelic-induced psychosis in healthy individuals and patients with depression.

Lifetime occurrences of prolonged psychosis were documented in five studies (*n* = 622): one follow-up study involving healthy participants [51], two trials with patients with psychosis [13, 14], and two articles summarizing several recent clinical trials, including healthy individuals and patients with depression. The observed incidence of psychedelic-induced psychosis was 2% (95%CI 0.5–7.7; I^2^ = 0%) across a cohort of 622 patients (Fig. 2). With exclusion of the two lower quality studies including patients with schizophrenia (*n* = 563), the incidence dropped to 0.6% (95%CI 0.2–1.8; I^2^ = 0%) (Fig. 3). We conducted a sensitivity analysis, retaining two studies with low risk of bias (Studerus et al., 2010; Perez et al. [22]) (Supplementary Fig. S4), however results were unchanged 0.7% (95%CI 0.2–2.8; I^2^ = 0%).

### Incidence rate of psychedelic-induced psychosis in population studies

**Fig. 4:**
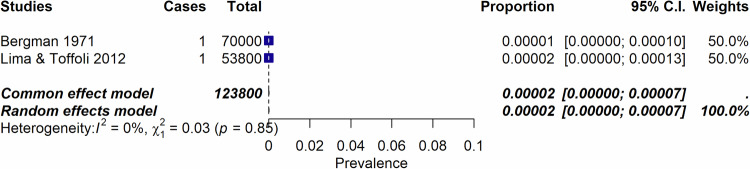
Lifetime occurrence of psychedelic-induced psychosis in indigenous populations.

Two studies reported the rate of psychedelic-induced psychosis in population studies (*n* = 123,800) [53, 54]. One study reported the use of ayahuasca and the other one of peyote in ritual ceremonies. Pooling those two studies of psychedelics users in indigenous populations, we obtained an incidence of 0.002% (95%CI 0-0.006; I^2^ = 0%) (Fig. 4).

### Rate of conversion from substance-induced psychotic disorder to schizophrenia

**Fig. 5:**
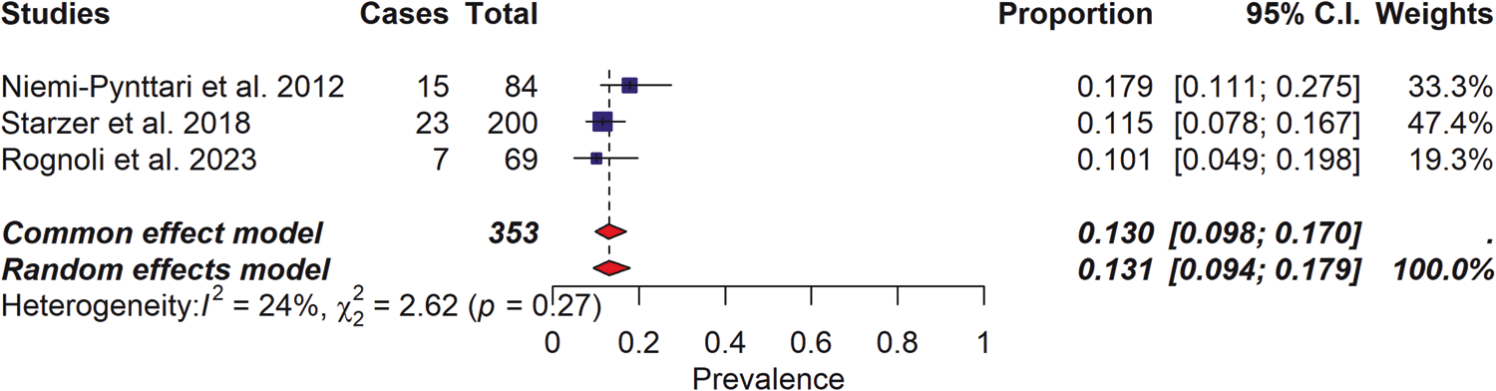
Incidence of conversion from substance-induced psychotic disorder due to psychedelic use (main drug of use) to schizophrenia.

Three studies reported the rate of conversion from psychedelic-induced psychotic disorder (SIPD) to schizophrenia in three population follow-up studies of patients that used hallucinogens as their main drugs of use (*n* = 353) [50, 80, 81]. The incidence of conversion from SIPD to schizophrenia was 13.1% (95%CI 9.4-17.9) over a mean period of 10.5 years pin the presence of a low heterogeneity (I^2^ = 24%) (Fig. 5).

## Discussion

The present study is the first comprehensive systematic review and meta-analysis investigating the incidence of psychedelic-induced psychosis in the general population, indigenous population, and individuals with mental disorders, including schizophrenia. This study provides a wide-ranging overview and hierarchy of evidence to address the question of the risk of psychedelic-induced psychosis across different populations and psychedelics.

Our findings from population-based studies indicate a very small incidence of psychedelic-induced psychosis in both the general population and indigenous population that frequently use psychedelics. Recent RCTs involving patients with depression reported that such enduring side effects occur in less than 2% of cases. However, it is important to note that these trials excluded patients at risk for psychosis and therefore do not directly inform on the risk of triggering psychotic symptoms in these excluded and potentially vulnerable subgroups of patients.

In contrast, instances of psychedelic-induced psychotic reactions predominantly occurred in individuals who obtained psychedelics from illicit sources or used new psychoactive substances [83]. Notably, several UCTs further report a low incidence of psychedelic-induced psychosis in patients with schizophrenia or with autism spectrum disorders (less than 4%). Notably, these studies were conducted during the 1970s and presented with high risks of bias, which limits their credibility. Moreover, we observed an incidence rate of 13% of patients transitioning from psychedelic-induced psychosis to schizophrenia. This pooled rate was obtained from just three good quality studies, which contrasts with the higher rate of 26% reported by Murrie and colleagues [20], who included also one PCP study in their analysis.

In summary, our incidence findings suggest that most of the evidence around the risk of psychosis associated with serotoninergic psychedelics is of low quality, yet that use of serotonergic psychedelics rarely induced psychosis in the general population -when excluding patients with psychotic features- while the risk of transition to schizophrenia is considerable in those who develop psychedelic-induced psychosis. It is important to highlight that a significant portion of the available data derives from lower-quality studies and definitive conclusions cannot be drawn from the existing evidence.

### Phenomenology of serotoninergic psychedelics trip

According to Hoch et al. there seems to be a low incidence of auditory hallucinations but a significant occurrence of visual hallucinations in patients with schizophrenia experimenting with mescaline and LSD [14]. Langs & Barr noted that LSD primarily induces visual hallucinations, whereas patients with schizophrenia display more delusional thinking [84]. Wießner and colleagues suggest the connection between the psychosis model and therapeutic model could rest in mystical experiences [85].

Among reviewed works, Strassman’s 1984 article on adverse reactions to psychedelic drugs distinguishes various reactions—from acute, time-limited panic responses during administration, to brief and transient psychotic fluctuations as well as psychoses lasting days, to recurrent flashbacks and persistent undifferentiated psychotic and treatment-resistant cases [7]. Strassman proposed that the development of LSD-induced psychosis is multifactorial, involving factors such as family history, personality traits, and exposure to multiple drugs [7]. In identified reviews, Paparelli and colleagues suggested that stimulants and THC are more likely to induce paranoid beliefs, particularly with repeated use, while LSD is more closely associated with visual hallucinations [86].

More recently, Orsolini and colleagues advise against ayahuasca use in patients with bipolar disorder or a psychotic disorder due to an elevated risk of manic episodes (based on two case reports) and psychotic onset [87]. A web-based survey on experience with ‘magic-mushroom’ consumption also found that a third of patients with self-reported bipolar disorder diagnosis presented hypomanic symptoms in the week following drug ingestion [88], raising more questions on the risk of manic switch than of exacerbation of psychotic symptoms. This finding is strengthened with a recent longitudinal study by Honk and colleagues reporting from 1.3 to 6.5% and 2 to 12% of psychotic symptoms in individuals that had a lifetime use of psychedelics, and a psychedelic use in the last 2 months, respectively. We were not able to include this study in our meta-analysis considering how results were reported, nevertheless, the authors conclude that among individuals with a personal or family history of bipolar disorders, psychedelic use was associated with an increase in psychotic symptoms, and on the contrary were decreased in individuals with a personal (but not family) history of psychotic disorders. Authors propose that while psychedelic use attenuates (or does not affect) the risk for psychosis in individuals with a personal (or family) history of psychotic disorders, it increases the risk for mania with psychotic features in individuals with a personal or family history of bipolar disorder [89].

### Dose-response findings

The determination of the ‘optimal safe dose’ is a critical consideration in addressing the issue of psychedelic-induced psychosis. While each patient may have their own personalized optimal dosage, a recent study identified near-maximum effective doses of psilocybin, referred to as ED95, for depression scores. The ED95 doses were 8.92, 24.68, and 36.08 mg/70 kg for patients with secondary depression, primary depression, and both subgroups, respectively [22].

Moreover, we identified several studies that reported that individuals with schizophrenia often exhibit a subpar response to typical moderate dosages of psilocybin that are well-tolerated by healthy subjects. The question of whether exceeding the conventional dosage is warranted, particularly for patients burdened with severe negative symptoms and limited positive symptoms, originates from the findings of Perez and colleagues, who found that patients with treatment-resistant depression require higher doses of psilocybin (36.08 mg/kg). Conversely, patients prone to significant positive symptoms may benefit from commencing their treatment with lower doses to mitigate the risk of long-lasting psychotic reactions. In addition, dose-dependent risk of anxiety symptoms have been reported up to 90% of individuals with anxiety disorders at doses of 125 mg MDMA [90]. Given that anxiety disorders are a frequently co-occurring comorbidity in patients with psychosis, dose-dependent risk should be considered for multiple symptom dimension beyond acute exacerbation of positive symptoms.

Identifying the “optimal safe dose” for add-on psychedelic treatment in patients with psychosis also concerns a critical consideration of the potential interacting effects with concomitant antipsychotic medication. Many second-generation antipsychotics have high affinity to 5HT2A receptors and act as antagonists, therefore maybe attenuating the therapeutic effects of psychedelic drugs. In contrast, antipsychotics with high affinity to D2 receptors, such as haloperidol -at a given dose-, might have no effect or potential even worsen some psychedelic effects (Vollenweider et al. [9]; Vollenweider & Kometer [91]). Thus, the effects of simultaneous treatment with antipsychotics and psychedelics needs further investigation.

In addition, other elements, such as the purity of the substance, or the setting might also be relevant to consider. Many authors propose that psychedelic-induced psychosis appears to be more prevalent in recreational or uncontrolled settings involving black-market substances. For instance, synthetic cathinones are marketed as cheap substitutes for other stimulants, such as amphetamines and cocaine, as is the case for products sold as Molly.

### Neurobiological mechanism of potential therapeutic effects

Individualized doses of psilocybin could be a therapeutic option for patients presenting with treatment-resistant/predominant depressive symptoms or negative symptoms [18, 92].

Consequently, the enhancement of neuroplasticity as well as the psychedelic experience could both carry therapeutic effects [93]. Timmermann et al. [94] suggest that the long-term benefits of these psychedelics could be due to enhanced neuroplasticity [94]. Neuroplasticity might be mainly promoted by activation of intracellular 5-HT2A receptors in animal studies [95, 96] and/or, by direct binding of BDNF receptor Trkb that may allow for a relatively widespread rewiring of neuronal networks [97]. In a recent publication, Heresco-Levy & Lerer proposed that intricate serotonergic-glutamatergic interactions, including ionotropic glutamate receptors, tropomyosin receptor kinase B (TrkB), and the mammalian target of rapamycin (mTOR), play a pivotal role in neuroplasticity induced by serotonergic psychedelics [98]. Integrating these lines of research and their own preliminary data ([98], the authors hypothesize that the administration of a psychedelic and an NMDAR modulator simultaneously may enhance the therapeutic efficacy of each treatment and facilitate dose adjustments and enhanced safety [98].

In addition, to enhanced neuroplasticity, it has been proposed that the profound ‘mystical’ experiences induced by psychedelics could be a key factor in their immediate antidepressant effects [99, 100]. However, whether these experiences are related to increased neuroplasticity in humans remains an open question.

### Therapeutic potential of serotoninergic psychedelics in patients with schizophrenia

In the reviewed trials with healthy participants and non-psychotic disorders, individuals with a personal or familial history of psychotic features were systematically excluded. This exclusion criterion poses a challenge in interpreting results, which cannot be applied to patients exhibiting a clinical or familial risk for psychosis. Consequently, the same cautious approach should be extended to populations at risk for substance-induced psychosis stemming from various substances, warranting their exclusion from clinical trials involving psychedelics. However, cohort studies specifically investigating the safety and efficacy of these substances in such populations under strictly controlled conditions would be of high interest.

Based on the few existing low-quality studies, there is no clear evidence for high incidence rates of psychedelic-induced psychosis in antipsychotic treated patients. Further high-quality studies are required to either confirm or refute the observed low incidence rates in the present study. Furthermore, the current findings may prompt a re-evaluation of the existing contraindications, which currently bar patients with schizophrenia who may be eligible for participation in research studies testing psychedelic-assisted therapy. In this regard, individuals with chronic and stable schizophrenia, particularly those experiencing predominant depressive and/or negative symptoms and continuous antipsychotic medication, may qualify for psychedelic-assisted therapy.

In fact, an ascending-dose tolerability study involving MDMA for patients with schizophrenia has already started (NCT05770375). Future trials are already starting for other specific populations, such as individuals with autism spectrum disorders (NCT05651126) or comorbid personality disorders (NCT05399498). Nevertheless, ethical considerations underscore the imperative that individuals within the schizophrenia-spectrum disorder, possessing a history of aggressive or suicidal behavior during psychotic episodes, should not undergo exposure to this therapeutic intervention.

### Limitations

This work has several limitations. While we included a significant number of studies, there was considerable variation in their type and quality. Overall quality of studies was low and only few studies (*n* = 9) could be included in the meta-analysis, hence the presented findings should be interpreted with caution. Moreover, studies primarily focused on different types of serotonergic psychedelics. Diagnoses across the 70-year span show evolving standards, affecting methodological consistency (e.g., study methods, settings, psychological support). Most studies were classified as having a critical risk of bias according to the ROBINS scale, with an OCEBM level of 2–3. Current RCTs that present a low risk of bias propose that psychedelic-induced psychosis is exceptional in controlled settings, including patients not presenting with psychotic symptoms or risk. Although most results were coherent and obtained low to moderate heterogeneity, several factors could potentially impact our results. Notably, population characteristics before the onset of any psychiatric issues are often not reported, as is the cumulative exposure to drugs and the overall quality of the drugs considered. For instance, illicitly manufactured LSD may be contaminated with substances that have atropine-like effects, potentially influencing the occurrence of psychotic symptoms. Additionally, the definition of ‘psychedelics-induced psychosis’ lacks firm establishment, with current authors often using SIPD DSM-5 criteria, requiring a temporal association between drug exposure and persistent psychotic symptoms. Furthermore, another important issue is the effective blinding in RCTs involving psychedelics due to the inherent alterations in consciousness that these compounds induce synergistic effects between the current psychological therapy and expectancy effects of the psychedelic trips are also difficult to control [101]. One solution to this limitation could be to include active placebos or active comparator study designs in future studies [102].

## Conclusion

The incidence of serotonergic psychedelics-induced psychosis was relatively low in the general population, but a considerably increased risk of transition to schizophrenia emerged after psychedelic-induced psychosis. Although the evidence base is currently comprised of only low-quality studies, the current findings warrant further studies to clarify whether the categorical exclusion of individuals living with schizophrenia is indeed necessary for studies involving the administration of psychedelic substances. Moreover, studies developing and validating prediction models of psychedelic-induced psychosis, and transition from psychedelic-induced psychosis to schizophrenia are needed. In conclusion, high-quality research is required to clarify the risk-benefit ratio of psychedelic treatments in individuals with schizophrenia and a conservative approach is recommended until further data is available.

## Supplementary information

Supplementary Materials

## Data Availability

All data generated or analyzed during this study are included in this published article (and its Supplementary Data files). The data and the analysis R code that generated the results and figures is available online with the metafor package.

## Code availability

Code used for these analyses are available from the corresponding author upon reasonable request.

## References

[1] Solmi M, Chen C, Daure C, Buot A, Ljuslin M, Verroust V, et al. A century of research on psychedelics: A scientometric analysis on trends and knowledge maps of hallucinogens, entactogens, entheogens and dissociative drugs. Eur Neuropsychopharmacol J Eur Coll Neuropsychopharmacol. 2022;64:44–60. 10.1016/j.euroneuro.2022.09.004 36191546

[2] Goodwin GM, Aaronson ST, Alvarez O, Arden PC, Baker A, Bennett JC, et al. Single-dose psilocybin for a treatment-resistant episode of major depression. N Engl J Med. 2022;387:1637–48. 36322843 10.1056/NEJMoa2206443

[3] van der Meer PB, Fuentes JJ, Kaptein AA, Schoones JW, de Waal MM, Goudriaan AE, et al. Therapeutic effect of psilocybin in addiction: a systematic review. Front Psychiatry. 2023;14:1134454. 36846225 10.3389/fpsyt.2023.1134454 PMC9947277

[4] Feulner L, Sermchaiwong T, Rodland N, Galarneau D. Efficacy and safety of psychedelics in treating anxiety disorders. Ochsner J. 2023;23:315–28. 38143548 10.31486/toj.23.0076 PMC10741816

[5] Khan AJ, Bradley E, O’Donovan A, Woolley J. Psilocybin for trauma-related disorders. Curr Top Behav Neurosci. 2022;56:319–32. 35711024 10.1007/7854_2022_366

[6] Aaronson ST, van der Vaart A, Miller T, LaPratt J, Swartz K, Shoultz A, et al. Single-dose synthetic psilocybin with psychotherapy for treatment-resistant bipolar type II major depressive episodes: a nonrandomized controlled trial. JAMA Psychiatry. 2023. 10.1001/jamapsychiatry.2023.4685. 10.1001/jamapsychiatry.2023.4685 PMC10701666 38055270

[7] Strassman RJ. Adverse reactions to psychedelic drugs. A review of the literature. J Nerv Ment Dis. 1984;172:577–95. 6384428 10.1097/00005053-198410000-00001

[8] Wolf G, Singh S, Blakolmer K, Lerer L, Lifschytz T, Heresco-Levy U, et al. Could psychedelic drugs have a role in the treatment of schizophrenia? Rationale and strategy for safe implementation. Mol Psychiatry. 2023;28:44–58. 36280752 10.1038/s41380-022-01832-z

[9] Vollenweider F, Vollenweider-Scherpenhuyzen M, Bäbler A, Vogel H, Hell D. Psilocybin induces schizophrenia-like psychosis in humans via a serotonin-2 agonist action. Neuroreport. 1999;9:3897–902. 10.1097/00001756-199812010-00024 9875725

[10] Sabé M, Zhao N, Kaiser S. A systematic review and meta-analysis of the prevalence of cocaine-induced psychosis in cocaine users. Prog Neuro-Psychopharmacology Biol Psychiatry. 2021;109:110263. 10.1016/j.pnpbp.2021.110263 33524454

[11] Solmi M, De Toffol M, Kim JY, Choi MJ, Stubbs B, Thompson T, et al. Balancing risks and benefits of cannabis use: umbrella review of meta-analyses of randomised controlled trials and observational studies. BMJ. 2023;382:e072348. 37648266 10.1136/bmj-2022-072348 PMC10466434

[12] Bowers MB Jr, Mazure CM, Nelson JC, Jatlow PI. Psychotogenic drug use and neuroleptic response. Schizophr Bull. 1990;16:81–5. 1970670 10.1093/schbul/16.1.81

[13] Fink M, Simeon J, Haque W, Itil T. Prolonged adverse reactions to LSD in psychotic subjects. Arch Gen Psychiatry. 1966;15:450–4. 5926592 10.1001/archpsyc.1966.01730170002002

[14] Hoch PH, Cattell JP, Pennes HH. Effects of Mescaline and Lysergic Acid (d-LSD-25). Am J Psychiatry. 1952;108:579–84. 14903183 10.1176/ajp.108.8.579

[15] Kramer M. Cross-national study of diagnosis of the mental disorders: origin of the problem. Am J Psychiatry. 1969;125:1–11. 10.1176/ajp.125.10s.1 4953061

[16] Helfer B, Samara MT, Huhn M, Klupp E, Leucht C, Zhu Y, et al. Efficacy and safety of antidepressants added to antipsychotics for schizophrenia: a systematic review and meta-analysis. Am J Psychiatry. 2016;173:876–86. 27282362 10.1176/appi.ajp.2016.15081035

[17] Ly C, Greb AC, Cameron LP, Wong JM, Barragan EV, Wilson PC, et al. Psychedelics promote structural and functional neural plasticity. Cell Rep. 2018;23:3170–82. 29898390 10.1016/j.celrep.2018.05.022 PMC6082376

[18] Arnovitz MD, Spitzberg AJ, Davani AJ, Vadhan NP, Holland J, Kane JM, et al. MDMA for the treatment of negative symptoms in schizophrenia. J Clin Med. 2022;11:3255. 10.3390/jcm11123255 PMC9225098 35743326

[19] Cohen S. LSD: the varieties of psychotic experience. J Psychoactive Drugs. 1985;17:291–6. 4087079 10.1080/02791072.1985.10524333

[20] Murrie B, Lappin J, Large M, Sara G. Transition of substance-induced, brief, and atypical psychoses to schizophrenia: a systematic review and meta-analysis. Schizophr Bull. 2020;46:505–16. 31618428 10.1093/schbul/sbz102 PMC7147575

[21] Seeman P, Guan H-C. Phencyclidine and glutamate agonist LY379268 stimulate dopamine D2High receptors: D2 basis for schizophrenia. Synapse. 2008;62:819–28. 18720422 10.1002/syn.20561

[22] Perez N, Langlest F, Mallet L, De Pieri M, Sentissi O, Thorens G, et al. Psilocybin-assisted therapy for depression: a systematic review and dose-response meta-analysis of human studies. Eur Neuropsychopharmacol. 2023;76:61–76. 37557019 10.1016/j.euroneuro.2023.07.011

[23] Mustafa NS, Bakar NHA, Mohamad N, Adnan LHM, Fauzi NFAM, Thoarlim A, et al. MDMA and the brain: a short review on the role of neurotransmitters in neurotoxicity. Basic Clin Neurosci. 2020;11:381–8. 33613876 10.32598/bcn.9.10.485 PMC7878040

[24] Liechti ME, Vollenweider FX. Which neuroreceptors mediate the subjective effects of MDMA in humans? A summary of mechanistic studies. Hum Psychopharmacol. 2001;16:589–98. 12404538 10.1002/hup.348

[25] Mitchell JM, Ot’alora G M, van der Kolk B, Shannon S, Bogenschutz M, Gelfand Y, et al. MDMA-assisted therapy for moderate to severe PTSD: a randomized, placebo-controlled phase 3 trial. Nat Med. 2023;29:2473–80. 37709999 10.1038/s41591-023-02565-4 PMC10579091

[26] Belbasis L, Bellou V, Ioannidis JPA. Conducting umbrella reviews. BMJ Med. 2022;1:e000071. 36936579 10.1136/bmjmed-2021-000071 PMC9951359

[27] Fiorentini A, Cantù F, Crisanti C, Cereda G, Oldani L, Brambilla P. Substance-induced psychoses: an updated literature review. Front Psychiatry. 2021;12:694863. 35002789 10.3389/fpsyt.2021.694863 PMC8732862

[28] Shea BJ, Reeves BC, Wells G, Thuku M, Hamel C, Moran J, et al. AMSTAR 2: a critical appraisal tool for systematic reviews that include randomised or non-randomised studies of healthcare interventions, or both. BMJ. 2017;358:j4008. 28935701 10.1136/bmj.j4008 PMC5833365

[29] Baethge C, Goldbeck-Wood S, Mertens S. SANRA—a scale for the quality assessment of narrative review articles. Res Integr Peer Rev. 2019;4:5. 30962953 10.1186/s41073-019-0064-8 PMC6434870

[30] Sterne JAC, Savović J, Page MJ, Elbers RG, Blencowe NS, Boutron I, et al. RoB 2: a revised tool for assessing risk of bias in randomised trials. BMJ. 2019;366:l4898. 31462531 10.1136/bmj.l4898

[31] Sterne JAC, Hernán MA, Reeves BC, Savović J, Berkman ND, Viswanathan M, et al. ROBINS-I: a tool for assessing risk of bias in non-randomised studies of interventions. BMJ. 2016;355:i4919. 27733354 10.1136/bmj.i4919 PMC5062054

[32] OCEBM. The Oxford Levels of Evidence 2. OCEBM Levels Evid Work Gr. 2011. 2011.

[33] DerSimonian R, Kacker R. Random-effects model for meta-analysis of clinical trials: an update. Contemp Clin Trials. 2007;28:105–14. 16807131 10.1016/j.cct.2006.04.004

[34] Higgins JPT, Thompson SG, Deeks JJ, Altman DG. Measuring inconsistency in meta-analyses. BMJ. 2003;327:557–60. 12958120 10.1136/bmj.327.7414.557 PMC192859

[35] Viechtbauer W. Conducting Meta-Analyses in R with the metafor Package. J Stat Softw. 2010;36:1–48.

[36] Barker TH, Migliavaca CB, Stein C, Colpani V, Falavigna M, Aromataris E, et al. Conducting proportional meta-analysis in different types of systematic reviews: a guide for synthesisers of evidence. BMC Med Res Methodol. 2021;21:189. 34544368 10.1186/s12874-021-01381-z PMC8451728

[37] Cohen S. Lysergic acid diethylamide: side effects and complications. J Nerv Ment Dis. 1960;130:30–40. 13811003 10.1097/00005053-196001000-00005

[38] Bercel NA, Travis LEEE, Olinger LB, Dreikurs E, Polos MG. Model psychoses induced by LSD-25 in normals: I. Psychophysiological investigations, with special reference to the mechanism of the paranoid reaction. AMA Arch Neurol Psychiatry. 1956;75:588–611. 13325989 10.1001/archneurpsyc.1956.02330240026003

[39] Gillin JC, Kaplan J, Stillman R, Wyatt RJ. The psychedelic model of schizophrenia: the case of N,N -dimethyltryptamine. Am J Psychiatry. 1976;133:203–8. 1062171 10.1176/ajp.133.2.203

[40] Rümmele W, Gnirss F. Untersuchungen mit Psilocybin, einer psychotropen Subtanz aus Psilocybe Mexicana. Schweiz Arch Neurol Psychiatr. 1961;87:365–85. 13744546

[41] Anderson BT, Danforth A, Daroff PR, Stauffer C, Ekman E, Agin-Liebes G, et al. Psilocybin-assisted group therapy for demoralized older long-term AIDS survivor men: An open-label safety and feasibility pilot study. EClinicalMedicine. 2020;27:100538. 33150319 10.1016/j.eclinm.2020.100538 PMC7599297

[42] Stoll WA. Lysergsäure-Diäthylamid, ein Phantastikum aus der Mutterkorngruppe. Schweiz Arch Neurol Psychiatr. 1947;60:279–323.

[43] Condrau G. Klinische erfahrungen an geisteskranken mit lysergsäure-diäthylamid. Acta Psychiatr Scand. 1949;24:9–32.

[44] Belsanti R. Modificazioni neuro-psico-biochimiche indotte dalla dietilamide dell’acido lisergico in schizofrenici e frenastenici. Acta Neurol (Napoli). 1952;7:25.

[45] Anastasopoulos G, Photiades H. Effects of LSD-25 on relatives of schizophrenic patients. J Ment Sci. 1962;108:95–8. 13861108 10.1192/bjp.108.452.95

[46] Forrer GR, Goldner RD. Experimental physiological studies with lysergic acid diethylamide (LSD-25). AMA Arch Neurol Psychiatry. 1951;65:581–8. 14818479 10.1001/archneurpsyc.1951.02320050038004

[47] Liddell DW, Weil-Malherbe H. The effects of methedrine and of lysergic acid diethylamide on mental processes and on the blood andrenaline level. J Neurol Neurosurg Psychiatry. 1953;16:7–13. 13023434 10.1136/jnnp.16.1.7 PMC503108

[48] Bender L, Faretra G, Cobrinik L. LSD and UML treatment of hospitalized disturbed children. Recent Adv Biol Psychiatry. 1963;5:84–92.

[49] Bowers MBJ. Psychoses precipitated by psychotomimetic drugs. A follow-up study. Arch Gen Psychiatry. 1977;34:832–5. 195549 10.1001/archpsyc.1977.01770190094009

[50] Niemi-Pynttäri JA, Sund R, Putkonen H, Vorma H, Wahlbeck K, Pirkola SP. Substance-induced psychoses converting into schizophrenia. J Clin Psychiatry. 2013;74:e94–e99. 23419236 10.4088/JCP.12m07822

[51] McGlothlin WH, Arnold DO. LSD revisited: a ten-year follow-up of medical LSD use. Arch Gen Psychiatry. 1971;24:35–49. 5538851 10.1001/archpsyc.1971.01750070037005

[52] Vardy MM, Kay SR. LSD psychosis or LSD-induced schizophrenia?: a multimethod inquiry. Arch Gen Psychiatry. 1983;40:877–83. 6870484 10.1001/archpsyc.1983.01790070067008

[53] Lima FAS, Tófoli LF, Labate B, Jungaberle H. An epidemiological surveillance system by the UDV: mental health recommendations concerning the religous use of hoasca 2011.

[54] Bergman RL. Navajo peyote use: Its apparent safety. Am J Psychiatry. 1971;128:695–9. 5147724 10.1176/ajp.128.6.695

[55] Dos Santos R, Bouso JC, Hallak J. Ayahuasca, dimethyltryptamine, and psychosis: A systematic review of human studies. Ther Adv Psychopharmacol. 2017;7:1–17. 28540034 10.1177/2045125316689030 PMC5433617

[56] Landabaso MA, Iraurgi I, Jiménez-Lerma JM, Calle R, Sanz J, Gutiérrez-Fraile M. Ecstasy-induced psychotic disorder: six-month follow-up study. Eur Addict Res. 2002;8:133–40. 12065963 10.1159/000059383

[57] Krebs TS, Johansen P-Ø. Psychedelics and mental health: a population study. PLoS One. 2013;8:e63972. 23976938 10.1371/journal.pone.0063972 PMC3747247

[58] Baylé FJ, Misdrahi D, Llorca PM, Lançon C, Olivier V, Quintin P, et al. [Acute schizophrenia concept and definition: investigation of a French psychiatrist population]. Encephale. 2005;31:10–7. 15971635 10.1016/s0013-7006(05)82367-x

[59] Johansen P-Ø, Krebs T. Psychedelics not linked to mental health problems or suicidal behavior: A population study. J Psychopharmacol. 2015;29. 10.1177/0269881114568039 25744618

[60] Ungerleider JT, Fisher DD, Fuller M, Caldwell A. The ‘bad trip’–the etiology of the adverse LSD reaction. Am J Psychiatry. 1968;124:1483–90. 4384739 10.1176/ajp.124.11.1483

[61] Hays P, Tilley JR. The differences between LSD psychosis and schizophrenia. Can Psychiatr Assoc J. 1973;18:331–3. 4780752 10.1177/070674377301800413

[62] Rugani F, Bacciardi S, Rovai L, Pacini M, Maremmani AGI, Deltito J, et al. Symptomatological features of patients with and without Ecstasy use during their first psychotic episode. Int J Environ Res Public Health. 2012;9:2283–92. 22851941 10.3390/ijerph9072283 PMC3407902

[63] Sloane B, Doust LW. Psychophysiological investigations in experimental psychoses: results of the exhibition of d-lysergic acid diethylamide to psychiatric patients. J Ment Sci. 1954;100:129–44. 13152528 10.1192/bjp.100.418.129

[64] Schmid Y, Enzler F, Gasser P, Grouzmann E, Preller K, Vollenweider F, et al. Acute effects of lysergic acid diethylamide in healthy subjects. Biol Psychiatry. 2015;78:544–53. 10.1016/j.biopsych.2014.11.015 25575620

[65] Preller K, Burt J, Ji JL, Schleifer C, Adkinson B, Staempfli P, et al. Changes in global and thalamic brain connectivity in LSD-induced altered states of consciousness are attributable to the 5-HT2A receptor. Elife. 2018;7. 10.7554/eLife.35082. 10.7554/eLife.35082 PMC6202055 30355445

[66] Wiessner I, Falchi M, Palhano-Fontes F, Feilding A, Ribeiro S, Tófoli L. LSD, madness and healing: Mystical experiences as possible link between psychosis model and therapy model. Psychol Med. 2021;53:1–15. 34253268 10.1017/S0033291721002531

[67] Isbell H. Comparison of the reactions induced by psilocybin and LSD-25 in man. Psychopharmacologia. 1959;1:29–38. 14405870 10.1007/BF00408109

[68] Wolbach AB, Miner EJ, Isbell H. Comparison of psilocin with psilocybin, mescaline and LSD-25. Psychopharmacologia. 1962;3:219–23. 14007905 10.1007/BF00412109

[69] Mithoefer MC, Wagner MT, Mithoefer AT, Jerome L, Doblin R. The safety and efficacy of {+/-}3,4-methylenedioxymethamphetamine-assisted psychotherapy in subjects with chronic, treatment-resistant posttraumatic stress disorder: the first randomized controlled pilot study. J Psychopharmacol. 2011;25:439–52. 20643699 10.1177/0269881110378371 PMC3122379

[70] Carhart-Harris R, Giribaldi B, Watts R, Baker-Jones M, Murphy-Beiner A, Murphy R, et al. Trial of psilocybin versus escitalopram for depression. N Engl J Med. 2021;384:1402–11. 33852780 10.1056/NEJMoa2032994

[71] Davis AK, Barrett FS, May DG, Cosimano MP, Sepeda ND, Johnson MW, et al. Effects of psilocybin-assisted therapy on major depressive disorder: a randomized clinical trial. JAMA Psychiatry. 2021;78:481–9. 33146667 10.1001/jamapsychiatry.2020.3285 PMC7643046

[72] Hasler F, Grimberg U, Benz MA, Huber T, Vollenweider FX. Acute psychological and physiological effects of psilocybin in healthy humans: a double-blind, placebo-controlled dose-effect study. Psychopharmacology (Berl). 2004;172:145–56. 14615876 10.1007/s00213-003-1640-6

[73] Shirvaikar RV, Kelkar YW. Therapeutic trial of lysergic Acid diethylamide (LSD) and thioridazine in chronic schizophrenia. Neurol India. 1966;14:97–101. 5330728

[74] Carstairs SD, Cantrell FL. Peyote and mescaline exposures: a 12-year review of a statewide poison center database. Clin Toxicol (Phila). 2010;48:350–3. 20170392 10.3109/15563650903586745

[75] Studerus E, Kometer M, Hasler F, Vollenweider FX. Acute, subacute and long-term subjective effects of psilocybin in healthy humans: a pooled analysis of experimental studies. J Psychopharmacol. 2011;25:1434–52. 20855349 10.1177/0269881110382466

[76] Baker E LSD psychotherapy LSD psycho-exploration: three reports. In: Bobbs-Merrill, editor. use LSD Psychother. Alcohol., Indianapolis: Abramson HA; 1967. p. 191–207.

[77] Leuner H Present state of psycholytic therapy and its possibilities. In: AH, Abramson, editors. 2nd Int. Conf. Use LSD Psychother. Alcohol., Amityville: The Bobbs-Merrill Co. Inc. 1965.

[78] Malleson N. Acute Adverse Reactions to Lsd in Clinical and Experimental use in the United Kingdom. Br J Psychiatry. 1971;118:229–30. 4995932 10.1192/bjp.118.543.229

[79] Novak SJ. LSD before Leary. Sidney Cohen’s critique of 1950s psychedelic drug research. Isis; an Int Rev Devoted to Hist Sci Its Cult Influ. 1997;88:87–110. 10.1086/383628 9154737

[80] Starzer MSK, Nordentoft M, Hjorthøj C. Rates and predictors of conversion to schizophrenia or bipolar disorder following substance-induced psychosis. Am J Psychiatry. 2018;175:343–50. 29179576 10.1176/appi.ajp.2017.17020223

[81] Rognli EB, Heiberg IH, Jacobsen BK, Høye A, Bramness JG. Transition from substance-induced psychosis to schizophrenia spectrum disorder or bipolar disorder. Am J Psychiatry. 2023;180:437–44. 37132221 10.1176/appi.ajp.22010076

[82] Baker EF. The use of Lysergic acid diethylamide (LSD) in psychotherapy. Can Med Assoc J. 1964;91:1200–2. 14226093 PMC1928491

[83] Graddy R, Buresh ME, Rastegar DA. New and Emerging Illicit Psychoactive Substances. Med Clin North Am. 2018;102:697–714. 29933824 10.1016/j.mcna.2018.02.010

[84] Langs RJ, Barr HL Lysergic acid diethylamide (lsd-25) and schizophrenic reactions. J Nerv Ment Dis. 1968;147. 10.1097/00005053-196808000-00008 5677324

[85] Wießner I, Falchi M, Palhano-Fontes F, Feilding A, Ribeiro S, Tófoli LF. LSD, madness and healing: Mystical experiences as possible link between psychosis model and therapy model. Psychol Med. 2023;53:1151–65. 34253268 10.1017/S0033291721002531

[86] Paparelli A, Di Forti M, Morrison PD, Murray RM. Drug-induced psychosis: how to avoid star gazing in schizophrenia research by looking at more obvious sources of light. Front Behav Neurosci. 2011;5:1. 21267359 10.3389/fnbeh.2011.00001 PMC3024828

[87] Orsolini L, Chiappini S, Papanti D, Latini R, Volpe U, Fornaro M, et al. How does ayahuasca work from a psychiatric perspective? Pros and cons of the entheogenic therapy. Hum Psychopharmacol. 2020;35:e2728. 32220028 10.1002/hup.2728

[88] Morton E, Sakai K, Ashtari A, Pleet M, Michalak EE, Woolley J. Risks and benefits of psilocybin use in people with bipolar disorder: An international web-based survey on experiences of ‘magic mushroom’ consumption. J Psychopharmacol. 2023;37:49–60. 36515370 10.1177/02698811221131997 PMC9834328

[89] Honk L, Stenfors CUD, Goldberg SB, Hendricks PS, Osika W, Dourron HM, et al. Longitudinal associations between psychedelic use and psychotic symptoms in the United States and United Kingdom. J Affect Disord. 2024. 10.1016/j.jad.2024.01.197. 10.1016/j.jad.2024.01.197 PMC10922895 38280572

[90] Mithoefer MC, Feduccia AA, Jerome L, Mithoefer A, Wagner M, Walsh Z, et al. MDMA-assisted psychotherapy for treatment of PTSD: study design and rationale for phase 3 trials based on pooled analysis of six phase 2 randomized controlled trials. Psychopharmacology (Berl). 2019;236:2735–45. 31065731 10.1007/s00213-019-05249-5 PMC6695343

[91] Vollenweider FX, Kometer M. The neurobiology of psychedelic drugs: implications for the treatment of mood disorders. Nat Rev Neurosci. 2010;11:642–51. 20717121 10.1038/nrn2884

[92] Hirschfeld T, Schmidt TT. Dose-response relationships of psilocybin-induced subjective experiences in humans. J Psychopharmacol. 2021;35:384–97. 33663259 10.1177/0269881121992676 PMC8058832

[93] Gattuso JJ, Perkins D, Ruffell S, Lawrence AJ, Hoyer D, Jacobson LH, et al. Default Mode Network Modulation by Psychedelics: A Systematic Review. Int J Neuropsychopharmacol. 2023;26:155–88. 36272145 10.1093/ijnp/pyac074 PMC10032309

[94] Timmermann C, Bauer PR, Gosseries O, Vanhaudenhuyse A, Vollenweider F, Laureys S, et al. A neurophenomenological approach to non-ordinary states of consciousness: hypnosis, meditation, and psychedelics. Trends Cogn Sci. 2023;27:139–59. 36566091 10.1016/j.tics.2022.11.006

[95] Vargas MV, Dunlap LE, Dong C, Carter SJ, Tombari RJ, Jami SA, et al. Psychedelics promote neuroplasticity through the activation of intracellular 5-HT2A receptors. Science. 2023;379:700–6. 36795823 10.1126/science.adf0435 PMC10108900

[96] de Vos CMH, Mason NL, Kuypers KPC. Psychedelics and neuroplasticity: a systematic review unraveling the biological underpinnings of psychedelics. Front Psychiatry. 2021;12:72606. 10.3389/fpsyt.2021.724606 PMC8461007 34566723

[97] Moliner R, Girych M, Brunello CA, Kovaleva V, Biojone C, Enkavi G, et al. Psychedelics promote plasticity by directly binding to BDNF receptor TrkB. Nat Neurosci. 2023;26:1032–41. 37280397 10.1038/s41593-023-01316-5 PMC10244169

[98] Heresco-Levy U, Lerer B. Synergistic psychedelic - NMDAR modulator treatment for neuropsychiatric disorders. Mol Psychiatry. 2024;29:146–52. 37945694 10.1038/s41380-023-02312-8

[99] Ko K, Knight G, Rucker JJ, Cleare AJ. Psychedelics, mystical experience, and therapeutic efficacy: a systematic review. Front Psychiatry. 2022;13:917199. 35923458 10.3389/fpsyt.2022.917199 PMC9340494

[100] Winkelman MJ. The mechanisms of psychedelic visionary experiences: hypotheses from evolutionary psychology. Front Neurosci. 2017;11:539. 10.3389/fnins.2017.00539 PMC5625021 29033783

[101] Dworkin RH, McDermott MP, Nayak SM, Strain EC. Psychedelics and psychotherapy: is the whole greater than the sum of its parts? Clin Pharmacol Ther. 2023;114:1166–9. 37795632 10.1002/cpt.3050

[102] Wen A, Singhal N, Jones BDM, Zeifman RJ, Mehta S, Shenasa MA, et al. A systematic review of study design and placebo controls in psychedelic research. Psychedelic Med. 2023;2:15–24. 10.1089/psymed.2023.0028 PMC11658677 40051762

[103] Busch A, Johnson W. L.S.D. 25 as an aid in psychotherapy; preliminary report of a new drug. Dis Nerv Syst. 1950;11:241–3. 14793387

[104] De Giacomo U. La catatonie toxique expérimentale. Acta Neurol (Napoli). 1951;7:5–10.

[105] Mayer-Gross W. Experimental psychoses and other mental abnormalities produced by drugs. Br Med J. 1951;2:317–21. 14858846 10.1136/bmj.2.4727.317 PMC2069705

[106] Katzenelbogen S, Fang AD. Narcosynthesis effects of sodium amytal, methedrine and L.S.D-25. Dis Nerv Syst. 1953;14:85–8. 13033687

[107] Cholden LS, Kurland A, Savage C. Clinical reactions and tolerance to LSD in chronic schizophrenia. J Nerv Ment Dis. 1955;122. 10.1097/00005053-195509000-00001 13295823

[108] Abramson HA. The use of LSD in psychotherapy: transactions of a conference on D-lysergic acid diethylamide (LSD-25). April 22, 23 and 24, 1959, Princeton, NJ: Josiah Macy, Jr. Foundation; 1960.

[109] Freedman AM, Ebin EVAV, Wilson EA. Autistic schizophrenic children: an experiment in the use of D-Lysergic acid diethylamide (LSD-25). Arch Gen Psychiatry. 1962;6:203–13. 13894863 10.1001/archpsyc.1962.01710210019003

[110] Fischer G, Castile D. An investigation to determine the therapeutic effectiveness of LSD-25 and psilocybin on hospitalized severely emotionally disturbed children. Purdue University Archives and Special Collections; 1963.

[111] Bender L, Goldschmidt L, Sankar DVS, Freedman AM. Treatment of autistic schizophrenic children with LSD-25 and UML-491 BT—recent advances in biological psychiatry: Volume IV: The Proceedings of the Sixteenth Annual Convention and Scientific Program of the Society of Biological Psychiatry, Atlantic City. In: Wortis J, editor. Springer US; 1962. p. 170–9. 10.1007/978-1-4684-8306-2_17.

[112] Bender L. D-lysergic acid in the treatment of the biological features of childhood schizophrenia. Dis Nerv Syst. 1966;7:43–6. 5965284

[113] Blumenfield M, Glickman L. Ten months experience with LSD users admitted to county psychiatric receiving hospital. N Y State J Med. 1967;67:1849–53. 5232667

[114] Dewhurst K, Hatrick JA. Differential diagnosis and treatment of lysergic acid diethylamide induced psychosis. Practitioner. 1972;209:327–32. 5081313

[115] Roy A. LSD and onset of schizophrenia. Can J Psychiatry. 1981;26:64–5. 7471035 10.1177/070674378102600113

[116] Smart RG, Storm T, Baker EF, Solursh L. A controlled study of lysergide in the treatment of alcoholism. 1. The effects on drinking behavior. Q J Stud Alcohol. 1966;27:469–82. 5970697

[117] Lev-Ran S, Feingold D, Frenkel A, Lerner AG. Clinical characteristics of individuals with schizophrenia and hallucinogen persisting perception disorder: a preliminary investigation. J Dual Diagn. 2014;10:79–83. 25392249 10.1080/15504263.2014.906155

[118] Lev-Ran S, Feingold D, Rudinski D, Katz S, Arturo LG. Schizophrenia and hallucinogen persisting perception disorder: a clinical investigation. Am J Addict. 2015;24:197–9. 25808913 10.1111/ajad.12204

[119] Tomsovic M, Edwards RV. Lysergide treatment of schizophrenic and nonschizophrenic alcoholics: a controlled evaluation. Q J Stud Alcohol. 1970;31:932–49. 5490826

[120] Gasser P, et al. Safety and efficacy of lysergic acid diethylamide-assisted psychotherapy for anxiety associated with life-threatening diseases. J Nerv Ment Dis. 2014;202:513–20. 24594678 10.1097/NMD.0000000000000113 PMC4086777

[121] Schmid Y, et al. Acute effects of lysergic acid diethylamide in healthy subjects. Biol Psychiatry. 2015;78:544–53. 25575620 10.1016/j.biopsych.2014.11.015

[122] Jiménez-Garrido DF, et al. Effects of ayahuasca on mental health and quality of life in naïve users: a longitudinal and cross-sectional study combination. Sci Rep. 2020;10:4075. 32139811 10.1038/s41598-020-61169-x PMC7057990

[123] Bickel P, Dittrich A, Schopf J. Effekte von N,N-dimethyltryptamin (DMT) auf psychotizismus-tests. Pharmacopsychiatry. 1977;10:10–14. 10.1055/s-0028-1094512 329291

[124] Bouso JC, et al. Long-term use of psychedelic drugs is associated with differences in brain structure and personality in humans. Eur Neuropsychopharmacol. 2015;25:483–92. 10.1016/j.euroneuro.2015.01.008 25637267

[125] Strassman RJ, Qualls CR, Uhlenhuth EH, Kellner R. Dose-response study of N,N-dimethyltryptamine in humans. II. Subjective effects and preliminary results of a new rating scale. Arch Gen Psychiatry. 1994;51:98–108. 8297217 10.1001/archpsyc.1994.03950020022002

[126] Dos Santos RG, et al. Ayahuasca improves self-perception of speech performance in subjects with social anxiety disorder: a pilot, proof-of-concept, randomized, placebo-controlled trial. J Clin Psychopharmacol. 2021;41:540–50. 34166299 10.1097/JCP.0000000000001428

[127] vollenweider, F. X. et al. Positron emission tomography and fluorodeoxyglucose sturies of metabolic hyperfrontality and psychopathology in the psilocybin model of psychosis. Neuropsychopharmacol. 1997;16;357–72. 10.1016/S0893-133X(96)00246-1 9109107

[128] Garcia-Romeu A, Griffiths RR, Johnson MW. Psilocybin-occasioned mystical experiences in the treatment of tobacco addiction. Curr Drug Abus Rev. 2014;7:157–64. 10.2174/1874473708666150107121331 PMC4342293 25563443

[129] Grob CS, et al. Pilot study of psilocybin treatment for anxiety in patients with advanced-stage cancer. Arch Gen Psychiatry. 2011;68:71–8. 20819978 10.1001/archgenpsychiatry.2010.116

[130] Griffiths RR, et al. Psilocybin produces substantial and sustained decreases in depression and anxiety in patients with life-threatening cancer: a randomized double-blind trial. J Psychopharmacol. 2016;30:1181–97. 27909165 10.1177/0269881116675513 PMC5367557

[131] Ross S, et al. Rapid and sustained symptom reduction following psilocybin treatment for anxiety and depression in patients with life-threatening cancer: a randomized controlled trial. J Psychopharmacol. 2016;30:1165–80. 27909164 10.1177/0269881116675512 PMC5367551

[132] Bogenschutz MP, et al. Percentage of heavy drinking days following psilocybin-assisted psychotherapy vs placebo in the treatment of adult patients with alcohol use disorder: a randomized clinical trial. JAMA Psychiatry. 2022;79:953–62. 36001306 10.1001/jamapsychiatry.2022.2096 PMC9403854

[133] von Rotz R, et al. Single-dose psilocybin-assisted therapy in major depressive disorder: A placebo-controlled, double-blind, randomised clinical trial. EClinicalMedicine. 2023;56:101809. 36636296 10.1016/j.eclinm.2022.101809 PMC9830149

[134] Bouso JC, Doblin R, Farré M, Alcázar MA, Gómez-Jarabo G. MDMA-assisted psychotherapy using low doses in a small sample of women with chronic posttraumatic stress disorder. J Psychoact Drugs. 2008;40:225–36. 10.1080/02791072.2008.10400637 19004414

[135] Oehen P, Traber R, Widmer V, Schnyder U. A randomized, controlled pilot study of MDMA (± 3,4-Methylenedioxymethamphetamine)-assisted psychotherapy for treatment of resistant, chronic Post-Traumatic Stress Disorder (PTSD). J Psychopharmacol. 2013;27:40–52. 23118021 10.1177/0269881112464827

[136] Danforth AL, et al. Reduction in social anxiety after MDMA-assisted psychotherapy with autistic adults: a randomized, double-blind, placebo-controlled pilot study. Psychopharmacol. (Berl). 2018;235:3137–48. 10.1007/s00213-018-5010-9 PMC6208958 30196397

[137] Ot’alora GM, et al. 3,4-Methylenedioxymethamphetamine-assisted psychotherapy for treatment of chronic posttraumatic stress disorder: a randomized phase 2 controlled trial. J Psychopharmacol. 2018;32:1295–307. 30371148 10.1177/0269881118806297 PMC6247454

[138] Jerome L, et al. Long-term follow-up outcomes of MDMA-assisted psychotherapy for treatment of PTSD: a longitudinal pooled analysis of six phase 2 trials. Psychopharmacol. (Berl). 2020;237:2485–97. 10.1007/s00213-020-05548-2 PMC7351848 32500209

[139] Mitchell JM, et al. MDMA-assisted therapy for severe PTSD: a randomized, double-blind, placebo-controlled phase 3 study. Nat Med. 2021;27:1025–33. 33972795 10.1038/s41591-021-01336-3 PMC8205851

[140] Ponte L, et al. Sleep quality improvements after MDMA-assisted psychotherapy for the treatment of posttraumatic stress disorder. J Trauma Stress. 2021;34:851–63. 34114250 10.1002/jts.22696 PMC8453707

[141] Brewerton TD, Gavidia I, Suro G, Perlman MM. Eating disorder patients with and without PTSD treated in residential care: discharge and 6-month follow-up results. J Eat Disord. 2023;11:48. 36973828 10.1186/s40337-023-00773-4 PMC10044735

[142] Nicholas CR, et al. The effects of MDMA-assisted therapy on alcohol and substance use in a phase 3 trial for treatment of severe PTSD. Drug Alcohol Depend. 2022;233:109356. 35286849 10.1016/j.drugalcdep.2022.109356 PMC9750500

[143] Pacey I. Randomized, double-blind, controlled of MDMA-assisted psychotherapy in 12 subjects with PTSD. NCT01958593. Lykos Therapeutics. 2017. https://clinicaltrials.gov/study/NCT01958593.

[144] Wolfson PE, et al. MDMA-assisted psychotherapy for treatment of anxiety and other psychological distress related to life-threatening illnesses: a randomized pilot study. Sci Rep. 2020;10:20442. 33235285 10.1038/s41598-020-75706-1 PMC7686344

[145] Hollister LEOE, Shelton J, Krieger G. A controlled comparison of lysergic acid diethylamide (LSD) and dextroamphetamine in alcoholics. Am J Psychiatry. 1969;125:1352–57. 4886242 10.1176/ajp.125.10.1352

[146] Breakey WR, Goodell H, Lorenz PC, McHugh PR. Hallucinogenic drugs as precipitants of schizophrenia. Psychol Med. 1974;4:255–61. 4427973 10.1017/s0033291700042938

[147] Boutros NN, Bowers MBJ. Chronic substance-induced psychotic disorders: state of the literature. J Neuropsychiatry Clin Neurosci. 1996;8:262–9. 8854296 10.1176/jnp.8.3.262

[148] Shoval G, et al. Substance use, suicidality, and adolescent-onset schizophrenia: an Israeli 10-year retrospective study. J Child Adolesc Psychopharmacol. 2006;16:767–75. 17201620 10.1089/cap.2006.16.767

[149] Hendricks PS, Clark CB, Johnson MW, Fontaine KR, Cropsey KL. Hallucinogen use predicts reduced recidivism among substance-involved offenders under community corrections supervision. J Psychopharmacol. 2014;28:62–6. 24399338 10.1177/0269881113513851

[150] Hendricks PS, Thorne CB, Clark CB, Coombs DW, Johnson MW. Classic psychedelic use is associated with reduced psychological distress and suicidality in the United States adult population. J Psychopharmacol. 2015;29:280–8. 25586402 10.1177/0269881114565653

[151] Vallersnes OM, et al. Psychosis associated with acute recreational drug toxicity: a European case series. BMC Psychiatry. 2016;16:293. 27538886 10.1186/s12888-016-1002-7 PMC4990880

[152] Evans J, et al. Extended difficulties following the use of psychedelic drugs: a mixed methods study. PLoS ONE. 2023;18:e0293349. 37874826 10.1371/journal.pone.0293349 PMC10597511

[153] Simonsson O, et al. Longitudinal associations between psychedelic use and unusual visual experiences in the United States and the United Kingdom. J. Psychopharmacol. 2023. 10.1177/02698811231218931. 10.1177/02698811231218931 PMC10851627 38140891

[154] Smart RG, Bateman K. Unfavourable reactions to LSD: a review and analysis of the available case reports. Can Med Assoc J. 1967;97:1214–21. 4862277 PMC1923615

[155] Leuner H. Present state of psycholytic therapy and its possibilities. In: Abramson AH, editor. 2nd International Conference on the Use of LSD in Psychotherapy and Alcoholism. Amityville, N.Y.: The Bobbs-Merrill Co. Inc.; 1965.

[156] Panhuysen LH. Undesirable side effects of LSD administration. Ned Tijdschr Geneeskd. 1970;114:723–7. 4910547

[157] De Gregorio D, Comai S, Posa L, Gobbi G. d-lysergic acid diethylamide (LSD) as a model of psychosis: mechanism of action and pharmacology. Int J Mol Sci. 2016;17. 10.3390/ijms17111953 PMC5133947 27886063

[158] Jacob MS, Presti DE. Endogenous psychoactive tryptamines reconsidered: an anxiolytic role for dimethyltryptamine. Med Hypotheses. 2005;64:930–7. 15780487 10.1016/j.mehy.2004.11.005

[159] Grammenos D, Barker SA. On the transmethylation hypothesis: stress, N,N-dimethyltryptamine, and positive symptoms of psychosis. J Neural Transm. 2015;122:733–9. 25362533 10.1007/s00702-014-1329-5

[160] Gable RS. Risk assessment of ritual use of oral dimethyltryptamine (DMT) and harmala alkaloids. Addiction. 2007;102:24–34. 17207120 10.1111/j.1360-0443.2006.01652.x

[161] Skryabin VY, Vinnikova M, Nenastieva A, Alekseyuk V. Hallucinogen persisting perception disorder: A literature review and three case reports. J Addict Dis. 2018;37:268–78. 31613183 10.1080/10550887.2019.1673655

[162] McGuire P. Long term psychiatric and cognitive effects of MDMA use. Toxicol Lett. 2000;112–113:153–6. 10720725 10.1016/s0378-4274(99)00219-2

[163] Soar K, Turner JJD, Parrott AC. Psychiatric disorders in Ecstasy (MDMA) users: a literature review focusing on personal predisposition and drug history. Hum Psychopharmacol. 2001;16:641–5. 12404545 10.1002/hup.350

[164] Smith KW, Sicignano DJ, Hernandez AV, White CM. MDMA-assisted psychotherapy for treatment of posttraumatic stress disorder: a systematic review with meta-analysis. J Clin Pharmacol. 2022;62:463–71. 34708874 10.1002/jcph.1995

[165] Mogar RE, Aldrich RW. The use of psychedelic agents with autistic schizophrenic children. Behav Neuropsychiatry. 1969;1:44–50. 5374546

[166] Glass GS. Psychedelic drugs, stress, and the ego. J Nerv Ment Dis. 1973;156:232–41. 4708883 10.1097/00005053-197304000-00003

[167] McCabe OL. Psychedelic drug crises: toxicity and therapeutics. J Psychedelic Drugs. 1977;9:107–21.

[168] Hermele M, Ran Y, Lee PA, Wen X-G. Properties of an algebraic spin liquid on the kagome lattice. Phys Rev B. 2008;77:224413.

[169] Trope A, et al. Psychedelic-assisted group therapy: a systematic review. J Psychoact Drugs. 2019;51:174–88. 10.1080/02791072.2019.1593559 PMC6650145 30950777

[170] Lerner AG, et al. Flashback and Hallucinogen Persisting Perception Disorder: clinical aspects and pharmacological treatment approach. Isr J Psychiatry Relat Sci. 2002;39:92–9. 12227234

[171] Litjens RPW, Brunt TM, Alderliefste G-J, Westerink RHS. Hallucinogen persisting perception disorder and the serotonergic system: a comprehensive review including new MDMA-related clinical cases. Eur Neuropsychopharmacol. 2014;24:1309–23. 24933532 10.1016/j.euroneuro.2014.05.008

[172] Halpern JH, Pope HGJ. Hallucinogen persisting perception disorder: what do we know after 50 years. Drug Alcohol Depend. 2003;69:109–19. 12609692 10.1016/s0376-8716(02)00306-x

[173] Orsolini L, et al. The ‘Endless Trip’ among the NPS users: psychopathology and psychopharmacology in the hallucinogen-persisting perception disorder. a systematic review. Front Psychiatry. 2017;8:240. 29209235 10.3389/fpsyt.2017.00240 PMC5701998

[174] Martinotti G, et al. Hallucinogen persisting perception disorder: etiology, clinical features, and therapeutic perspectives. Brain Sci. 2018;8. 10.3390/brainsci8030047 PMC5870365 29547576

[175] Doyle MA, et al. Hallucinogen persisting perceptual disorder: a scoping review covering frequency, risk factors, prevention, and treatment. Expert Opin Drug Saf. 2022;21:733–43. 35426769 10.1080/14740338.2022.2063273

