# Supplementary material for: Reconsidering evidence for psychedelic-induced psychosis: an overview of reviews, a systematic review, and meta-analysis of human studies
Source: Mol Psychiatry. 2024 Nov 27;30(3):1223–55. doi: 10.1038/s41380-024-02800-5 (PMC11835720; doi:10.1038/s41380-024-02800-5)
Supplement: Supplementary file 1 — Supplementary Materials [file 41380_2024_2800_MOESM1_ESM.docx]

**Supplementary material**

**Reconsidering evidence for psychedelic-induced psychosis: An overview of reviews, a systematic review, and meta-analysis of human studies**

Michel Sabé, Adi Sulstarova, Alban Glangetas, Marco De Pieri, Luc Mallet, Logos Curtis, Hélène Richard-Lepouriel, Louise Penzenstadler, Federico Seragnoli, Gabriel Thorens, Daniele Zullino, Katrin Preller, Kerem Böge, Stefan Leucht, Christoph U. Correll, Marco Solmi, Stefan Kaiser, Matthias Kirschner.

**Supplementary Information 1.** PRISMA 2020 Checklist and PRISMA 2020 Abstracts checklist

**Supplementary Information 2.** Diagnostic criteria of substance-induced psychosis according to the DSM-5

**Supplementary Information 3.** MRI-studies of psychedelic drug effects

**Supplementary Information 4.** Hallucinogen Persisting Perception Disorder

**Supplementary Table S1.** Summary of retrieved studies on long-lasting psychotic reactions phenomena

**Supplementary Table S2.** List of excluded studies with reasons

**Supplementary Table S3.** Assessment of non-systematics review using the SANRA scale

**Supplementary Table S4.** Assessment of systematic reviews using the AMSTAR-2 Criteria

**Supplementary Table S5.** Assessment of the quality non-randomized studies using ROBINS-I tool

**Supplementary Table S6.** Cochrane collaboration risk of bias assessment for included RCTs.

**Supplementary Table S7.** Studies, reviews, and guidelines on prevention of potential adverse events and hallucinogen-induced psychosis

**Supplementary Table S8.** Detail of included studies in our meta-analysis

**Supplementary Fig S1.** Systematic review PRISMA flowchart

**Supplementary Fig S2.** Funnel plot and Trim and fill method

**Supplementary Fig S3.** Sensitivity analysis of psychedelic-induced psychosis according to the number of sessions across healthy individuals, patients with depression, and patients with schizophrenia

**Supplementary Fig S4**. Sensitivity analysis for the incidence of psychedelic-induced psychosis in healthy individuals and patients with depression.

**Supplementary Information 5.** References of all included studies

**Supplementary Information 1.** PRISMA 2020 Checklist and PRISMA 2020 Abstracts checklist

| **Section and Topic** | **Item #** | **PRISMA 2020 Checklist** | **Location where item is reported** |
| --- | --- | --- | --- |
| **TITLE** | | |  |
| Title | 1 | Identify the report as a systematic review. | Title |
| **ABSTRACT** | | |  |
| Abstract | 2 | See the PRISMA 2020 for Abstracts checklist. | See below |
| **INTRODUCTION** | | |  |
| Rationale | 3 | Describe the rationale for the review in the context of existing knowledge. | p.4 |
| Objectives | 4 | Provide an explicit statement of the objective(s) or question(s) the review addresses. | p.6 |
| **METHODS** | | |  |
| Eligibility criteria | 5 | Specify the inclusion and exclusion criteria for the review and how studies were grouped for the syntheses. | p.6, 7 |
| Information sources | 6 | Specify all databases, registers, websites, organisations, reference lists and other sources searched or consulted to identify studies. Specify the date when each source was last searched or consulted. | p.6 |
| Search strategy | 7 | Present the full search strategies for all databases, registers and websites, including any filters and limits used. | p.6 |
| Selection process | 8 | Specify the methods used to decide whether a study met the inclusion criteria of the review, including how many reviewers screened each record and each report retrieved, whether they worked independently, and if applicable, details of automation tools used in the process. | p.6, 7 |
| Data collection process | 9 | Specify the methods used to collect data from reports, including how many reviewers collected data from each report, whether they worked independently, any processes for obtaining or confirming data from study investigators, and if applicable, details of automation tools used in the process. | p.6, 7 |
| Data items | 10a | List and define all outcomes for which data were sought. Specify whether all results that were compatible with each outcome domain in each study were sought (e.g. for all measures, time points, analyses), and if not, the methods used to decide which results to collect. | p.6, 7, 8 |
|  | 10b | List and define all other variables for which data were sought (e.g. participant and intervention characteristics, funding sources). Describe any assumptions made about any missing or unclear information. | p.6, 7 |
| Study risk of bias assessment | 11 | Specify the methods used to assess risk of bias in the included studies, including details of the tool(s) used, how many reviewers assessed each study and whether they worked independently, and if applicable, details of automation tools used in the process. | p.7 |
| Effect measures | 12 | Specify for each outcome the effect measure(s) (e.g. risk ratio, mean difference) used in the synthesis or presentation of results. | p.8 |
| Synthesis methods | 13a | Describe the processes used to decide which studies were eligible for each synthesis (e.g. tabulating the study intervention characteristics and comparing against the planned groups for each synthesis (item #5)). | p.6, 7 |
|  | 13b | Describe any methods required to prepare the data for presentation or synthesis, such as handling of missing summary statistics, or data conversions. | p.7 |
|  | 13c | Describe any methods used to tabulate or visually display results of individual studies and syntheses. | p.7, 8 |
|  | 13d | Describe any methods used to synthesize results and provide a rationale for the choice(s). If meta-analysis was performed, describe the model(s), method(s) to identify the presence and extent of statistical heterogeneity, and software package(s) used. | p.7 |
|  | 13e | Describe any methods used to explore possible causes of heterogeneity among study results (e.g. subgroup analysis, meta-regression). | p.6, 7 |
|  | 13f | Describe any sensitivity analyses conducted to assess robustness of the synthesized results. | p.6, 7 |
| Reporting bias assessment | 14 | Describe any methods used to assess risk of bias due to missing results in a synthesis (arising from reporting biases). | p.7 |
| Certainty assessment | 15 | Describe any methods used to assess certainty (or confidence) in the body of evidence for an outcome. | p.7 |
| **RESULTS** | | |  |
| Study selection | 16a | Describe the results of the search and selection process, from the number of records identified in the search to the number of studies included in the review, ideally using a flow diagram. | p.8  Fig S1 |
|  | 16b | Cite studies that might appear to meet the inclusion criteria, but which were excluded, and explain why they were excluded. | Info 5 |
| Study characteristics | 17 | Cite each included study and present its characteristics. | Table 1, 2 |
| Risk of bias in studies | 18 | Present assessments of risk of bias for each included study. | Table 1, 2, Table S1 |
| Results of individual studies | 19 | For all outcomes, present, for each study: (a) summary statistics for each group (where appropriate) and (b) an effect estimate and its precision (e.g. confidence/credible interval), ideally using structured tables or plots. | Fig 2-5 |
| Results of syntheses | 20a | For each synthesis, briefly summarise the characteristics and risk of bias among contributing studies. | Table 1 ,2  Table S3-6 |
|  | 20b | Present results of all statistical syntheses conducted. If meta-analysis was done, present for each the summary estimate and its precision (e.g. confidence/credible interval) and measures of statistical heterogeneity. If comparing groups, describe the direction of the effect. | P.13-16 |
|  | 20c | Present results of all investigations of possible causes of heterogeneity among study results. | Table 1, 2 |
|  | 20d | Present results of all sensitivity analyses conducted to assess the robustness of the synthesized results. | n.r. |
| Reporting biases | 21 | Present assessments of risk of bias due to missing results (arising from reporting biases) for each synthesis assessed. | Table S3-6 |
| Certainty of evidence | 22 | Present assessments of certainty (or confidence) in the body of evidence for each outcome assessed. | Table 1, 2  Table S3-6 |
| **DISCUSSION** | | |  |
| Discussion | 23a | Provide a general interpretation of the results in the context of other evidence. | p.16-20 |
|  | 23b | Discuss any limitations of the evidence included in the review. | p.16-20 |
|  | 23c | Discuss any limitations of the review processes used. | p.21 |
|  | 23d | Discuss implications of the results for practice, policy, and future research. | p.20-22 |
| **OTHER INFORMATION** | | |  |
| Registration and protocol | 24a | Provide registration information for the review, including register name and registration number, or state that the review was not registered. | p.6 |
|  | 24b | Indicate where the review protocol can be accessed, or state that a protocol was not prepared. | p.6 |
|  | 24c | Describe and explain any amendments to information provided at registration or in the protocol. | p.6 |
| Support | 25 | Describe sources of financial or non-financial support for the review, and the role of the funders or sponsors in the review. | p.23 |
| Competing interests | 26 | Declare any competing interests of review authors. | p.23 |
| Availability of data, code and other materials | 27 | Report which of the following are publicly available and where they can be found: template data collection forms; data extracted from included studies; data used for all analyses; analytic code; any other materials used in the review. | p.23 |

*From:*  Page MJ, McKenzie JE, Bossuyt PM, Boutron I, Hoffmann TC, Mulrow CD, et al. The PRISMA 2020 statement: an updated guideline for reporting systematic reviews. BMJ 2021;372:n71. doi:

| **Section and Topic** | **Item #** | **PRISMA 2020 Abstracts checklist** | **Reported (Yes/No)** |
| --- | --- | --- | --- |
| **TITLE** | | |  |
| Title | 1 | Identify the report as a systematic review. | Yes |
| **BACKGROUND** | | |  |
| Objectives | 2 | Provide an explicit statement of the main objective(s) or question(s) the review addresses. | Yes |
| **METHODS** | | |  |
| Eligibility criteria | 3 | Specify the inclusion and exclusion criteria for the review. | Yes |
| Information sources | 4 | Specify the information sources (e.g. databases, registers) used to identify studies and the date when each was last searched. | Yes |
| Risk of bias | 5 | Specify the methods used to assess risk of bias in the included studies. | Yes |
| Synthesis of results | 6 | Specify the methods used to present and synthesise results. | Yes |
| **RESULTS** | | |  |
| Included studies | 7 | Give the total number of included studies and participants and summarise relevant characteristics of studies. | Yes |
| Synthesis of results | 8 | Present results for main outcomes, preferably indicating the number of included studies and participants for each. If meta-analysis was done, report the summary estimate and confidence/credible interval. If comparing groups, indicate the direction of the effect (i.e. which group is favoured). | Yes |
| **DISCUSSION** | | |  |
| Limitations of evidence | 9 | Provide a brief summary of the limitations of the evidence included in the review (e.g. study risk of bias, inconsistency and imprecision). | Yes |
| Interpretation | 10 | Provide a general interpretation of the results and important implications. | Yes |
| **OTHER** | | |  |
| Funding | 11 | Specify the primary source of funding for the review. | No |
| Registration | 12 | Provide the register name and registration number. | Yes |

**Supplementary Information 2.** Diagnostic criteria of substance-induced psychosis according to the DSM-5

| **Disorder** | **Criteria** |
| --- | --- |
| Substance-induced psychosis | A. Presence of one or both of the following symptoms: •Delusions •Hallucinations B. There is evidence from the history, physical examination, or laboratory findings that either (1) or (2): •The symptoms in Criterion A developed during, or within a month of, substance intoxication or withdrawal •Medication used is etiologically related to the disturbance C. The disturbance is not more accounted for by a psychotic disorder that is not substance-induced. D. The disturbance does not occur exclusively during delirium. E. The disturbance causes clinically significant distress or impairment in social, occupational, or other important areas of functioning. |
| Primary psychotic diseases | This group includes: •Schizophrenia •Other psychotic diseases •Schizotypal personality disorder  All the previous conditions must have one or more symptoms of the following: •Delusions •Hallucinations •Disorganized speech •Disorganized behavior •Negative symptoms |
| Psychotic illness with comorbid substance use | At least, one of the criteria defining a psychotic disease and all the criteria of a substance use disorder must be present: •A pattern of use that results in marked distress and/or impairment, with two or more of the following symptoms for 12 months. •Using the substance in larger amounts or over a longer period of time than intended •Unsuccessful attempts or persistent desire to reduce its use •Excessive time spent on obtaining, using, and/or recovering from the effects of the substance •A pervasive craving for the substance •Significant interference with roles at work, school, or home •Continued use despite recurrent social or interpersonal consequences •Reducing or giving up important activities due to the substance use •Substance use in situations in which it may be physically hazardous •Substance use despite recurrent or persistent physical or psychological consequences •Tolerance of the substance •Withdrawal from the substance |

**Source:** American Psychiatric Association. Diagnostic and Statistical Manual of Mental Disorders: DSM-5, Fifth edit. Washington, DC (2013). 10.1176/appi.books.9780890425596

**Supplementary Information 3.** MRI-studies of psychedelic drug effects

Bouso and colleagues conducted a case-control MRI study among a small sample of regular ayahuasca users (25 sessions/year), revealing that consistent psychedelic drug use could potentially induce structural changes in brain areas, such as the cortical thickness of the posterior cingulate cortex (PCC), implicated in attention, self-referential thinking, and inner reflection ^1^. PET studies in healthy subjects ingesting psilocybin (Vollenweider et al., 1997, 1999) suggested that psilocybin decreased [11C]raclopride binding in the caudate nucleus and putamen, reflecting heightened striatal dopamine levels. The authors posed the question of whether such heightened striatal dopamine activity after psylocibin use might exacerbate positive symptoms in patients with schizophrenia. While this hypothesis is plausible from the current understanding of imbalanced striatal dopamine transmission in psychosis, it would have to be further confirmed by empirical data. Such finding further nourished the rationale of giving to patients with schizophrenia a concomitant antipsychotic medication (D2 blocker) while potentially benefiting from psychedelic-therapy. In addition to heightened striatal dopamine activity, this study also revealed a hyperfrontal metabolic pattern of regional cerebral glucose metabolism that contrasts with the hypofrontality reported in patients with chronic schizophrenia (Vollenweider et al., 1997).

These findings might be of particular interest because they could provide potentially a putative therapeutic mechanism of how psychedelics might ameliorate negative symptoms in schizophrenia ^4^.

**Supplementary Information 4.** Hallucinogen Persisting Perception Disorder

Two clinical studies on Hallucinogen Persisting Perception Disorder (HPPD) were selected (Lev-ran 2014; Lev-Ran 2015). These retrieved articles found no association between HPPD and the risk of enduring psychotic symptoms. Lev-Ran and colleagues discovered that in patients with schizophrenia who developed HPPD after LSD use, their negative symptom severity and general psychopathology scores were lower compared to schizophrenia patients with no prior psychedelic use, despite enduring perceptual disturbances.

Narrative reviews indicated HPPD prevalence as extremely rare, primarily linked to LSD and MDMA/Ecstasy use ^5–8^. Four systematic reviews on HPPD phenomena were identified. Authors contend that the term 'flashbacks' lacks clear definition, and limited information is available in most studies to assess whether cases meet DSM-IV criteria for HPPD ^9–12^.

Simonsson and colleagues proposed a longitudinal study including 9732 individuals. A total of 7667 patients completed the follow-up survey. Among the 100 individuals that reported psychedelic use during the 2-month study period, psychedelic-use was associated with greater increases in unusual visual experiences ^13^.

Of importance, all authors conclude that HPPD is not part of psychedelic-induced psychosis and is not associated with an increased prevalence of such side-effect.

**Supplementary Table S1.** Summary of retrieved studies on long-lasting psychotic reactions phenomena

| **Type of drug** | **Type of studies** | | | | | | |
| --- | --- | --- | --- | --- | --- | --- | --- |
|  | **Sytematic review^a^** | **Review** | **RCT** | **CC** | **UCT^b^** | **Cohort^c^** | **Total** |
| **LSD** | 2 | 6 | 6 | 7 | 21 | 6 | 48 |
| **DMT (ayahuasca)** | 3 | 2 | 2 | 2 | 2 | 1 | 12 |
| **Psilocybin (mescaline)** | - | 1 | 10 | - | 5 | 1 | 17 |
| **MDMA/ecstacy** | 1 | 3 | 14 | 1 | - | 1 | 20 |
| **Multiple substances** | 3 | 5 | 3 | - | 2 | 13 | 26 |
| **Other type of studies retrieved** | | | | | | | |
| **Long-lasting somatic reactions** | - | 1 | - | - | - | - | 1 |
| **HPPD** | 5 | 2 | - | - | - | **-** | 7 |
| **Total** | 14 | 20 | 35 | 10 | 30 | 22 | 131 |

**Supplementary Table S2.** List of excluded studies with reasons

| **Excluded studies** | **Title** | **Substance** | **Type of study/Reason** |
| --- | --- | --- | --- |
|  | | | **Case reports and case series** |
| **Abramson et al. 1958** | The stablemate concept of therapy as affected by LSD in schizophrenia | LSD | Case series |
| **Rolo et al. 1965** | Preliminary method for study of LSD with children | LSD | Case-report |
| **Simmons et al. 1966** | Modification of autistic behavior with LSD-25 | LSD | Case series |
| **Bowers and Freedman 1966** | “Psychedelic” experiences in acute psychoses | LSD | Case series |
| **Horowitz 1964** | The imagery of visual hallucinations. | LSD | Case series |
| **Fischer 1970** | The psycholytic treatment of a childhood schizophrenic girl | LSD | Case series |
| **Hatrick & Dewhurst 1970** | Dewhurst K: Delayed psychosis due to LSD | LSD | Case-report |
| **Ianzito et al. 1972** | Reaction to LSD in a  t ow year-old child | LSD | Case-report |
| **Heimann, 1986** | Models of experience and behavior in psychotic disorders. | Scopolamine | Case series |
| **Greer, 1985** | Using MDMA in psychotherapy. | MDMA | Case series |
| **Grinspoon & Bakalar 1986** | Can Drugs Be Used to Enhance the Psychotherapeutic  Process?* | MDMA | Case series |
| **Scher & Neppe 1991** | Carbamazepine adjunct for nonresponsive psychosis  with prior hallucinogenic abuse. | Marijuana | Case series |
| **Lu et al. 2004** | A case of prolonged peyote-induced psychosis resolved by sleep | Peyote | Case report |
| **Espiard et al. 2005** | Hallucinogen persisting perception disorder after psilocybin consumption: a case study | LSD | Case report |
| **Ikeda et al. 2005** | 5-methoxy-N,N-diisopropyltryptamine-induced flashbacks | MDMA | Case report |
| **Hermle et al. 2012** | Hallucinogen-persisting perception disorder. | LSD | Case report |
| **Lerner 2015** | Synthetic Cannabis Substances (SPS) Use and Hallucinogen Persisting Perception Disorder (HPPD): Two Case Reports | Synthetic cannabis | Case report |
| **Lerner & Lev-Ran 2015** | LSD-associated "Alice in Wonderland Syndrome"(AIWS): A Hallucinogen Persisting Perception Disorder (HPPD) Case Report | LSD | Case report |
| **Hatrick & Dewhusrt 1970** | Delayed psychosis due to LSD | LSD | Case report |
| **Szmulewicz et al. 2015** | Switch to mania after ayahuasca consumption in a man with bipolar disorder: A case report | Ayahuasca | Case report |
| **Zellner et al. 2019** | Ayahuasca-induced psychosis  in a patient with bipolar disorder. | Ayahuasca | Case report |
| **Strassman, 1992** | Human hallucinogen interactions with drugs affecting serotonergic neurotransmission | LSD | Case series |
| **Lima et al. 2002** | Sistema de monitoramento psiquiátrico em usuários do chá hoasca. | Ayahuasca | Case series |
| **Dos Santos & Strassman 2008** | Ayahuasca and psychosis | Ayahuasca | Case report |
| **Potash et al. 2009** | Persistent psychosis and medical complications after a single ingestion of MDMA “ecstasy” - A case report and review of the literature. | MDMA | Case report |
| **Umut 2011** | A mood disorder episode with an onset under chronic cannabis consumption and accompanied with psychotic features immediately after N,N-dimethyltryptamine (DMT) use: a case report. | DMT | Case report |
| **Warren 2013** | Recreational use of naturally occurring dimethyltryptamine - contributing to psychosis? | DMT | Case report |
| **Paterson et al. 2015** | N,N-Dimethyltryptamine-induced psychosis. | DMT | Case report |
| **Szmulewicz 2015** | Switch to mania after ayahuasca consumption in a man with bipolar disorder: a case report. | Ayahuasca | Case report |
| **Virani et al. 2018** | Persistent psychosis due to single dose of ecstasy. | Ecstasy | Case report |
| **Palma-Alvarez 2021** | Psychosis induced by abuse of ayahuasca: a case report | Ayahuasca | Case report |
|  |  |  | **Clinical trial and cohort studies** |
| Sandison et al. 1954 | The therapeutic value of lysergic acid diethylamide in mental illness. | LSD | No prior diagnosis for included individuals |
| **Lellan et al. 1979** | Development of psychiatric illness in drug abusers. Possible role of drug preference | Various subtances | No mention of psychosis-induced disorders |
| **Wright 1988** | Phencyclidine-induced psychosis: eight-year follow-up of ten cases. | PCP | Studies on phencyclidine are not included in our work |
| **Gouzoulis-Mayfrank 1998** | Effects of the hallucinogen psilocybin on habituation and prepulse inhibition of the startle reflex in humans | Psilocybin | No mention of psychosis-induced disorders |
| **Dolezal & Hausner** | Comparative **phenomenology** of experimental mental alterations after application of LSD, benactyzine and phenmetrzine | LSD | No mention of psychosis-induced disorders |
| **Gouzoulis-Mayfrank et al. 2005** | Psychological effects of (S)-ketamine and N,N-dimethyltryptamine **(DMT):** a double-blind, cross-over study in healthy volunteers | DMT | No mention of psychosis-induced disorders |
| **Lambert et al. 2005** | The impact of substance use disorders on clinical outcome in 643 patients with first-episode psychosis | Various subtances | No mention of psychosis-induced disorders |
| **Baggott et al. 2006** | Chronic visual changes in hallucinogen users: a web-based questionnaire. | Various subtances | Poster |
| **Daumann et al. 2010** | Neuronal correlates of visual and auditory alertness in the **DMT** and ketamine model of psychosis | DMT | No mention of psychosis-induced disorders |
| **McLean et al. 2012** | Dose-related effects of salvinorin A in humans: dissociative, hallucinogenic, and memory effects | Salvia A | No considered among the retained substances. |
| **Holze et al. 2022** | Direct comparison of the acute effects of lysergic acid diethylamide and psilocybin in a double-blind placebo-controlled study in healthy subjects | LSD | *No mention of psychotic reactions |
|  |  |  | **Reviews** |
| **Vojtĕchovský 1965** | [The contribution of hallucinogens to the theory of the etiopathogenesis of endogenic psychoses]. | Theoretical paper | No mention of psychosis-induced disorders |
| **Vojtechovsky 1984** | [CHOLINOTROPIC HALLUCINOGENS. II]. | Various substances | No mention of psychosis-induced disorders |
| **Bennet 1967** | LSD: 1967 | LSD | No mention of psychosis-induced disorders |
| **Ruzickova 1967** | [Effect of psilocybine in chronic schizophrenias. I. Clinical findings] | Psilocybin | No mention of psychosis-induced disorders |
| **Schwarz 1968** | The complications of LSD: a review of the literature. | AE | No mention of psychosis-induced disorders |
| **Lowy 1977** | Hallucinogenic Mushrooms in Guatemala | Psilocybin | No mention of psychosis-induced disorders |
| **McDonald 1980** | Mushrooms and madness. Hallucinogenic mushrooms and some psychopharmacological implications. | AE | No mention of psychosis-induced disorders |
| **Javitt 1987** | Negative schizophrenic symptomatology and the PCP (phencyclidine) model of schizophrenia. | Theoretical paper | Theoretical paper |
| **Wilkins 1989** | Clinical implications of PCP, NMDA and opiate receptors | PCP, NMDA | Studies on phencyclidine are not included in our work |
| **Pennings et al. 1998** | Clinical and toxicologic aspects of the use of Ecstasy. | Ecstasy | No mention of psychosis-induced disorders |
| **Vollenweider 1998** | Advances and Pathophysiological Models of Hallucinogenic Drug Actions in Humans: A Preamble to Schizophrenia Research | Various substances | No mention of psychosis-induced disorders |
| **Bonson et al. 2018** | Regulation of human research with LSD in the United States (1949-1987). | LSD | Paper on the historical and political aspect of human research with LSD |
| **Burgess et al. 2000** | Agony and ecstasy: a review of **MDMA** effects and toxicity | MDMA | No clear data on neuropsychiatric effects |
| **Gouzoulis-Mayfrank et al. 2002** | [Chronic neurotoxic damage in ecstasy (MDMA) users. Review of the current state of research]. | MDMA | No mention of psychosis-induced disorders |
| **Grof 1980** | LSD Psychotherapy. | LSD | No mention of psychosis-induced disorders |
| **Senoo 2003** | [Hallucinogen related mental disorder]. | Various substances | No mention of psychosis-induced disorders |
| **Barbee et al. 2009** | Analysis of mushroom exposures in Texas requiring hospitalization, 2005-2006 | Psilocybine | No mention of psychosis-induced disorders |
| **Marciniak et al. 2010** | [Poisoning with selected mushrooms with neurotropic and hallucinogenic effect]. | Psilocybine | No mention of psychosis-induced disorders |
| **De Araujo et al. 2012** | Seeing with the eyes shut: neural basis of enhanced imagery following Ayahuasca ingestion. | Ayahuasca | No mention of psychosis-induced disorders |
| **Suzuki et al. 2015** | Toxicities associated with **NBOMe** ingestion-a novel class of potent hallucinogens: a review of the literature | NBOMe | Our work does not include NBOMe. |
| **Dos Santos et al. 2016** | The current state of research on ayahuasca: A systematic review of human studies assessing psychiatric symptoms, neuropsychological functioning, and neuroimaging. | Ayahuasca | No mention of psychosis-induced disorders |
| **Wilson et al. 2018** | Clinical characteristics of primary psychotic disorders with concurrent substance abuse and substance-induced psychotic disorders: A systematic review | Various subtances | No mention of psychosis-induced disorders |
| **Zawilska & Wojciedszak 2013** | Salvia divinorum: from Mazatec medicinal and hallucinogenic plant to emerging recreational drug | Salvia divinorum | No mention of psychosis-induced disorders |
| **Murray et al. 2013** | What can we learn about schizophrenia from studying the human model, drug-induced psychosis? | Various subtances | No mention of psychosis-induced disorders |
| **Halberstadt & Geyer 2013** | Serotonergic hallucinogens as translational models relevant to schizophrenia | Various subtances | Review on the linkage between 5-HT and schizophrenia |
| **El-Khoury & Sahakian 2015** | The Association of Salvia divinorum and Psychotic Disorders: A Review of the Literature and Case Series. | Salvia A  Cannabis | No included in the retained substances. |
| **Nichols et al. 2016** | Psychedelics | Various subtances | No mention of psychosis-induced disorders |
| **Rucker et al. 2016** | Psychedelics in the treatment of unipolar mood disorders: a systematic review | Various subtances | No mention of psychosis-induced disorders |
| **Massod et al. 2018** | Treatment of Alcohol-Induced Psychotic Disorder (Alcoholic Hallucinosis)-A Systematic Review. | Various subtances | No mention of psychosis-induced disorders |
| **Lladó-Pelfort et al. 2018** | Effects of Hallucinogens on Neuronal Activity | Various subtances | Various subtances |
| **Tandon 2019** | Substance-Induced Psychotic Disorders and Schizophrenia: Pathophysiological Insights and Clinical Implications | Various subtances | No relevant information on hallucinogens regarding our inclusion criteria |
| **Van Os et al. 2019** | A systematic review and meta-analysis of the psychosis continuum: evidence for a psychosis proneness–persistence–impairment model of psychotic disorder | Various subtances | No relevant information on hallucinogens regarding our inclusion criteria |
| **Beckmann 2020** | Substance-Induced Psychosis in Youth | Various subtances | No relevant information on hallucinogens regarding our inclusion criteria |
| **Schifano et al. 2021** | New/emerging psychoactive substances and associated psychopathological consequences | Various subtances | Various subtances |
| **Anwarr et al. 2022** | New Paradigms of Old Psychedelics in Schizophrenia | Various subtances | No relevant information on psychotic-induced psychosis |
| **Leptourgos et al. 2022** | From hallucinations to synaesthesia: A circular inference account of unimodal and multimodal erroneous percepts in clinical and drug-induced psychosis. | Various subtances | Various subtances |
| **Friesen 2022** | Psychosis and psychedelics: Historical entanglements and contemporary contrast | Various subtances | Various subtances |
| **Gicas 2022** | Substance-induced psychosis and cognitive functioning: A systematic review | Various subtances | No relevant information on hallucinogens regarding our inclusion criteria |
| **Muller 2022** | Flashback phenomena after administration of LSD and psilocybin  in controlled studies with healthy participants | LSD | No relevant information on hallucinogens regarding our inclusion criteria |
| **Harmanci et al. 2023** | How are young people's mental health related to their sexual health and substance use? A systematic review of UK literature | Various subtances | No relevant information on hallucinogens regarding our inclusion criteria, except of ecstasy, however the related studies were already included in our work |
| **Zaremba et al. 2023** | Antipsychotic Drugs Efficacy in Dextromethorphan-Induced Psychosis | Dextromethorphan | This substance is not included in our systematic review |
|  |  |  | **Meta analysis** |
| **Beards et al. 2013** | Life events and psychosis: a review and meta-analysis | Various subtances | No mention of serotoninergic hallucinogens |
|  |  |  | **Guidelines** |
| **DiSclafani et al. 1981** | Drug-induced psychosis: Emergency diagnosis and management | Various subtances | Focus on other drugs that hallucinogens, besides PCP |
| **Reingardiene 2006** | [Ecstasy toxicity] | Ecstacy | No clear data on neuropsychiatrist effects |
| **Katrina et al. 2013** | Risk factors for violence in psychosis: systematic review and meta-regression analysis of 110 studies. | Various subtances | No data on psychedelics |
|  |  |  | **Articles on PCP** |
| **Schwartz 1986** | PCP intoxication in seven young children | PCP | No mention of psychosis-induced disorders |
| **McCarron 1981** | Acute phencyclidine intoxication: clinical patterns, complications, and treatment | PCP | No mention of psychosis-induced disorders |
| **McCarron 1981** | Acute phencyclidine intoxication: incidence of clinical findings in 1,000 cases | PCP | No mention of psychosis-induced disorders |
| **Wright 1988** | Phencyclidine-induced psychosis: eight-year follow-up of ten cases | PCP | No mention of psychosis-induced disorders |
| **Batisse et al. 2016** | [Cathinones use in Paris]. | PCP | No mention of psychosis-induced disorders |
| **Daziani et al. 2023** | Synthetic Cathinones and Neurotoxicity Risks: A Systematic Review | PCP | No mention of psychosis-induced disorders |

**Supplementary** **Table S3.** Assessment of non-systematics review using the SANRA scale

| **Assessment of non-systematic reviews with the Scale for the Assessment of Narrative Review Articles (SANRA)** | | | | | | | |
| --- | --- | --- | --- | --- | --- | --- | --- |
|  | **1-Justification of the article’s importance for the readership** | **2-Statement of concrete aims or formulation of questions** | **3-Description of the literature search** | **4-Referencing** | **5-Scientific reasoning** | **6-Appropriate presentation of data** | **Total score** |
| **Score of 0** | The importance is not justified | No aims or questions are formulated | The search strategy is not presented | Key statements are not supported by references | The article’s point is not based on appropriate arguments | Data are presented inadequately |  |
| **Score of 1** | The importance is alluded to, but not explicitly justified | Aims are formulated generally but not concretely or in terms of clear questions | The literature search is described briefly | The referencing of key statements is inconsistent | Appropriate evidence is introduced selectively | Data are often not presented in the most appropriate way |  |
| **Score of 2** | The importance is explicitly justified | One or more concrete aims or questions are formulated | The literature search is described in detail, including search terms and inclusion criteria | Key statements are supported by references | Appropriate evidence is generally present | Relevant outcome data are generally presented appropriately |  |
| **Article (first authors, year of publication)** |  | | | | | | |
| **Cohen 1960** ^5^ | 2 | 2 | 1 | 1 | 1 | 1 | **8** |
| **Smart & Bateman 1967** ^14^ | 2 | 1 | 0 | 1 | 1 | 1 | **6** |
| **Mogar & Aldrich 1969** ^15^ | 2 | 2 | 1 | 2 | 1 | 1 | **9** |
| **Panhuysen 1970** ^16^ | 2 | 1 | 0 | 1 | 1 | 1 | **6** |
| **Glass 1973** ^17^ | 2 | 1 | 0 | 1 | 1 | 1 | **6** |
| **McCabe 1977** ^18^ | 1 | 1 | 0 | 1 | 1 | 1 | **5** |
| **Strassman 1984** ^19^ | 2 | 2 | 1 | 2 | 2 | 2 | **11** |
| **Novak 1997** ^20^ | 1 | 1 | 1 | 1 | 1 | 1 | **6** |
| **Vollenweider et al. 1998** ^21^ | 2 | 2 | 0 | 2 | 2 | 2 | **10** |
| **McGuire 2000** ^22^ | 2 | 2 | 0 | 2 | 2 | 2 | **10** |
| **Soar et al. 2001** ^23^ | 2 | 2 | 0 | 2 | 2 | 2 | **10** |
| **Lerner et al. 2002** ^7^ | 2 | 2 | 0 | 2 | 1 | 2 | **9** |
| **Jacob & Presti 2005** ^24^ | 2 | 1 | 0 | 2 | 2 | 2 | **9** |
| **Studerus et al. 2011** ^25^ | 2 | 2 | 0 | 2 | 2 | 2 | **10** |
| **Paparelli et al. 2011** ^26^ | 2 | 2 | 0 | 2 | 2 | 2 | **10** |
| **Litjens et al. 2014** ^8^ | 2 | 2 | 0 | 2 | 2 | 2 | **10** |
| **Grammenos & Barker 2014** ^27^ | 2 | 1 | 0 | 2 | 2 | 2 | **9** |
| **Skyarbin 2018** ^6^ | 2 | 2 | 1 | 2 | 2 | 2 | **11** |
| **Wolf 2022** ^4^ | 2 | 2 | 2 | 2 | 2 | 2 | **12** |

**Scoring:** The six items that form the revised scale are rated in integers from 0 (low standard) to 2 (high standard), with 1 as an intermediate score. The maximal sum score is 12. To improve studies assessment, we examine the distribution of the scores and divided into three distinct grades the overall score as following:

Low quality narrative reviews, scores ≤ 6; Moderate quality narrative reviews, scores ≥ 7; High quality narrative reviews, scores ≥ 10

**Supplementary Table S4.** Assessment of systematic reviews using the AMSTAR-2 Criteria

| **Assessment of systematic reviews using the AMSTAR-2 Criteria** | | | | | | | | | | | | | | | | | **Overall score** |
| --- | --- | --- | --- | --- | --- | --- | --- | --- | --- | --- | --- | --- | --- | --- | --- | --- | --- |
| **AMSTAR 2 Items** | **1** | **2** | **3** | **4** | **5** | **6** | **7** | **8** | **9** | **10** | **11** | **12** | **13** | **14** | **15** | **16** |  |
| **Boutros & Bowers 1996** ^28^ | Yes | No | Yes | No | Yes | No | No | Yes | No | No | No meta | No meta | n.r | Yes | No meta | No | CL |
| **Halpern & Pope 2003 (Scoping review)** | Yes | Yes | Yes | Partial Yes | Yes | No | Yes | Yes | No | Yes | No meta | No meta | No | Yes | No meta | Yes | CL |
| **Gable 2007** ^29^ | Yes | Yes | Yes | Yes | No | No | No | Yes | No | No | No meta | No meta | No | Yes | No meta | Yes | CL |
| **Hermele et al. 2008** ^30^ | Yes | Yes | Yes | Partial Yes | No | No | No | Yes | No | No | No meta | No meta | No | Yes | No meta | Yes | CL |
| **De Gregorio et al. 2016** ^31^ | Yes | Yes | Yes | Partial Yes | Yes | No | Yes | Yes | No | Yes | No meta | No meta | No | Yes | No meta | Yes | CL |
| **Dos Santos et al. 2017** ^32^ | Yes | Yes | Yes | Yes | Yes | No | Yes | Yes | No | Yes | No meta | No meta | No | Yes | No meta | Yes | CL |
| **Martinotti et al. 2018** ^11^ | Yes | Yes | Yes | Partial yes | Yes | Yes | No | Yes | No | No | No meta | No meta | No | Yes | No meta | Yes | CL |
| **Trope et al. 2019** ^33^ | Yes | Yes | Yes | Partial yes | Yes | Yes | No | Yes | No | No | No meta | No meta | No | Yes | No meta | Yes | CL |
| **Murrie et al. 2020** ^34^ | Yes | Yes | Yes | Yes | Yes | Yes | Yes | Yes | Yes | Yes | Yes | Yes | Yes | Yes | Yes | Yes | HQ |
| **Orsolini et al. 2017** ^10^ | Yes | Yes | Yes | Partial yes | Yes | Yes | No | Yes | No | No | No meta | No meta | No | Yes | No meta | Yes | CL |
| **Orsolini et al. 2020** ^35^ | Yes | Yes | Yes | Yes | Yes | Yes | Partial yes | Yes | No | No | No meta | No meta | No | Yes | No meta | Yes | LQ |
| **Fiorentini et al. 2021** ^36^ | Yes | Yes | Yes | Yes | Yes | Yes | No | Yes | No | No | No meta | No meta | No | Yes | No meta | Yes | LQ |
| **Doyle et al. 2022** ^12^ | Yes | Yes | Yes | Yes | Yes | Yes | Yes | Partial yes | No | No | No meta | No meta | No | Yes | No meta | Yes | CL |
| **Smith et al. 2022** ^37^ | Yes | Yes | Yes | Yes | Yes | Yes | Yes | Yes | Yes | Yes | Yes | Yes | Yes | Yes | Yes | Yes | HQ |

**Source:** Shea BJ, Reeves BC, Wells G, Thuku M, Hamel C, Moran J, Moher D, Tugwell P, Welch V, Kristjansson E, Henry DA. AMSTAR 2: a critical appraisal tool for systematic reviews that include randomised or non-randomised studies of healthcare interventions, or both. BMJ. 2017 Sep 21;358:j4008.

**Abbreviations:**

CL: Critically Low-quality review

LQ: Low-quality review

MQ: Moderate quality review

HQ: High quality review

**AMSTAR 2:** This tool is composed of 16 items that can be rated as 'Yes', 'Partially Yes', or ‘No’. Seven items are recommended as critical domains (items: 2, 4, 7, 9, 11, 13, 15), affecting the overall confidence of SRs. This confidence can be classified as high (no weaknesses or one non-critical item), moderate (more than one non-critical item), low (one critical item with or without non-critical items), or critically low (more than

one critical item with or without non-critical items).

**Supplementary Table S5.** Assessment of the quality non-randomized studies using ROBINS-I tool

| **ROBINS-I tool for non-randomized studies** | | | | | | | | | |
| --- | --- | --- | --- | --- | --- | --- | --- | --- | --- |
|  | | **Pre-intervention** | | **At intervention** | **Post-intervention** | | | | **Overall risk of bias** |
| **Study** | **Type of study** | **Bias due to confounding** | **Bias in selection of participants into the study** | **Bias in classification of interventions** | **Bias due to deviations from intended interventions** | **Bias due to missing data** | **Bias in measurement of outcomes** | **Bias in selection of the reported result** | **Low/ moderate/**  **Serious/ critical** |
| **Stoll 1947** ^38^ | NRCT | Serious | Moderate | Low | Low | Critical | Critical | Critical | Critical risk |
| **Condrau 1949** ^39^ | NRCT | Serious | Moderate | Low | Low | Critical | Critical | Critical | Critical risk |
| **Busch & Johnson 1950** ^40^ | NRCT | Serious | Low | Low | Low | Critical | Critical | Critical | Critical risk |
| **De Giacomo**  **1951**^41^ | NRCT | Serious | Low | Low | Low | Critical | Critical | Critical | Critical risk |
| **Mayer-Gross**  **1951** ^42^ | NRCT | Serious | Low | Low | Low | Critical | Critical | Critical | Critical risk |
| **Forrer & Goldner 1951** ^43^ | NRCT | Serious | Low | Low | Low | Critical | Critical | Critical | Critical risk |
| **Belsanti 1952** ^44^ | NRCT | Serious | Low | Low | Low | Critical | Critical | Critical | Critical risk |
| **Hoch 1952** ^45^ | NRCT | Moderate | Low | Low | Low | Critical | Moderate | Moderate | Critical risk |
| **Katzenelbogen &Fang 1953** ^46^ | NRCT | Moderate | Low | Low | Low | Critical | Moderate | Moderate | Critical risk |
| **Liddell & Weil-Malherbe 1953** ^47^ | NRCT | Moderate | Low | Low | Low | Moderate | Moderate | Moderate | Critical risk |
| **Cholden et al. 1955** ^48^ | NRCT | Low | Low | Low | Low | Moderate | Moderate | Moderate | Moderate |
| **Bercel et al. 1956** ^49^ | NRCT | Low | Low | Low | Low | Moderate | Moderate | Moderate | Moderate |
| **Abramson 1960** ^50^ | NRCT | Low | Low | Low | Low | Moderate | Moderate | Moderate | Moderate |
| **Rümmele and Gnirss 1961**^51^ | NRCT | Moderate | Serious | Low | Low | Critical | Moderate | Moderate | Critical risk |
| **Bender et al. 1962** ^52^ | NRCT | Moderate | Serious | Low | Low | Critical | Moderate | Moderate | Critical risk |
| **Bender & Faretra 1963** ^53^ | NRCT | Moderate | Serious | Low | Low | Critical | Moderate | Moderate | Critical risk |
| **Bender 1966** ^54^ | NRCT | Moderate | Serious | Low | Low | Critical | Moderate | Moderate | Critical risk |
| **Fischer & Castile 1963** ^55^ | NRCT | Moderate | Serious | Low | Low | Critical | Moderate | Moderate | Critical risk |
| **Bergman 1971** ^56^ | Population survey | Serious | Low | Low | Low | Critical | Moderate | Moderate | Critical risk |
| **Anastasopoulos & Photiades 1962** ^57^ | NRCT | Moderate | Low | Low | Low | Critical | Moderate | Moderate | Critical risk |
| **Freedman et al. 1962** ^58^ | NRCT | Moderate | Low | Low | Low | Critical | Moderate | Moderate | Critical risk |
| **Wolbach, Miner, & Isbell 1962** ^59^ | RCT | Serious | Serious | Low | Low | Critical | Moderate | Moderate | Critical risk |
| **Bender et al. 1963** ^52^ | NRCT | Low | Low | Low | Low | Critical | Moderate | Moderate | Moderate risk |
| **Fink 1966** ^60^ | RCT | Serious | Serious | Low | Low | Critical | Moderate | Moderate | Critical risk |
| **Blumfield &Glickman 1967** ^61^ | Retrospective cohort | Serious | Serious | Low | Low | Critical | Moderate | Moderate | Critical risk |
| **Langs & Barr 1968** ^62^ | RCT | Low | unclear | Low | Low | Low | Moderate | Moderate | Moderate risk |
| **Ungerleider 1968** ^63^ | Case-control study | Serious | Serious | Low | Low | Critical | Moderate | Moderate | Moderate risk |
| **Mogar & Aldrich 1969** ^15^ | NRCT | Low | unclear | Low | Low | Low | Moderate | Moderate | Moderate risk |
| **Malleson 1971** ^64^ | Retrospective cohort | Serious | Serious | Low | Low | Critical | Moderate | Moderate | Critical risk |
| **McGlothlin & Arnold 1971** ^65^ | Longitudinal cohort | Serious | Low | Low | Low | Critical | Moderate | Moderate | Critical risk |
| **Dewhurst & Hatrick 1972** ^66^ | Retrospective cohort | Serious | Serious | Low | Low | Critical | Moderate | Moderate | Critical risk |
| **Hays & Tilley 1973** ^67^ | Case-control study | Moderate | unclear | Low | Low | Critical | Moderate | Moderate | Critical risk |
| **Breakey, Coodell, Lorenz, et al 1974** ^68^ | Case-control- retrospective cohort | Serious | unclear | Low | Low | unclear | Moderate | Moderate | Critical risk |
| **Gillin et al. 1976** ^69^ | NRCT and review | Moderate | Moderate | Low | Low | unclear | Moderate | Moderate | Moderate risk |
| **Bickel et al. 1977**^70^ | Case-control study | Serious | Moderate | Low | Low | Critical | Moderate | Moderate | Critical risk |
| **Bowers 1977** ^71^ | Prospective follow-up cohort | Serious | Moderate | Low | Low | Moderate | Moderate | Moderate | Critical risk |
| **Roy et al. 1981** ^72^ | Retrospective cohort | Serious | Serious | Low | Low | Critical | Moderate | Moderate | Critical risk |
| **Vardy & Kay 1983** ^65^ | Case-control study with follow-up | Moderate | Moderate | Low | Low | Moderate | Moderate | Moderate | Moderate risk |
| **Bowers et al. 1990** ^73^ | Prospective study | Moderate | Serious | Low | Low | Low | Moderate | Low | Critical risk |
| **Vollenweider et al. 1997** ^74^ | Two phase open study  (preliminary drug tolerance, PET phase) | Low | Moderate | Low | Low | Low | Low | Low | Moderate risk |
| **Vollenweider et al. 1999** ^75^ | RCT (simple blind) | Low | Moderate | Low | Low | Low | Low | Low | Low risk |
| **Landabaso et al. 2002** ^76^ | Prospective cohort | Moderate | Moderate | Low | Low | Moderate | Moderate | Low | Moderate risk |
| **Hasler et al. 2004** ^77^ | RCT | Low | Moderate | Low | Low | Low | Low | Low | Moderate risk |
| **Shoval et al. 2006** ^78^ | Retrospective cohort | Serious | Moderate | Low | Low | Critical | Low | Low | Critical risk |
| **Carstairs & Lee 2010** ^79^ | Retrospective cohort | Serious | Serious | Low | Low | Critical | Moderate | Moderate | Critical risk |
| **Lima & Toffoli 2012** ^80^ | Epidemiological survey | Serious | Serious | Low | Low | Critical | Moderate | Moderate | Critical risk |
| **Rugani et al. 2012** ^81^ | Case-control study | Serious | Moderate | Low | Low | Critical | Moderate | Moderate | Critical risk |
| **Niemi-Pynttari et al. 2013** ^82^ | Register based study | Serious | Low | Low | Low | Moderate | Moderate | Moderate | Serious risk |
| **Lev-Ran et al. 2014** ^83^ | Case-control study | Serious | Serious | Low | Low | Moderate | Moderate | Moderate | Serious risk |
| **Lev-Ran et al. 2015** ^84^ | Case-control study | Moderate | Moderate | Low | Low | Moderate | Moderate | Moderate | Moderate risk |
| **Krebs & Johansen 2013** ^85^ | Population study | Moderate | Moderate | Low | Low | Moderate | Moderate | Moderate | Moderate risk |
| **Johansen & Krebs 2015** ^86^ | Population study | Moderate | Moderate | Low | Low | Moderate | Moderate | Moderate | Moderate risk |
| **Garcia-Romeu et al. 2015** ^87^ | Open-label study | Moderate | Serious | Low | Low | Moderate | Moderate | Moderate | Serious risk |
| **Bouso et al. 2015** ^1^ | Case-control study | Moderate | Moderate | Low | Low | Moderate | Moderate | Moderate | Moderate risk |
| **Hendricks et al. 2014** ^88^ | Register based study | Serious | Critical | Low | Low | Moderate | Moderate | Low | Critical risk |
| **Hendricks et al. 2015** ^88^ | Register based study | Serious | Serious | Low | Low | Low | Moderate | Moderate | Serious risk |
| **Anderson et al. 2020** ^89^ | Open label study | Serious | Moderate | Low | Low | Low | Moderate | Moderate | Serious risk |
| **Jimenez-Garrido et al. 2020** ^90^ | NRCT | Moderate | Moderate | Low | Low | Low | Moderate | Moderate | Moderate risk |
| **Rognoli et al. 2023** ^91^ | Register based study | Serious | Serious | Low | Low | Low | Moderate | Moderate | Serious risk |
| **Aaronson et al. 2023** ^92^ | NRCT | Low | Low | Low | Low | Low | Low | Low | Low risk |
| **Evans et al. 2023** ^93^ | Web-based questionnaire / mixed-methods study | Serious | Serious | Low | Low | Low | Moderate | Moderate | Serious risk |
| **Simonsson et al. 2023 Or**  **Honk et al. 2024** ^94^ | Longitudinal study | Serious | Moderate | Low | Low | Low | Low | Moderate | Serious risk |

**Source:** Sterne JAC, Higgins JPT, Elbers RG, Reeves BC and the development group for ROBINS-I. Risk Of Bias In Non-randomized Studies of Interventions (ROBINS-I): detailed guidance, updated 12 October 2016. Available from http://www.riskofbias.info

The response options for an overall RoB judgement are:

(1) Low risk of bias (the study is comparable to a well-performed randomized trial);

(2) Moderate risk of bias (the study provides sound evidence for a non-randomized study but cannot be

considered comparable to a well-performed randomized trial);

(3) Serious risk of bias (the study has some important problems);

(4) Critical risk of bias (the study is too problematic to provide any useful evidence and should not be

included in any synthesis); and

(5) No information on which to base a judgement about risk of bias

**Supplementary Table S6.** Cochrane collaboration risk of bias assessment for included RCTs (RoB 2 tool).

| **Cochrane collaboration risk of bias assessment for included RCTs** | | | | | | | | |
| --- | --- | --- | --- | --- | --- | --- | --- | --- |
|  | **Random sequence generation** (selection bias) | **Allocation concealment** (selection bias) | **Blinding of participants and personnel** (performance bias) | **Blinding of outcome assessment** (detection bias) | **Incomplete outcome data** (attrition bias) | **Selective reporting** (reporting bias) | **Other bias** | **Bias score** |
| **Ayahuasca trials** | | | | | | | | |
| **Palhano-Fontes et al. 2019** ^95^ | Low risk | Low risk | Low risk | Low risk | Low risk | Low risk | - | 6- LR |
| **Dos Santos et al. 2021** ^96^ | Unclear | Low risk | Low risk | Low risk | Low risk | Unclear | - | 4 -MR |
| **Psilocybin trials** | | | | | | | | |
| **Vollenweider et al. 1999** ^3^ | Low risk | unclear | Low risk | Low risk | Low risk | Unclear |  | 4 - MR |
| **Hasler et al. 2004** ^77^ | Low risk | Low risk | Low risk | Low risk | Low risk | Unclear |  | 5- MR |
| **Grob et al. 2011** ^97^ | Unclear | Unclear | Low risk | Low risk | Low risk | Low risk | - | 4 - MR |
| **Gasser et al. 2014** ^98^ | Low risk | Low risk | Low risk | Unclear | Low risk | Low risk | - | 5 - MR |
| **Griffiths et al. 2016** ^99^ | Low risk | Low risk | Low risk | Low risk | Low risk | Unclear | - | 5 - MR |
| **Ross et al. 2016** ^100^ | Low risk | Low risk | Low risk | Low risk | Low risk | Low risk | - | 6 - LR |
| **Davis et al. 2021** ^101^ | Low risk | Low risk | Low risk | Low risk | Low risk | Low risk | - | 6 - LR |
| **Carhart-Harris et al. 2021** ^102^ | Low risk | Low risk | Low risk | Low risk | Low risk | Unclear | - | 6 - LR |
| **Goodwin et al. 2022** ^103^ | Low risk | Low risk | Low risk | Low risk | Low risk | Low risk | - | 6 - LR |
| **Bogenschutz et al. 2022** ^104^ | Low risk | Low risk | Low risk | Low risk | unclear | Low risk |  | 5 - MR |
| **Von Rotz et al. 2023** ^105^ | Low risk | Low risk | Low risk | Low risk | Low risk | Low risk | - | 6 - LR |
| **MDMA trials** | | | | | | | | |
| **Bouso et al. 2008** ^106^ | Unclear | Unclear | Low risk | Low risk | High risk | High risk | - | 2 - HR |
| **Mithoefer et al. 2011** ^107^ | Low risk | Low risk | Low risk | Low risk | Low risk | Low risk | - | 6 - LR |
| **Oehen et al. 2013** ^108^ | Low risk | Low risk | Low risk | Low risk | Low risk | Low risk | - | 6 - LR |
| **Pacey et al.** ^109^  **NCT01958593** | Low risk | High risk | High risk | Low risk | High risk | High risk | - | 2- HR |
| **Mithoefer et al. 2019** ^110^ | Low risk | Low risk | Low risk | Low risk | Low risk | Low risk | - | 6 - LR |
| **Danforth et al. 2018** ^111^ | Low risk | Low risk | Low risk | Low risk | Low risk | Low risk | - | 6 - LR |
| **Ot’alora et al. 2018** ^112^ | Low risk | Low risk | Low risk | Low risk | Low risk | Low risk | - | 6 - LR |
| **Jerome et al. 2020** ^113^ | Low risk | Low risk | Low risk | Low risk | Low risk | Low risk | - | 6 - LR |
| **Mitchell et al. 2021** ^114^ | Low risk | Low risk | Low risk | Low risk | Low risk | Low risk | - | 6 - LR |
| **Ponte et al. 2021** ^115^ | Low risk | Low risk | Low risk | Low risk | Low risk | Low risk | - | 6 - LR |
| **Brewerton et al. 2022** ^116^ | Low risk | Low risk | Low risk | Low risk | Low risk | Low risk | - | 6 - LR |
| **Nicholas et al. 2022** ^117^ | Low risk | Low risk | Low risk | Low risk | Low risk | Low risk | - | 6 - LR |
| **Wolfson et al. 2022** ^118^ | Low risk | Low risk | Low risk | Unclear | Low risk | Unclear | - | 4- MR |
| **Mitchell et al. 2023** ^119^ | Low risk | Unclear | Low risk | Low risk | Low risk | Low risk | - | 5- LR |
| **LSD trials** | | | | | | | | |
| **Isbell 1959** ^120^ | Unclear | High risk | Low risk | High risk | Low risk | Low risk | - | 3 - HR |
| **Shirvaikar & Kelkar 1966** ^121^ | Unclear | High risk | Low risk | Unclear | Unclear | Low risk | - | 1- HR |
| **Smart et al. 1966** ^122^ | Low risk | Low risk | Low risk | Unclear | Unclear | Unclear | - | 3- MR |
| **Hollister et al. 1969** ^123^ | Low risk | Low risk | High risk | Low risk | Low risk | Low risk | - | 4 - HR |
| **Tomsovic & Edwards 1970** ^124^ | Low risk | High risk | High risk | High risk | Unclear | High risk | - | 1- HR |
| **Strassman & Qualls 1994** ^125^ | Low risk | High risk | Low risk | Low risk | Low risk | Low risk | - | 5 - MR |
| **Schmid et al. 2015** ^126^ | Low risk | Low risk | Low risk | Low risk | Low risk | Low risk | - | 6 - LR |
| **Preller et al. 2018** ^127^ | Low risk | Low risk | Low risk | Low risk | Low risk | Low risk | - | 6 - LR |
| **Wießner et al. 2023** ^128^ | Low risk | Low risk | Low risk | Low risk | Low risk | Low risk | - | 6 - LR |

**Source:** Sterne JAC, Savović J, Page MJ, Elbers RG, Blencowe NS, Boutron I, Cates CJ, Cheng H-Y, Corbett MS, Eldridge SM, Hernán MA, Hopewell S, Hróbjartsson A, Junqueira DR, Jüni P, Kirkham JJ, Lasserson T, Li T, McAleenan A, Reeves BC, Shepperd S, Shrier I, Stewart LA, Tilling K, White IR, Whiting PF, Higgins JPT. RoB 2: a revised tool for assessing risk of bias in randomised trials. BMJ 2019; 366: l4898**.**

**Cochrane Risk of bias judgment:**

**Low risk of bias:** This study is judged to be at low risk of bias for all domains for this result

**Some concerns:** The trial is judged to raise some concerns in at least one domain for this result, but not to be at high risk of bias for any domain

**High risk of bias:** The trial is judged to be at high risk of bias in at least one domain for this result. Or. The trial is judged to have some concerns for multiple domains in a way that substantially lowers confidence in the result.

Studies not included Vallersnes et al. 2016^129^

**Supplementary Table S7.** Studies, reviews, and guidelines on prevention of potential adverse events and hallucinogen-induced psychosis

| **1.****Guidelines for safety and adverse events managements** | | | | | | | |
| --- | --- | --- | --- | --- | --- | --- | --- |
| **1.1. Narrative reviews** | | | | | | | |
| **Leikin et al. 1989** | Clinical features and management of intoxication due to hallucinogenic drugs | LSD  PCP  Cannabis  Cocaine | Narrative review | Authors discuss psychedelics properties and treatments of possible adverse events | * Patients exhibiting prolonged drug-induced psychosis may require a variety of treatments including ECT, lithium and l-5-hydroxytryptophan  *Authors mainly discuss stimulants induced drugs (PCP, cocaine)  *Authors propose haloperidol for LSD-psychosis | CR;  no coi | Level 3 |
| **Gouzoulis-Mayfrank 1998** | Methodological issues of human experimental research with hallucinogens | All psychedelics | Narrative review | Authors discuss the metho | *Authors discuss the study of psychosis using hallucinogens-induced psychotic states  *Authors propose to mainly include patients that are not-drug naïve  *Selection, screening, preparation, supervision, and follow-up of patients are essential | CR;  no coi | Level 3 |
| **1.2. Systematic reviews and guidelines** | | | | | | | |
| **Johnson et al. 2008** | Human Hallucinogen Research: Guidelines for Safety | RCTs settings | Guidelines | Authors propose guidelines for safety | *The most likely risk is overwhelming distress during drug action (‘bad trip’), which could lead to potentially dangerous behaviour such as leaving the study site | p.f., n.p.o. | Level 1 |
| **Johnson et al. 2018** | The abuse potential of medical psilocybin according to the 8 factors of the Controlled Substances Act in the United States | RCTs settings | Guidelines | Authors propose guidelines for safety | *Potential harms include dangerous behavior in unprepared, unsupervised users, and exacerbation of mental illness in those with or predisposed to psychotic disorders  *Safeguards against these risks include the exclusion of volunteers with personal or family history of psychotic disorders or other severe psychiatric disorders, establishing trust and rapport between session monitors and volunteer before the session, careful volunteer preparation, a safe physical session environment and interpersonal support from at least two study monitors during the session | p.f. | Level 1 |
| **Sarparast et al.**  **2022** | Drug‑drug interactions between psychiatric medications and MDMA  or psilocybin: a systematic review | RCT, epidemiological studies and case reports | Systematic review | Authors included 40 papers including 22 RCTs of MDMA administered, 4 RCTs of psilocybin and 3 epidemiological studies, gathering over 200 participants. | *Drug-drug interactions predominantly pertains to MDMA  *In available studies, most participants were young healthy adults  who were either administered a psychiatric medication only once or over several days for a maximum of 14 days, which limits extrapolation to real world clinical settings. | n.p.o. | Level 1 |
| **Breeksema et al. 2022** | Adverse events in  clinical treatments with serotonergic psychedelics and MDMA: A  mixed-methods systematic review | RCT settings | Systematic review | Authors included 24 studies reporting adverse events with serotoninergic psychedelics | *AEs are not always pre-specified, the range of potential reactions can be broad, reporting can be erratic, and terminology is often inconsistent  *Paranoia reactions and extremes anxiety was reported in some studies, but only for a very limited number of participants | no coi | Level 1 |
| **2. Selection of participants** | | | | | | | |
| **2.1. NRCT** | | | | | | | |
| **Bonson & Murphy 1996** | Alterations in responses to LSD in humans associated with chronic administration of tricyclic antidepressants, monoamine oxidase inhibitors or lithium | LSD | Cross-sectional study | Authors used a web-based questionnaire to study association of LSD and antidepressants | *Lithium and tricyclic antidepressants could increase the physiological and psychological responses to LSD, in contrast with monoamine oxidase and serotonin-selective antidepressants.  *Authors propose a parallel for induction of positive symptoms in patients with schizophrenia. | CR;  n.p.o. | Level 2 |
| **Bonson et al., 1996** | Chronic administration of serotonergic antidepressants attenuates the subjective effects of LSD in humans | LSD | Cross-sectional study | Authors used a web-based questionnaire to study association of LSD and antidepressants | *In contrast with their 1996 study, authors found no effect of lithium and tricyclic antidepressants on responses to LSD | CR:  n.p.o. | Level 2 |
| **2.2.RCT** | | | | | | | |
| **Griffiths et al., 2006** | Psilocybin can occasion mystical-type experiences having substantial and sustained personal meaning and spiritual significance | Psilocybin | RCT | Cardiovascular screening during psilocybin trial | * Psilocybin produced modestly higher elevations in blood pressure  *Based on this study, many trials propose exclusion of participants if resting blood pressure exceeded 140 systolic and 90 diastolic | LR | Level 1 |
| **Callaway & Grob, 1998** | Ayahuasca preparations and serotonin reuptake inhibitors: a potential combination for severe adverse interactions | Ayahuasca | Narrative review | Narrative review and case report | *The interaction between the potent monoamine oxidase-inhibiting harmala  alkaloids in ayahuasca and selective serotonin reuptake inhibitor  may induce a serotonin syndrome with potentially grave outcome. | LR | Level 2 |
| **3.Guidelines** | | | | | | | |
| **Johnson et al. 2008** | Human hallucinogen research: guidelines for safety | Ayahuasca | Narrative review | Authors discuss this potential interaction and present a case report | individuals are excluded who have a current or history of meeting DSM-IV criteria for schizophrenia or other psychotic disorders (unless substance-induced or due to a medical condition), or bipolar I or II disorder, which are the most important conditions to exclude for ensuring safety | LR | Level 1 |

**Supplementary Table S8.** Detail of included studies in our meta-analysis

| **Clinical studies included in our meta-analysis** | | | | | | | |
| --- | --- | --- | --- | --- | --- | --- | --- |
| **Author, year** | **Title** | **Drug** | **Type of study** | **Setting**  **Mean age, %male** | **Main findings** | **RoB 2 or ROBINS-I**  **/COIs** | **LoE**  **OCEBM Levels of Evidence^a^** |
| **Hoch, Cattell & Pennes 1952** | Effects of mescaline and LSD (d-LSD-25) | Mescaline  LSD | NRCT | 59 patients with schizophrenia received: mescaline (IV, 0.4 to 0.6 gm)(n=17), d-LSD-25 (oral LSD 10 to 120 µg) (n=21)(or both, n=21).  Patients were not benefiting from a medication.  n.a.(40.7%) | *Patients with paranoid and catatonic schizophrenia presented an increase of their symptoms during drug intake. Most, but not all patients with pseudoneurotic schizophrenia were improved  *Intravenous mescaline created acute and reversible marked deterioration compared to oral LSD. Hallucinations were essentially visual, and sexual material occurred  *With LSD patients presented frequent emotional disturbances and with both drug an increase of disorganization occurred  *Authors comment that the low incidence of auditory hallucinations is a striking contrast to the high incidence of such hallucinations in drug-free schizophrenic patients. Moreover, the predominance of visual hallucinations in schizophrenics corresponds to the same.  *For LSD, 4.8 (2/41) patients presented long-lasting psychotic reactions, and 19% (4/21) patients presented marked deterioration during the trip | CR;  n.m. | Level 2 |
| **Cohen 1960** | Lysergic Acid Diethylmaide: side effects and complications | LSD | UCT | A total of 62 physicians who had experience with prescription of LSD or mescaline were asked on psychiatric side effects of treatments. (n.a) | *Dosages of LSD ranged from 25-1500 mcg.  *A prevalence of 0.18% (1.8/1000) of prolonged psychotic reaction (> 48h) was reported | MR;  n.m. | Level 2 |
| **Baker et al. 1967**  **(reported in Smart et al. 1967)** | LSD Psychotherapy; LSD Psycho-Exploration: Three Reports | LSD | UCT | A total of 150 patients received up to 10 sessions of LSD. (n.a) | *Reported in Smart et al. 1967: Unfavourable Reactions to LSD:  A Review and Analysis of the Available Case Reports  *Authors found a 2.6% (3/158 patients) incidence of long-lasting psychotic reactions. | CR;  pf | Level 3 |
| **Fink et al. 1966** | Prolonged adverse  reactions to LSD in psychotic subjects. | LSD | NRCT | One or multiple IV LSD session (0.5µg/kg to 10 µg/Kg) -or placebo- to 65 patients with schizophrenia.  36 year-old(n.a.) | *Patients received LSD, or a placebo medication  *Authors found a 3.2% (3/92) incidence of long-lasting psychotic reactions, described as basically exacerbations of preexisting psychopathology with accompanying signs of a confusional delirium | CR;  pf, npo | Level 2 |
| **Leuner 1965**  **(reported in Smart et al. 1967)** | Present state of psycholytic therapy and its possibilities | LSD | UCT | A total of 82 patients received an average of 27 sessions of LSD. (n.a) | *Reported in Smart et al. 1967: Unfavourable Reactions to LSD:  A Review and Analysis of the Available Case Reports  *Authors found a rate 3.6% (3/82 patients) incidence of long-lasting psychotic reactions. | CR;  pf | Level 3 |
| **Bergman 1971** | Navajo peyote use: Its apparent safety | Mescaline (Peyote) | Population survey. Description of 3 cases reports | Authors report a 4-year community psychiatric service mental among Navajo Indians that are peyote users. 30000 participants. | *Authors estimated the number of psychotic outbreaks among Navajo members of the Native American Church of 1/70,000 peyote ingestions  *Combination of alcohol and peyote can trigger short-duration paranoid trips.  *Authors describe cases of patients with schizophrenia diagnosis that attended many ceremonial sessions without important adverse events | CR;  n.m. | Level 2 |
| **Malleson 1971** | Acute Adverse Reactions to LSD in Clinical and  Experimental Use in the United Kingdom | LSD | Retrospective cohort | Report by clinicians of their practice with LSD. (n.a) | *On 4300 individuals who received one or multiple session of LSD, 37 presented psychedelic-induced psychosis  *Of these 37 patients, 10 patients presented a persistent and chronic psychotic state, and 19 individuals recovered completely (7 over 3 months, 3 between 2 weeks to 3 months and 9 within 2 weeks) | CR;  n.m. | Level 2 |
| **McGlothlin & Arnold 1971** | LSD revisited. A ten-year follow-up of medical LSD use | LSD | 10-year follow-up study | 247 persons who received LSD in nonmedical or psychotherapeutic  setting. 44(66%) | * 58% of subjects reported some lasting effect 6 months following 3 sessions of 200μg LSD, as lower anxiety to stress. There is little evidence on measurable, lasting personality or behavior changes  * 36 of the 247 participants, and only 4 of the 32 participants with serious psychiatric disorders presented ‘LSD-like recurrences’, that can be classified as minor psychotic or psychosomatic symptoms  *One case of prolonged psychosis with a hospitalization of 1 week of the 247 LSD users, occurring after a third LSD experience (1/276 sessions) | CR;  p.f. | Level 2 |
| **Novak 1997** | LSD before Leary Sidney Cohen’s Critique of 1950s Psychelelic Drug Research | LSD | Narrative review | Narrative review on historical concepts | *Reports a long-lasting psychosis (>48H) at a rate of 1 case out of 247 individuals who received LSD | CR n.m. | Level 3 |
| **Perez et al. 2023** | Psilocybin-assisted therapy for depression: A systematic review and dose-response meta-analysis of human studies | Psilocybin | Meta-analysis | A total of 366 patients included in several RCt were included in this meta-analysis. Doses of psilocyin varied from 3 mg to 40 mg. 366(46.8%) | *The determined 95% effective doses per day (ED95) were 8.92, 24.68,  and 36.08 mg/70 kg for patients with secondary depression, primary depression, and both subgroups, respectively.  *Authors found we found significant dose-response associations for various side effects, including physical discomfort, blood pressure increase, nausea/vomiting,  headache/migraine, and the risk of prolonged psychosis | LR;  p.f. | Level 1 |
| **Studerus et al. 2011** | Acute, subacute and long-term subjective effects of psilocybin in healthy humans: a pooled analysis of experimental studies | Psilocybin | Review of different clinical trials | Narrative review of the prospective follow-up of patients included in different clinical trials. 227 subjects experimented psilocybin with follow-up of possible adverse events | *Authors identified a pool of 8 RCTs published between 1999 and 2008  *The analysis included 110 healthy subjects who had received 1-4 oral doses of psilocybin (45-315 µg/kg body weight)  *Acute adverse drug reactions, characterized by strong dysphoria and/or anxiety/panic, occurred only in the two highest dose conditions in a relatively small proportion of subjects (2/110)  *Authors found no indication for subsequent drug abuse, persisting perception disorders, prolonged psychosis or other long-term impairments of functioning in any of our subjects. | LR;  npo, pf | Level 1 |
| **Lima & Toffoli 2011** | An epidemiological surveillance system by the UDV: mental health  recommendations concerning the religious use of hoasca | Ayahuasca | Epidemiological survey | Authors report observations obtained through a surveillance program from 1994 up to 2007. 130000 participants | *Authors report 20 cases of psychotic-like phenomena, with only 4 cases of an immediate temporal relationship without a preexistent psychiatric history, and 5 cases of beginning or worsening with preexisting psychiatric history and/or active disorder.  *Authors estimate the number of psychotic occurrences as 1/50000 ingestions | CR;  n.m. | Level 2 |
| **Niemi-Pynttari et al. 2013** | Substance-induced psychoses converting into schizophrenia: a register-based study of 18,478 Finnish inpatient cases | LSD | Register-based study | Retrospective analysis of the nationwide Finnish hospital discharge register with follow-up of patients (1987-2003). Total of 18.478 patients, with 84 HIP. 24.3(7) | *Eight-year cumulative risk to receive a schizophrenia spectrum disorder diagnosis reported:  46%(95%CI, 35-57%) for cannabis-induced psychosis  30%(95%CI, 14-46%) for amphetamine-induced psychosis  24%(95%CI, n.a.) for hallucinogens-induced psychosis (n=15/84 ; N=3)  *HR (95%CI): 2.69 (1.59-4.55) for hallucinogens. PCP accounted for 1/3 of studies  *Total of 18.478 patients, with 84 patients presenting a hallucinogen-induces psychosis. Of those patients 15(17.8%) converged to a diagnosis of schizophrenia | SR;  p.f. | Level 2 |
| **Starzer et al. 2018** | Rates and predictors  of conversion to schizophrenia or bipolar disorder following substance-induced psychosis. | Various hallucinogens | Register based study | Information extracted from the Danish Civil Registration System. 6788 patients with a diagnosis of SIP.  n.a(78%) | *The highest conversion rate was found for cannabis-induced psychosis, with HR= 47.4% (95% CI 54.7–52.3) converting to either schizophrenia or bipolar disorder  *Alcohol induced 34% of the psychoses, cannabis 22%, and mixed/other substances 27% | CR;  p.i. | Level 2 |
| **Rognoli et al. 2023** | Transition From Substance-Induced Psychosis to  Schizophrenia Spectrum Disorder or Bipolar Disorder | Various hallucinogens | Register based study | Information extracted from the Norwegian Patient Registry from 2010 to 2015. 3187 patients with a diagnosis of SIP.33.6±12.3(73.4%) | *The highest conversion rate was found for Cannabis-induced psychosis CH= 36%(95% CI 31.4-41) converting to schizophrenia  *The risk of transition of psychedelics-induced psychosis was obtained directly | SR;  p.f. | Level 2 |

**Supplementary Fig S1.** Systematic review PRISMA flowchart

***Additional records identified though other sources,** n = 27

***Unique titles identified though database search,** n = 2138

• Electronic databases, n= 2138

(PUBMED, n= 597; EMBASE, n= 1770; PsyARTICLES, n= 925; PsyINFO, n= 900).

• Registry at clinicaltrials.gov, n= 67

• International Clinical Trials Registry Platform, n= 10

## Identification

**Excluded,** n= 121

Data not relevant to our research subject, or of insufficient quality, n= 121

**Unique titles identified,**

n= 2186

## Screening

**Excluded,** n= 1832

Article not relevant to research subject, n= 1750

Animal studies, n=52

Articles on genetics, n= 24

Duplication, n= 6

**Screening of trial protocols, and/or abstracts,**

n= 2055

**Clinical trials, randomized clinical trials, reviews and meta-analysis full text reviewed for eligibility,**

n= 223

## Eligibility

**Exclusion after review of full-text articles,** n= 92

Case reports and case series,

n= 29

Clinical trials and cohorts’ studies not mentioning psychotic reactions, n= 9

Review not eligible to our inclusion criteria, n= 44

Articles on PCP, n= 6

**Studies included in qualitative synthesis,**

n= 131

## Included

**Studies included in quantitative synthesis,**

n= 131

**Supplementary Fig S2.** Funnel plot and Trim and fill method


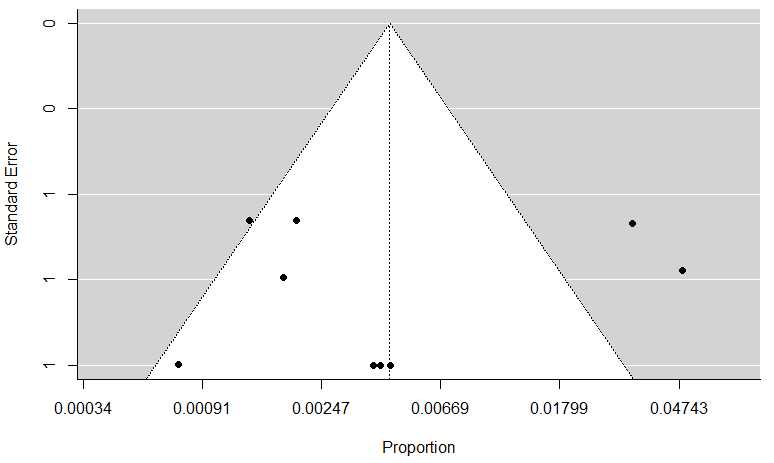


Trim and fill method for publications bias


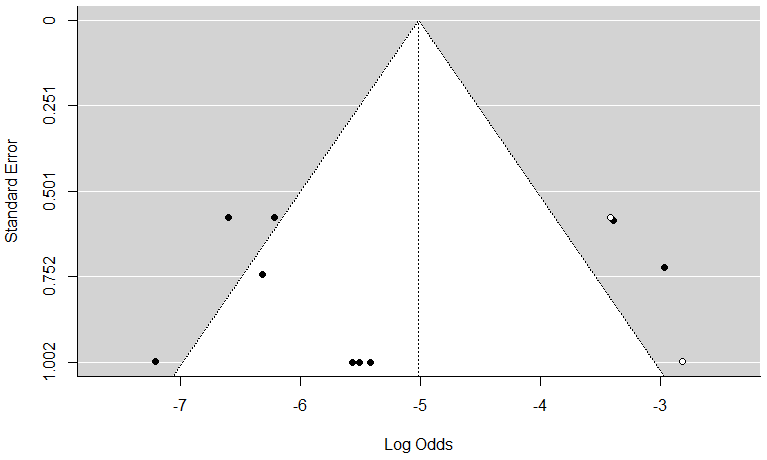


Prediction

pred ci.lb ci.ub pi.lb pi.ub

0.0066 0.0025 0.0174 0.0003 0.1181

Egger regression test

Regression Test for Funnel Plot Asymmetry

Model: mixed-effects meta-regression model

Predictor: standard error

**Test for Funnel Plot Asymmetry:** z = -0.5333, p = 0.5939

Limit Estimate (as sei -> 0): b = -4.1970 (CI: -8.8606, 0.4667)


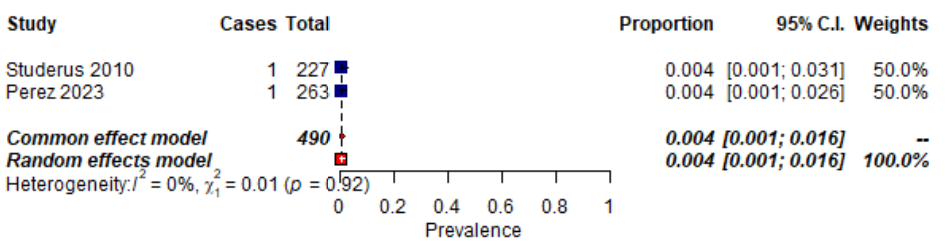


**Supplementary Fig S3.** Sensitivity analysis of psychedelic-induced psychosis according to the number of sessions across healthy individuals, patients with depression, and patients with schizophrenia. Included studies were restricted to studies presenting low risk of bias.


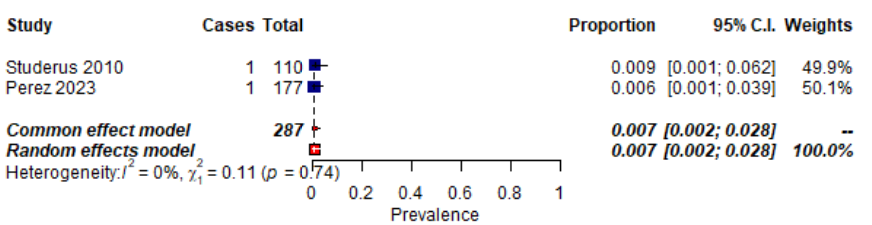


**Supplementary Fig S4.** Sensitivity analysis for the incidence of psychedelic-induced psychosis in healthy individuals and patients with depression. Included studies were restricted to studies presenting low risk of bias.

**Supplementary Information 5.** References of all included studies

1. Bouso, J. C. *et al.* Long-term use of psychedelic drugs is associated with differences in brain structure and personality in humans. *Eur. Neuropsychopharmacol. J. Eur. Coll. Neuropsychopharmacol.* **25**, 483–492 (2015).

2. Vollenweider, F. X. *et al.* Positron emission tomography and fluorodeoxyglucose studies of metabolic hyperfrontality and psychopathology in the psilocybin model of psychosis. *Neuropsychopharmacol. Off. Publ. Am. Coll. Neuropsychopharmacol.* **16**, 357–372 (1997).

3. Vollenweider, F. X., Vontobel, P., Hell, D. & Leenders, K. L. 5-HT modulation of dopamine release in basal ganglia in psilocybin-induced psychosis in man--a PET study with [11C]raclopride. *Neuropsychopharmacol. Off. Publ. Am. Coll. Neuropsychopharmacol.* **20**, 424–433 (1999).

4. Wolf, G. *et al.* Could psychedelic drugs have a role in the treatment of schizophrenia? Rationale and strategy for safe implementation. *Mol. Psychiatry* **28**, 44–58 (2023).

5. Cohen, S. Lysergic acid diethylamide: side effects and complications. *J. Nerv. Ment. Dis.* **130**, 30–40 (1960).

6. Skryabin, V. Y., Vinnikova, M., Nenastieva, A. & Alekseyuk, V. Hallucinogen persisting perception disorder: A literature review and three case reports. *J. Addict. Dis.* **37**, 268–278 (2018).

7. Lerner, A. G. *et al.* Flashback and Hallucinogen Persisting Perception Disorder: clinical aspects and pharmacological treatment approach. *Isr. J. Psychiatry Relat. Sci.* **39**, 92–99 (2002).

8. Litjens, R. P. W., Brunt, T. M., Alderliefste, G.-J. & Westerink, R. H. S. Hallucinogen persisting perception disorder and the serotonergic system: a comprehensive review including new MDMA-related clinical cases. *Eur. Neuropsychopharmacol. J. Eur. Coll. Neuropsychopharmacol.* **24**, 1309–1323 (2014).

9. Halpern, J. H. & Pope, H. G. J. Hallucinogen persisting perception disorder: what do we know after 50 years? *Drug Alcohol Depend.* **69**, 109–119 (2003).

10. Orsolini, L. *et al.* The ‘Endless Trip’ among the NPS Users: Psychopathology and Psychopharmacology in the Hallucinogen-Persisting Perception Disorder. A Systematic Review. *Front. psychiatry* **8**, 240 (2017).

11. Martinotti, G. *et al.* Hallucinogen Persisting Perception Disorder: Etiology, Clinical Features, and Therapeutic Perspectives. *Brain Sci.* **8**, (2018).

12. Doyle, M. A. *et al.* Hallucinogen persisting perceptual disorder: a scoping review covering frequency, risk factors, prevention, and treatment. *Expert Opin. Drug Saf.* **21**, 733–743 (2022).

13. Simonsson, O. *et al.* Longitudinal associations between psychedelic use and unusual visual experiences in the United States and the United Kingdom. *J. Psychopharmacol.* 02698811231218931 (2023) doi:10.1177/02698811231218931.

14. Smart, R. G. & Bateman, K. Unfavourable reactions to LSD: a review and analysis of the available case reports. *Can. Med. Assoc. J.* **97**, 1214–1221 (1967).

15. Mogar, R. E. & Aldrich, R. W. The use of psychedelic agents with autistic schizophrenic children. *Behav. Neuropsychiatry* **1**, 44–50 (1969).

16. Panhuysen, L. H. Undesirable side effects of LSD administration. *Ned. Tijdschr. Geneeskd.* 723–727 (1970).

17. Glass, G. S. PSYCHEDELIC DRUGS, STRESS, AND THE EGO. *J. Nerv. Ment. Dis.* **156**, 232–241 (1973).

18. McCabe, O. L. Psychedelic Drug Crises: Toxicity and Therapeutics. *J. Psychedelic Drugs* **9**, 107–121 (1977).

19. Strassman, R. J. Adverse reactions to psychedelic drugs. A review of the literature. *J. Nerv. Ment. Dis.* **172**, 577–595 (1984).

20. Novak, S. J. LSD before Leary. Sidney Cohen’s critique of 1950s psychedelic drug research. *Isis; an Int. Rev. devoted to Hist. Sci. its Cult. Influ.* **88**, 87–110 (1997).

21. Vollenweider, F. X., Vollenweider-Scherpenhuyzen, M. F. I., Bäbler, A., Vogel, H. & Hell, D. Psilocybin induces schizophrenia-like psychosis in humans via a serotonin-2 agonist action. *Neuroreport* **9**, (1998).

22. McGuire, P. Long term psychiatric and cognitive effects of MDMA use. *Toxicol. Lett.* **112**–**113**, 153–156 (2000).

23. Soar, K., Turner, J. J. D. & Parrott, A. C. Psychiatric disorders in Ecstasy (MDMA) users: a literature review focusing on personal predisposition and drug history. *Hum. Psychopharmacol.* **16**, 641–645 (2001).

24. Jacob, M. S. & Presti, D. E. Endogenous psychoactive tryptamines reconsidered: an anxiolytic role for dimethyltryptamine. *Med. Hypotheses* **64**, 930–937 (2005).

25. Studerus, E., Kometer, M., Hasler, F. & Vollenweider, F. X. Acute, subacute and long-term subjective effects of psilocybin in healthy humans: a pooled analysis of experimental studies. *J. Psychopharmacol.* **25**, 1434–1452 (2011).

26. Paparelli, A., Di Forti, M., Morrison, P. D. & Murray, R. M. Drug-induced psychosis: how to avoid star gazing in schizophrenia research by looking at more obvious sources of light. *Front. Behav. Neurosci.* **5**, 1 (2011).

27. Grammenos, D. & Barker, S. A. On the transmethylation hypothesis: stress, N,N-dimethyltryptamine, and positive symptoms of psychosis. *J. Neural Transm.* **122**, 733–739 (2015).

28. Boutros, N. N. & Bowers, M. B. J. Chronic substance-induced psychotic disorders: state of the literature. *J. Neuropsychiatry Clin. Neurosci.* **8**, 262–269 (1996).

29. Gable, R. S. Risk assessment of ritual use of oral dimethyltryptamine (DMT) and harmala alkaloids. *Addiction* **102**, 24–34 (2007).

30. Hermele, M., Ran, Y., Lee, P. A. & Wen, X.-G. Properties of an algebraic spin liquid on the kagome lattice. *Phys. Rev. B* **77**, 224413 (2008).

31. De Gregorio, D., Comai, S., Posa, L. & Gobbi, G. d-Lysergic Acid Diethylamide (LSD) as a Model of Psychosis: Mechanism of Action and Pharmacology. *Int. J. Mol. Sci.* **17**, (2016).

32. Dos Santos, R., Bouso, J. C. & Hallak, J. Ayahuasca, dimethyltryptamine, and psychosis: A systematic review of human studies. *Ther. Adv. Psychopharmacol.* **7**, 1–17 (2017).

33. Trope, A. *et al.* Psychedelic-Assisted Group Therapy: A Systematic Review. *J. Psychoactive Drugs* **51**, 174–188 (2019).

34. Murrie, B., Lappin, J., Large, M. & Sara, G. Transition of Substance-Induced, Brief, and Atypical Psychoses to Schizophrenia: A Systematic Review and Meta-analysis. *Schizophr. Bull.* **46**, 505–516 (2020).

35. Orsolini, L. *et al.* How does ayahuasca work from a psychiatric perspective? Pros and cons of the entheogenic therapy. *Hum. Psychopharmacol.* **35**, e2728 (2020).

36. Fiorentini, A. *et al.* Substance-Induced Psychoses: An Updated Literature Review. *Front. psychiatry* **12**, 694863 (2021).

37. Smith, K. W., Sicignano, D. J., Hernandez, A. V & White, C. M. MDMA-Assisted Psychotherapy for Treatment of Posttraumatic Stress Disorder: A Systematic Review With Meta-Analysis. *J. Clin. Pharmacol.* **62**, 463–471 (2022).

38. Stoll, W. A. Lysegsaure-diathylamid, ein Phantastikum aus der Mutterkorngruppe," Schweizer Archiv fur Neurologie und Psychiatrie. *Schweiz. Arch. Neurol. Psychiatr.* **60**, 279–323 (1947).

39. CONDRAU, G. Klinische Erfahrungen an Geisteskranken mit Lysergsäure-Diäthylamid. *Acta Psychiatr. Scand.* **24**, 9–32 (2007).

40. Busch, A. & Johnson, W. L.S.D. 25 as an aid in psychotherapy; preliminary report of a new drug. *Dis. Nerv. Syst.* **11**, 241–243 (1950).

41. De Giacomo, U. La catatonie toxique expérimentale. *Acta Neurol. (Napoli).* **7**, 5–10 (1951).

42. Mayer-Gross, W. Experimental Psychoses and Other Mental Abnormalities Produced by Drugs. *Br. Med. J.* **2**, 317 LP – 321 (1951).

43. Forrer, G. R. & Goldner, R. D. EXPERIMENTAL PHYSIOLOGICAL STUDIES WITH LYSERGIC ACID DIETHYLAMIDE (LSD-25). *A.M.A. Arch. Neurol. Psychiatry* **65**, 581–588 (1951).

44. Belsanti, R. Modificazioni neuro-psico-biochimiche indotte dalla dietilamide dell’acido lisergico in schizofrenici e frenastenici. *Acta neurol.(Napoli)* **7**, 25 (1952).

45. Hoch, P. H., Cattell, J. P. & Pennes, H. H. EFFECTS OF MESCALINE AND LYSERGIC ACID (d-LSD-25). *Am. J. Psychiatry* **108**, 579–584 (1952).

46. Katzenelbogen, S. & Fang, A. D. Narcosynthesis effects of sodium amytal, methedrine and L.S.D-25. *Dis. Nerv. Syst.* **14**, 85–8 (1953).

47. Liddell, D. W. & Weil-Malherbe, H. THE EFFECTS OF METHEDRINE AND OF LYSERGIC ACID DIETHYLAMIDE ON MENTAL PROCESSES AND ON THE BLOOD ANDRENALINE LEVEL. *J. Neurol. Neurosurg. Psychiatry* **16**, 7–13 (1953).

48. CHOLDEN, L. S., KURLAND, A. & SAVAGE, C. CLINICAL REACTIONS AND TOLERANCE TO LSD IN CHRONIC SCHIZOPHRENIA. *J. Nerv. Ment. Dis.* **122**, (1955).

49. Bercel, N. A., Travis, L. E. E. E., Olinger, L. B., Dreikurs, E. & Polos, M. G. Model Psychoses Induced by LSD-25 in Normals: I. Psychophysiological Investigations, with Special Reference to the Mechanism of the Paranoid Reaction. *A.M.A. Arch. Neurol. Psychiatry* **75**, 588–611 (1956).

50. Abramson, H. A. *The Use of LSD in Psychotherapy: Transactions of a Conference on D-Lysergic Acid Diethylamide (LSD-25), April 22, 23 and 24, 1959, Princeton, N. J.* (Josiah Macy, Jr. Foundation, 1960).

51. RUEMMELE, W. & GNIRSS, F. [Research on psiloybin, a psychotropic substance from Psilocybe mexicana]. *Schweiz. Arch. Neurol. Neurochir. Psychiatr.* **87**, 365–85 (1961).

52. Bender, L., Goldschmidt, L., Sankar, D. V. S. & Freedman, A. M. Treatment of Autistic Schizophrenic Children with LSD-25 and UML-491 BT - Recent Advances in Biological Psychiatry: Volume IV: The Proceedings of the Sixteenth Annual Convention and Scientific Program of the Society of Biological Psychiatry, Atlantic Cit. in (ed. Wortis, J.) 170–179 (Springer US, 1962). doi:10.1007/978-1-4684-8306-2_17.

53. Bender, L., Faretra, G. & Cobrinik, L. LSD and UML treatment of hospitalized disturbed children. *Recent Adv. Biol. Psychiatry* **5**, 84–92 (1963).

54. Bender, L. D-lysergic acid in the treatment of the biological features of childhood schizophrenia. *Dis. Nerv. Syst.* **7 Suppl**, 43–46 (1966).

55. Fischer, G. & Castile, D. *An Investigation to Determine the Therapeutic Effectiveness of LSD-25 and Psilocybin on Hospitalized Severely Emotionally Disturbed Children*. (Purdue University Archives and Special Collections, 1963).

56. Bergman, R. L. Navajo peyote use: Its apparent safety. *The American Journal of Psychiatry* vol. 128 695–699 (1971).

57. Anastasopoulos, G. & Photiades, H. Effects of LSD-25 on Relatives of Schizophrenic Patients. *J. Ment. Sci.* **108**, 95–98 (1962).

58. FREEDMAN, A. M., EBIN, E. V. A. V & WILSON, E. A. Autistic Schizophrenic Children: An Experiment in the Use of D-Lysergic Acid Diethylamide (LSD-25). *Arch. Gen. Psychiatry* **6**, 203–213 (1962).

59. Wolbach, A. B., Miner, E. J. & Isbell, H. Comparison of psilocin with psilocybin, mescaline and LSD-25. *Psychopharmacologia* **3**, 219–223 (1962).

60. Fink, M., Simeon, J., Haque, W. & Itil, T. Prolonged adverse reactions to LSD in psychotic subjects. *Arch. Gen. Psychiatry* **15**, 450–454 (1966).

61. Blumenfield, M. & Glickman, L. Ten months experience with LSD users admitted to county psychiatric receiving hospital. *New York State Journal of Medicine* vol. 67 1849–1853 (1967).

62. Langs, R. J. & Barr, H. L. LYSERGIC ACID DIETHYLAMIDE (LSD-25) AND SCHIZOPHRENIC REACTIONS. *J. Nerv. Ment. Dis.* **147**, (1968).

63. Ungerleider, J. T., Fisher, D. D., Fuller, M. & Caldwell, A. The ‘bad trip’--the etiology of the adverse LSD reaction. *Am. J. Psychiatry* **124**, 1483–1490 (1968).

64. Malleson, N. Acute Adverse Reactions to Lsd in Clinical and Experimental use in the United Kingdom. *Br. J. Psychiatry* **118**, 229–230 (1971).

65. McGlothlin, W. H. & Arnold, D. O. LSD Revisited: A Ten-Year Follow-up of Medical LSD Use. *Arch. Gen. Psychiatry* **24**, 35–49 (1971).

66. Dewhurst, K. & Hatrick, J. A. Differential diagnosis and treatment of lysergic acid diethylamide induced psychosis. *Practitioner* **209**, 327–332 (1972).

67. Hays, P. & Tilley, J. R. The Differences between LSD Psychosis and Schizophrenia. *Can. Psychiatr. Assoc. J.* **18**, 331–333 (1973).

68. Breakey, W. R., Goodell, H., Lorenz, P. C. & McHugh, P. R. Hallucinogenic drugs as precipitants of schizophrenia. *Psychol. Med.* **4**, 255–261 (1974).

69. Gillin, J. C., Kaplan, J., Stillman, R. & Wyatt, R. J. The psychedelic model of schizophrenia: The case of N,N -dimethyltryptamine. *The American Journal of Psychiatry* vol. 133 203–208 (1976).

70. Bickel, P., Dittrich, A. & Schopf, J. Effekte von N,N-Dimethyltryptamin (DMT) auf Psychotizismus-Tests. *Pharmakopsychiat* **10**, 10–14 (1977).

71. Bowers, M. B. J. Psychoses precipitated by psychotomimetic drugs. A follow-up study. *Arch. Gen. Psychiatry* **34**, 832–835 (1977).

72. Roy, A. LSD and onset of schizophrenia. *Can. J. Psychiatry.* **26**, 64–65 (1981).

73. Bowers Jr., M. B., Mazure, C. M., Nelson, J. C. & Jatlow, P. I. Psychotogenic Drug Use and Neuroleptic Response. *Schizophr. Bull.* **16**, 81–85 (1990).

74. Vollenweider, F. X. *et al.* Metabolic hyperfrontality and psychopathology in the ketamine model of psychosis using positron emission tomography (PET) and [18F]fluorodeoxyglucose (FDG). *Eur. Neuropsychopharmacol. J. Eur. Coll. Neuropsychopharmacol.* **7**, 9–24 (1997).

75. Vollenweider, F., Vollenweider-Scherpenhuyzen, M., Bäbler, A., Vogel, H. & Hell, D. Psilocybin induces schizophrenia-like psychosis in humans via a serotonin-2 agonist action. *Neuroreport* **9**, 3897–3902 (1999).

76. Landabaso, M. A. *et al.* Ecstasy-induced psychotic disorder: six-month follow-up study. *Eur. Addict. Res.* **8**, 133–140 (2002).

77. Hasler, F., Grimberg, U., Benz, M. A., Huber, T. & Vollenweider, F. X. Acute psychological and physiological effects of psilocybin in healthy humans: a double-blind, placebo-controlled dose-effect study. *Psychopharmacology (Berl).* **172**, 145–156 (2004).

78. Shoval, G. *et al.* Substance Use, Suicidality, and Adolescent-Onset Schizophrenia: An Israeli 10-Year Retrospective Study. *J. Child Adolesc. Psychopharmacol.* **16**, 767–775 (2006).

79. Carstairs, S. D. & Cantrell, F. L. Peyote and mescaline exposures: a 12-year review of a statewide poison center database. *Clin. Toxicol. (Phila).* **48**, 350–353 (2010).

80. Lima, F. A. S., Tófoli, L. F., Labate, B. & Jungaberle, H. An epidemiological surveillance system by the UDV: mental health recommendations concerning the religous use of hoasca. in (2011).

81. Rugani, F. *et al.* Symptomatological features of patients with and without Ecstasy use during their first psychotic episode. *Int. J. Environ. Res. Public Health* **9**, 2283–2292 (2012).

82. Niemi-Pynttäri, J. A. *et al.* Substance-Induced Psychoses Converting Into Schizophrenia. *J. Clin. Psychiatry* **74**, e94–e99 (2013).

83. Lev-Ran, S., Feingold, D., Frenkel, A. & Lerner, A. G. Clinical characteristics of individuals with schizophrenia and hallucinogen persisting perception disorder: a preliminary investigation. *J. Dual Diagn.* **10**, 79–83 (2014).

84. Lev-Ran, S., Feingold, D., Rudinski, D., Katz, S. & Arturo, L. G. Schizophrenia and hallucinogen persisting perception disorder: A clinical investigation. *Am. J. Addict.* **24**, 197–199 (2015).

85. Krebs, T. S. & Johansen, P.-Ø. Psychedelics and mental health: a population study. *PLoS One* **8**, e63972 (2013).

86. Johansen, P.-Ø. & Krebs, T. Psychedelics not linked to mental health problems or suicidal behavior: A population study. *J. Psychopharmacol.* **29**, (2015).

87. Garcia-Romeu, A., Griffiths, R. R. & Johnson, M. W. Psilocybin-occasioned mystical experiences in the treatment of tobacco addiction. *Curr. Drug Abuse Rev.* **7**, 157–164 (2014).

88. Hendricks, P. S., Thorne, C. B., Clark, C. B., Coombs, D. W. & Johnson, M. W. Classic psychedelic use is associated with reduced psychological distress and suicidality in the United States adult population. *J. Psychopharmacol.* **29**, 280–288 (2015).

89. Anderson, B. T. *et al.* Psilocybin-assisted group therapy for demoralized older long-term AIDS survivor men: An open-label safety and feasibility pilot study. *EClinicalMedicine* **27**, 100538 (2020).

90. Jiménez-Garrido, D. F. *et al.* Effects of ayahuasca on mental health and quality of life in naïve users: A longitudinal and cross-sectional study combination. *Sci. Rep.* **10**, 4075 (2020).

91. Rognli, E. B., Heiberg, I. H., Jacobsen, B. K., Høye, A. & Bramness, J. G. Transition From Substance-Induced Psychosis to Schizophrenia Spectrum Disorder or Bipolar Disorder. *Am. J. Psychiatry* **180**, 437–444 (2023).

92. Aaronson, S. T. *et al.* Single-Dose Synthetic Psilocybin With Psychotherapy for Treatment-Resistant Bipolar Type II Major Depressive Episodes: A Nonrandomized Controlled Trial. *JAMA psychiatry* (2023) doi:10.1001/jamapsychiatry.2023.4685.

93. Evans, J. *et al.* Extended difficulties following the use of psychedelic drugs: A mixed methods study. *PLoS One* **18**, e0293349 (2023).

94. Honk, L. *et al.* Longitudinal associations between psychedelic use and psychotic symptoms in the United States and United Kingdom. *J. Affect. Disord.* (2024) doi:https://doi.org/10.1016/j.jad.2024.01.197.

95. Palhano-Fontes, F. *et al.* Rapid antidepressant effects of the psychedelic ayahuasca in treatment-resistant depression: a randomized placebo-controlled trial. *Psychol. Med.* **49**, 655–663 (2019).

96. Dos Santos, R. G. *et al.* Ayahuasca Improves Self-perception of Speech Performance in Subjects With Social Anxiety Disorder: A Pilot, Proof-of-Concept, Randomized, Placebo-Controlled Trial. *J. Clin. Psychopharmacol.* **41**, 540–550 (2021).

97. Grob, C. S. *et al.* Pilot study of psilocybin treatment for anxiety in patients with advanced-stage cancer. *Arch. Gen. Psychiatry* **68**, 71–78 (2011).

98. Gasser, P. *et al.* Safety and efficacy of lysergic acid diethylamide-assisted psychotherapy for anxiety associated with life-threatening diseases. *J. Nerv. Ment. Dis.* **202**, 513–520 (2014).

99. Griffiths, R. R. *et al.* Psilocybin produces substantial and sustained decreases in depression and anxiety in patients with life-threatening cancer: A randomized double-blind trial. *J. Psychopharmacol.* **30**, 1181–1197 (2016).

100. Ross, S. *et al.* Rapid and sustained symptom reduction following psilocybin treatment for anxiety and depression in patients with life-threatening cancer: a randomized controlled trial. *J. Psychopharmacol.* **30**, 1165–1180 (2016).

101. Davis, A. K. *et al.* Effects of Psilocybin-Assisted Therapy on Major Depressive Disorder: A Randomized Clinical Trial. *JAMA psychiatry* **78**, 481–489 (2021).

102. Carhart-Harris, R. *et al.* Trial of Psilocybin versus Escitalopram for Depression. *N. Engl. J. Med.* **384**, 1402–1411 (2021).

103. Goodwin, G. M. *et al.* Single-Dose Psilocybin for a Treatment-Resistant Episode of Major Depression. *N. Engl. J. Med.* **387**, 1637–1648 (2022).

104. Bogenschutz, M. P. *et al.* Percentage of Heavy Drinking Days Following Psilocybin-Assisted Psychotherapy vs Placebo in the Treatment of Adult Patients With Alcohol Use Disorder: A Randomized Clinical Trial. *JAMA psychiatry* **79**, 953–962 (2022).

105. von Rotz, R. *et al.* Single-dose psilocybin-assisted therapy in major depressive disorder: A placebo-controlled, double-blind, randomised clinical trial. *EClinicalMedicine* **56**, 101809 (2023).

106. Bouso, J. C., Doblin, R., Farré, M., Alcázar, M. A. & Gómez-Jarabo, G. MDMA-assisted psychotherapy using low doses in a small sample of women with chronic posttraumatic stress disorder. *J. Psychoactive Drugs* **40**, 225–236 (2008).

107. Mithoefer, M. C., Wagner, M. T., Mithoefer, A. T., Jerome, L. & Doblin, R. The safety and efficacy of {+/-}3,4-methylenedioxymethamphetamine-assisted psychotherapy in subjects with chronic, treatment-resistant posttraumatic stress disorder: the first randomized controlled pilot study. *J. Psychopharmacol.* **25**, 439–452 (2011).

108. Oehen, P., Traber, R., Widmer, V. & Schnyder, U. A randomized, controlled pilot study of MDMA (± 3,4-Methylenedioxymethamphetamine)-assisted psychotherapy for treatment of resistant, chronic Post-Traumatic Stress Disorder (PTSD). *J. Psychopharmacol.* **27**, 40–52 (2013).

109. Pacey, I. Randomized, Double-blind, Controlled of MDMA-assisted Psychotherapy in 12 Subjects With PTSD. *NCT01958593. Lykos Therapeutics* https://clinicaltrials.gov/study/NCT01958593 (2017).

110. Mithoefer, M. C. *et al.* MDMA-assisted psychotherapy for treatment of PTSD: study design and rationale for phase 3 trials based on pooled analysis of six phase 2 randomized controlled trials. *Psychopharmacology (Berl).* **236**, 2735–2745 (2019).

111. Danforth, A. L. *et al.* Reduction in social anxiety after MDMA-assisted psychotherapy with autistic adults: a randomized, double-blind, placebo-controlled pilot study. *Psychopharmacology (Berl).* **235**, 3137–3148 (2018).

112. Ot’alora G, M. *et al.* 3,4-Methylenedioxymethamphetamine-assisted psychotherapy for treatment of chronic posttraumatic stress disorder: A randomized phase 2 controlled trial. *J. Psychopharmacol.* **32**, 1295–1307 (2018).

113. Jerome, L. *et al.* Long-term follow-up outcomes of MDMA-assisted psychotherapy for treatment of PTSD: a longitudinal pooled analysis of six phase 2 trials. *Psychopharmacology (Berl).* **237**, 2485–2497 (2020).

114. Mitchell, J. M. *et al.* MDMA-assisted therapy for severe PTSD: a randomized, double-blind, placebo-controlled phase 3 study. *Nat. Med.* **27**, 1025–1033 (2021).

115. Ponte, L. *et al.* Sleep Quality Improvements After MDMA-Assisted Psychotherapy for the Treatment of Posttraumatic Stress Disorder. *J. Trauma. Stress* **34**, 851–863 (2021).

116. Brewerton, T. D., Gavidia, I., Suro, G. & Perlman, M. M. Eating disorder patients with and without PTSD treated in residential care: discharge and 6-month follow-up results. *J. Eat. Disord.* **11**, 48 (2023).

117. Nicholas, C. R. *et al.* The effects of MDMA-assisted therapy on alcohol and substance use in a phase 3 trial for treatment of severe PTSD. *Drug Alcohol Depend.* **233**, 109356 (2022).

118. Wolfson, P. E. *et al.* MDMA-assisted psychotherapy for treatment of anxiety and other psychological distress related to life-threatening illnesses: a randomized pilot study. *Sci. Rep.* **10**, 20442 (2020).

119. Mitchell, J. M. *et al.* MDMA-assisted therapy for moderate to severe PTSD: a randomized, placebo-controlled phase 3 trial. *Nat. Med.* **29**, 2473–2480 (2023).

120. Isbell, H. Comparison of the reactions induced by psilocybin and LSD-25 in man. *Psychopharmacologia* **1**, 29–38 (1959).

121. Shirvaikar, R. V & Kelkar, Y. W. Therapeutic trial of lysergic Acid diethylamide (LSD) and thioridazine in chronic schizophrenia. *Neurol. India* **14**, 97–101 (1966).

122. Smart, R. G., Storm, T., Baker, E. F. & Solursh, L. A controlled study of lysergide in the treatment of alcoholism. 1. The effects on drinking behavior. *Q. J. Stud. Alcohol* **27**, 469–482 (1966).

123. HOLLISTER, L. E. O. E., SHELTON, J. & KRIEGER, G. A Controlled Comparison of Lysergic Acid Diethylamide (LSD) and Dextroamphetamine in Alcoholics. *Am. J. Psychiatry* **125**, 1352–1357 (1969).

124. Tomsovic, M. & Edwards, R. V. Lysergide treatment of schizophrenic and nonschizophrenic alcoholics: a controlled evaluation. *Q. J. Stud. Alcohol* **31**, 932–949 (1970).

125. Strassman, R. J., Qualls, C. R., Uhlenhuth, E. H. & Kellner, R. Dose-response study of N,N-dimethyltryptamine in humans. II. Subjective effects and preliminary results of a new rating scale. *Arch. Gen. Psychiatry* **51**, 98–108 (1994).

126. Schmid, Y. *et al.* Acute Effects of Lysergic Acid Diethylamide in Healthy Subjects. *Biol. Psychiatry* **78**, 544–553 (2015).

127. Preller, K. *et al.* Changes in global and thalamic brain connectivity in LSD-induced altered states of consciousness are attributable to the 5-HT2A receptor. *Elife* **7**, (2018).

128. Wießner, I. *et al.* LSD, madness and healing: Mystical experiences as possible link between psychosis model and therapy model. *Psychol. Med.* **53**, 1151–1165 (2023).

129. Vallersnes, O. M. *et al.* Psychosis associated with acute recreational drug toxicity: a European case series. *BMC Psychiatry* **16**, 293 (2016).
